# Ancient mtDNA diversity reveals specific population development of wild horses in Switzerland after the Last Glacial Maximum

**DOI:** 10.1371/journal.pone.0177458

**Published:** 2017-05-24

**Authors:** Julia Elsner, Michael Hofreiter, Jörg Schibler, Angela Schlumbaum

**Affiliations:** 1 Integrative Prehistory and Archaeological Science, University of Basel, Basel, Switzerland; 2 Institute for Biochemistry and Biology, University of Potsdam, Potsdam, Germany; University of Florence, ITALY

## Abstract

On large geographical scales, changes in animal population distribution and abundance are driven by environmental change due to climatic and anthropogenic processes. However, so far, little is known about population dynamics on a regional scale. We have investigated 92 archaeological horse remains from nine sites mainly adjacent to the Swiss Jura Mountains dating from c. 41,000–5,000 years BP. The time frame includes major environmental turning points such as the Last Glacial Maximum (LGM), followed by steppe vegetation, afforestation and initial re-opening of the landscape by human agricultural activities. To investigate matrilinear population dynamics, we assembled 240 base pairs of the mitochondrial d-loop. F_ST_ values indicate large genetic differentiation of the horse populations that were present during and directly after the LGM. After the retreat of the ice, a highly diverse population expanded as demonstrated by significantly negative results for Tajima’s *D*, Fu’s *F*_*S*_ and mismatch analyses. At the same time, a different development took place in Asia where populations declined after the LGM. This first comprehensive investigation of wild horse remains on a regional scale reveals a discontinuous colonisation of succeeding populations, a pattern that diverges from the larger Eurasian trend.

## Introduction

Population distributions and abundance patterns of species are driven by environmental change. Natural and anthropogenic processes impact the availability of food resources for large herbivores, but species respond individually to these challenges (e.g., [1, 2]). Generally, large animals are more likely to react to climate change due to longer generation intervals and smaller effective population size [3]. The Pleistocene-Holocene transition is characterised by profound climatic and thus environmental change, and species well adapted to the open steppe vegetation in Eurasia and North America were confronted with fragmentation and even loss of habitat, yet some were able to establish new niches and survived [1, 4]. A combination of human hunting pressure and habitat fragmentation caused by climate change seems to be the most appropriate explanation for most extinction events in the Old World [5–8] and is supported by both archaeological and palaeo-climatic evidence.

One of the key species of the Pleistocene steppe was the horse, *Equus ferus caballus* L. 1758. The palaeontological and archaeological record shows its abundance in an area ranging from North America to southern Europe. In the first genetic studies featuring Pleistocene horses from North America and Eurasia, it emerged that in addition to a mitochondrial clade consisting of North American horses, a second clade included both *E*. *f*. *caballus* from North America and Eurasia, and domestic horses [9, 10]. Cieslak *et al*. [11] further showed that while some matrilines were regionally confined to Alaska, the Eurasian steppe, and Iberia, others were extremely widely distributed suggesting a panmictic population. The approach of Lorenzen *et al*. [1] focused on (amongst others) horse population development in response to climate change, habitat distribution and human encroachment on a global scale, mainly featuring specimens from north-eastern Asia and north-western America. A positive correlation between available habitat size and genetic diversity supports their conclusion that climate had been the major driving force in population changes over the past 50 thousand years (ka). Horses were thriving particularly under cold and arid conditions. The authors found, however, that the drastic decline of genetic diversity in horses after the Last Glacial Maximum (LGM) could not be explained by habitat reduction alone and thus might reflect the impact of expanding human populations in Eurasia as indicated by the prevalence of horse remains in the archaeological (not palaeontological) record [1]. This scenario was supported by Orlando *et al*. [12], who found support for models indicating population reduction in the interglacial phases and expansion during the cold stages of Marine Isotope Stages (MIS) 4 and 3, followed by a 100-fold collapse after the LGM. In contrast to these large scale developments, little is as yet known about the effect of environmental change on regional horse populations, and it is probable that they responded more variably, depending on local conditions.

To investigate this issue, we focused on local horse population development through the course of c. 50 ka in the heterogeneous landscape between and including the Alps and the Jura Mountain Chain—present day Switzerland. This region was subjected to sometimes rapid environmental change. Both Alpine and Jura glaciers reached their maximal extent at c. 25 ka BP and started to retreat between 22 and 21 ka BP [13–15]. Deglaciation progressed quite rapidly; the northern Jura c. 50 km south of Basel (Rhine knee area) was ice-free by 19 ka BP [16], while around 18 ka BP, soil development had started in the Alpine foreland [17] when 80% of the LGM ice had melted [18]. Environmental conditions improved rapidly and human (temporary) settlements, first at the foot of the mountains and in caves, later towards the lakes [19], became more numerous [20]. Palynological data indicate herbaceous, heliophilous vegetation until c. 14.7 ka BP, forming grassland interspersed with dwarf shrubs [20–22]. At 14.7 ka BP, mean temperature rose by c. 5°C [21] and in the course of the following 2,000 years the landscape turned into open woodland; by 11 ka BP, forest canopy was probably closed [23]. Anthropogenic influence (agricultural activity) becomes traceable around 7 ka BP in the Jura [21] and in the Alpine foreland [24]. Since the Neolithic, large game species have lost significance in human diets [25], but since agriculture and domestic animal husbandry demanded open landscapes, wild species were increasingly displaced. Horses are absent from the Mesolithic archaeological record in Switzerland despite numerous known sites (yet less than from earlier and later periods) which include faunal assemblages [19]. Most likely, the last wild horses in Switzerland stem from Neolithic lakeshore settlements where horses are present in very low amounts; however, it cannot be ultimately ruled out that they represent first domestics [26]. It is assumed that from the Bronze Age onwards, all horse remains stem from domestic animals [27].

**Fig 1:**
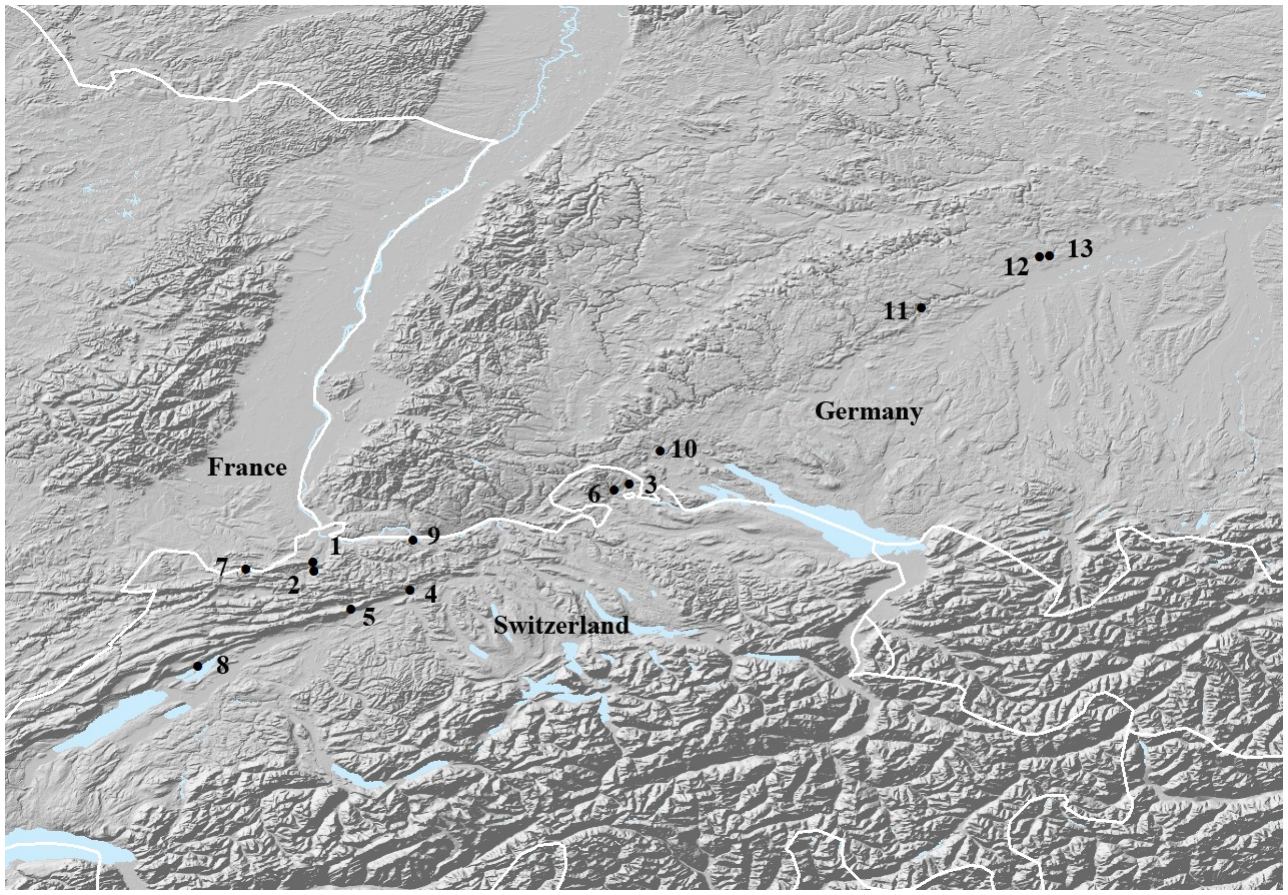
Map of investigated sites in Switzerland and added sites in Germany. Site numbers according to Table 1.

We have investigated horse teeth and bones from nine sites mainly adjacent to the Swiss Jura Mountains dating from c. 41 to 5 ka BP (Fig 1, Table 1, S1 Table). The dataset comprises all known Pleistocene sites with more than one horse remain. To address matrilinear population dynamics of Upper Pleistocene and Holocene wild horses, we have assembled 240 base pairs (bp) of the mitochondrial d-loop. We aim to contextualise population developments and natural and anthropogenic changes of the environment in a fringe area of the Eurasian steppe biota. These results are compared with published data from Northern Asia and the Ural region, the heartland of the steppe.

**Table 1:** Details of the investigated sites, number of samples and haplogroups. Archaeological cultures according to Leesch [28]. Mitochondrial haplogroup nomenclature follows Cieslak *et al*. [11]. Haplogroups identified from directly dated specimens are indicated in italics. For individual dates, skeletal elements and references see S1 Table.

Name of site		^14^C Age (cal ka BP)	Archaeological culture	Number of positive samples	Mitochondrial haplogroups
**1**	Schalberghöhle	41–37	-	3	*A*; *X3*
		12.5	Azilian	1	*B*
**2**	Kohlerhöhle	24–23	Badegoulian	11	B; H; *X3*
		15–13.5	Magdalenian	12	*A*; *B*; D; K
**3**	Kesslerloch	18–14	Magdalenian	43	*A*; *B*; D; *H*; *K*; X3; X4b
**4**	Käsloch	17–15	Magdalenian	3	*B*; *H*
**5**	Rislisberghöhle	15–12.5	Magdalenian—Azilian	4	*K*
**6**	Schweizersbild	15–14	Magdalenian	6	*A*; *B*; *X3*
**7**	Abri Neumühle	14.5	Magdalenian	1	*C*
**8**	Twann-Bahnhof	5.5	Neolithic	6	A; *B*; D
**9**	Mumpf	5	Neolithic	2	*D*
**10**	Petersfels^a^^,^ ^b^	15–14	Magdalenian	2	*A*; *B*
**11**	Hohlefels^a^	14.8	Magdalenian	1	*C*
**12**	Bocksteinhöhle^a^	50	-	1	*A*
**13**	Vogelherdhöhle^a^	17	Magdalenian	1	*A*
				total = 97	

^a^ Weinstock *et al*. [10];

^b^ Lorenzen *et al*. [1]

## Materials and methods

### Archaeological samples

A total of 202 horse (*Equus* sp.) teeth and bones were sampled either from Palaeolithic or Neolithic anthropogenic cultural layers, or from palaeontological contexts associated with hyena hunting activity and chance finds; 92 of them yielded amplifiable mtDNA [29]. All sites are located in or close to the Jura Mountains in Switzerland (Fig 1, Table 1, S1 Table). The samples had been stored in museums and archaeological collections since their excavations. To obtain direct dates from each layer at the respective sites, 31 samples were chosen for ^14^C dating using accelerator mass spectrometry (AMS) at ETH Zurich, Switzerland, and calibrated with CalPal [30]. Additional ^14^C dates were assembled from the literature. In some cases the age was projected from dendrochronology or typology (S1 Table). Five ^14^C dated sequences of Pleistocene horse remains (DQ007558/DQ007611: 14’751 cal BP, DQ007556/DQ007609: 14’752 cal BP, DQ007591: 16’928 cal BP, DQ007590: 50’735 cal BP; [10] FJ204352: 14’500 cal BP; [1] from the Swabian Jura were added to the dataset, resulting in 97 specimens to be analysed.

### Processing of ancient samples

Preparation, extraction, amplification and Sanger sequencing of ancient samples were performed as described in Elsner *et al*. [29] in dedicated ancient DNA facilities following established standards for aDNA work [31], including multiple independent extractions and PCR, and routine cloning. Mitochondrial d-loop positions 15,492 to 15,669 and 15,696 to 15,758 [32] were targeted in seven partially overlapping fragments [33]. Contamination of ancient samples was never detected; all PCR amplification products in the extraction and PCR blank controls came from microorganisms or were unidentifiable according to GenBank Blast search and are most likely explained by the permissive PCR set up (low annealing temperature, up to 70 cycles).

### Data analysis

Sequences were edited and aligned by eye with BioEdit [34]. A consensus sequence was built from at least three amplifications from a minimum of two independent extractions based on majority. To deal with sequences with missing nucleotides on the one hand, and to avoid specimens that stemmed from potentially mixed-up layers and an overrepresentation of Magdalenian samples, the analyses were done on three datasets (Table 2).

**Table 2:** Datasets used for analyses.

	Selection criterion	Number of sequences						
		Total	Palaeontological	Badegoulian	Madgalenian	Magd.+Azilian	Azilian	Neolithic
Dataset 1	all samples	**97**	4	11	70	74	4	8
Dataset 2	samples with > 40% missing nucleotides excluded	**78**	4	11	53	57	4	6
Dataset 3	only ^14^C dated samples	**36**	4	5	20	-	3	4

Within the datasets, samples were assembled into time bins according to similar environmental conditions. Magdalenian and Azilian samples were handled both in combination and separately because, on the one hand, the transition between the cultural horizons is marked by the temperature increase at c. 14.7 ka BP, but, on the other hand, possibly mixed up layers within the sites may have led to mis-assignment of individual specimens. The combined category could thus be dropped for dataset 3. Both Azilian and Neolithic date into the early Holocene, yet we assume different dynamics in the Neolithic due to anthropogenic interference to the landscape leading to a dilution of the interdependence of latitude/temperature and vegetation, and thus did not combine those samples.

Nucleotide diversity is defined as the average number of nucleotide differences per site between two DNA sequences in all possible pairs in the population studied, while haplotype diversity is a way to describe the uniqueness of a haplotype in a population. These indices as well as F_ST_ values and tests to detect recent population expansion (Tajima’s *D*, Fu’s *FS*, sum of squared deviations SSD, Harpending’s raggedness index) were computed with Arlequin 3.5 [35] with missing data coded as ‘?’ and allowed level of missing data set to 5%. Tajima’s *D* [36] uses the mean average number of pairwise nucleotide differences and the number of segregating sites, each scaled so that they are expected to be the same in a neutrally evolving population of constant size; Fu’s *F*_*S*_ [37] is based on the number of alleles (haplotypes). It is generally assumed that Fu’s *F*_*S*_ is more sensitive in detecting population expansion than Tajima’s *D*. The raggedness index [38, 39] is also used to detect recent population expansions, which is rejected by non-significant results. The SSD between observed and expected mismatch (distribution of the number of sequence differences) quantifies the smoothness of the observed mismatch.

To reject a statistical bias in the analyses introduced by uneven sample sizes in the respective time bins, directly compared time bins (e.g. Magdalenian and LGM) were randomized (10k permutations with replacement) using nucleotide and haplotype diversity estimated with the packages *pegas* [40] and *seqinR* [41] implemented in R [42] using the option ‘pairwise deletion of missing data’. This was done by pooling the sequences of two time bins, repeatedly creating two pseudo-groups of the same size of the original bins from the pool and collecting diversity parameters from them. In case the combined pseudo diversity deviated from the original diversity (threshold 0.05), sampling bias has to be assumed.

For the construction of Median Joining Networks (MJN) [43] with the program Network (fluxus-engineering.com) polymorphic nucleotide positions were down-weighted according to the number of polymorphisms (default 50) [33]. The transition: transversion weight was set to 1: 10 (S2 Table). Principal component analysis (PCA) was based on relative haplogroup frequencies within the Eurasian dataset and computed with PAST [44].

## Results

### Swiss and Swabian wild horse populations

**Fig 2:**
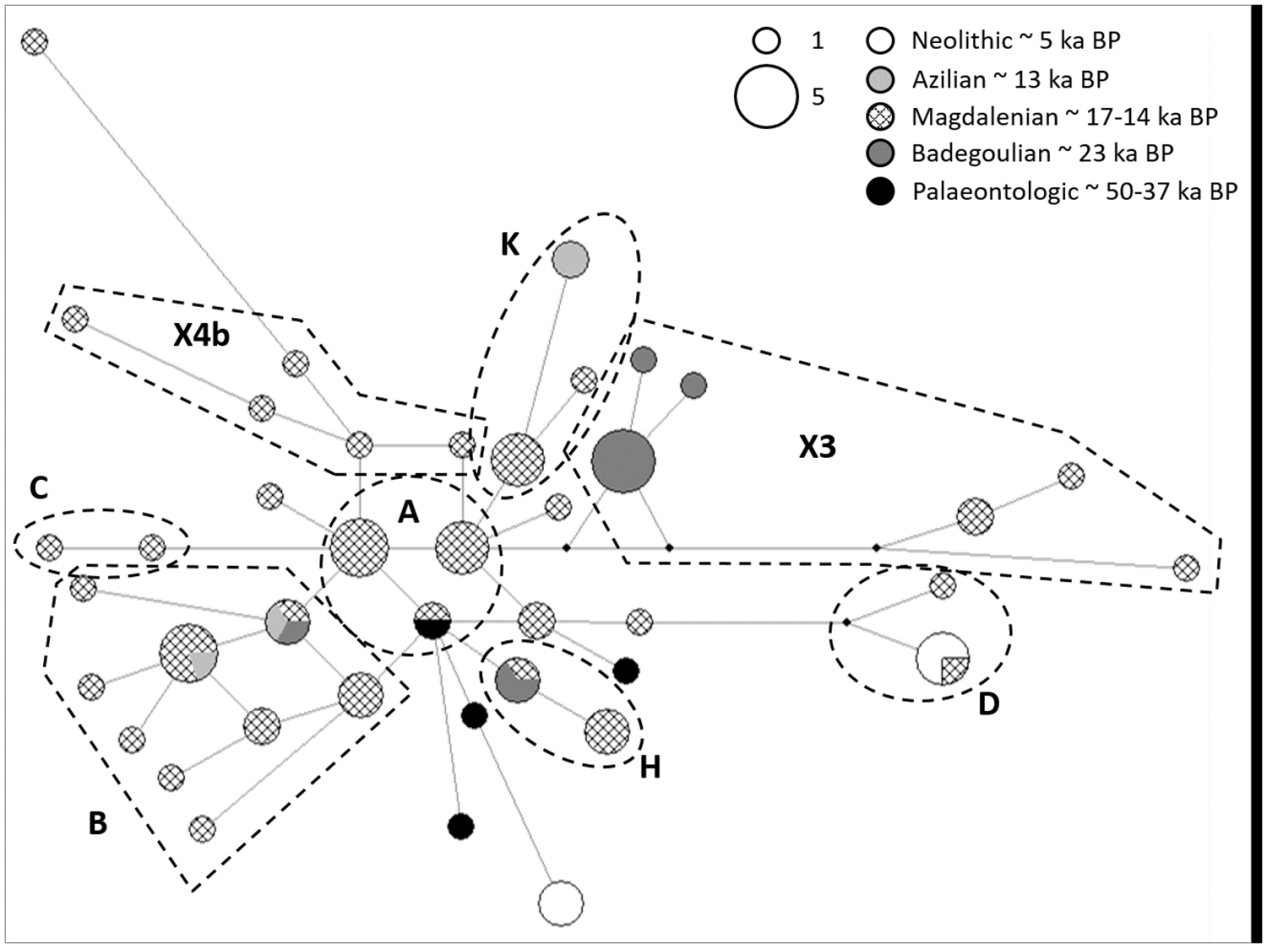
Median Joining Network (MJN) of horse populations (97 samples, max. 241 bp). Nodes are proportional to frequencies and branch length according to number of substitutions. Haplogroup nomenclature follows Cieslak *et al*. [11].

The maximal sequence length of 241 bp (without primers) could be assembled for 50 specimens, 231 bp for 15 samples. The remaining 27 specimens had missing data mostly between positions 15,564 and 15,669 relative to the horse reference mitogenome sequence [32]. Amongst the 92 Swiss and five Swabian horse samples, 36 polymorphic sites were present resulting in 41 haplotypes (ht), which can be summarized into eight haplogroups (hg) according to Cieslak *et al*. [11]: A, B, C, D; H, K and X3 (Fig 2; S3 Table). One previously unnamed haplogroup was labelled X4b following [11], distinguished by nucleotide positions 15495, **15540**, 15602, (**15718**) and 15720 (defining nucleotide positions in bold, optional position in parenthesis).

Nucleotide and haplotype diversities are shown in Table 3 (see also S4 Table). Both nucleotide and haplotype diversities are highest in the Magdalenian and lowest in the Badegoulian. Note that nucleotide diversity of the Neolithic deme is relatively high compared to the lower haplotype diversity, indicating population fragmentation.

**Table 3:** Nucleotide and haplotype diversities in horse populations from Switzerland and the Swabian Jura (dataset 2).

Time period	Number of samples	Number of haplotypes	Nucleotide diversity	Haplotype diversity
Palaeontological	4	4	0.0104	1
Badegoulian	11	4	0.0093	0.6
Magdalenian	53	28	0.016	0.95
Magd. + Azilian	57	29	0.0159	0.95
Azilian	4	3	0.0104	0.83
Neolithic	6	2	0.0149	0.6

**Fig 3:**
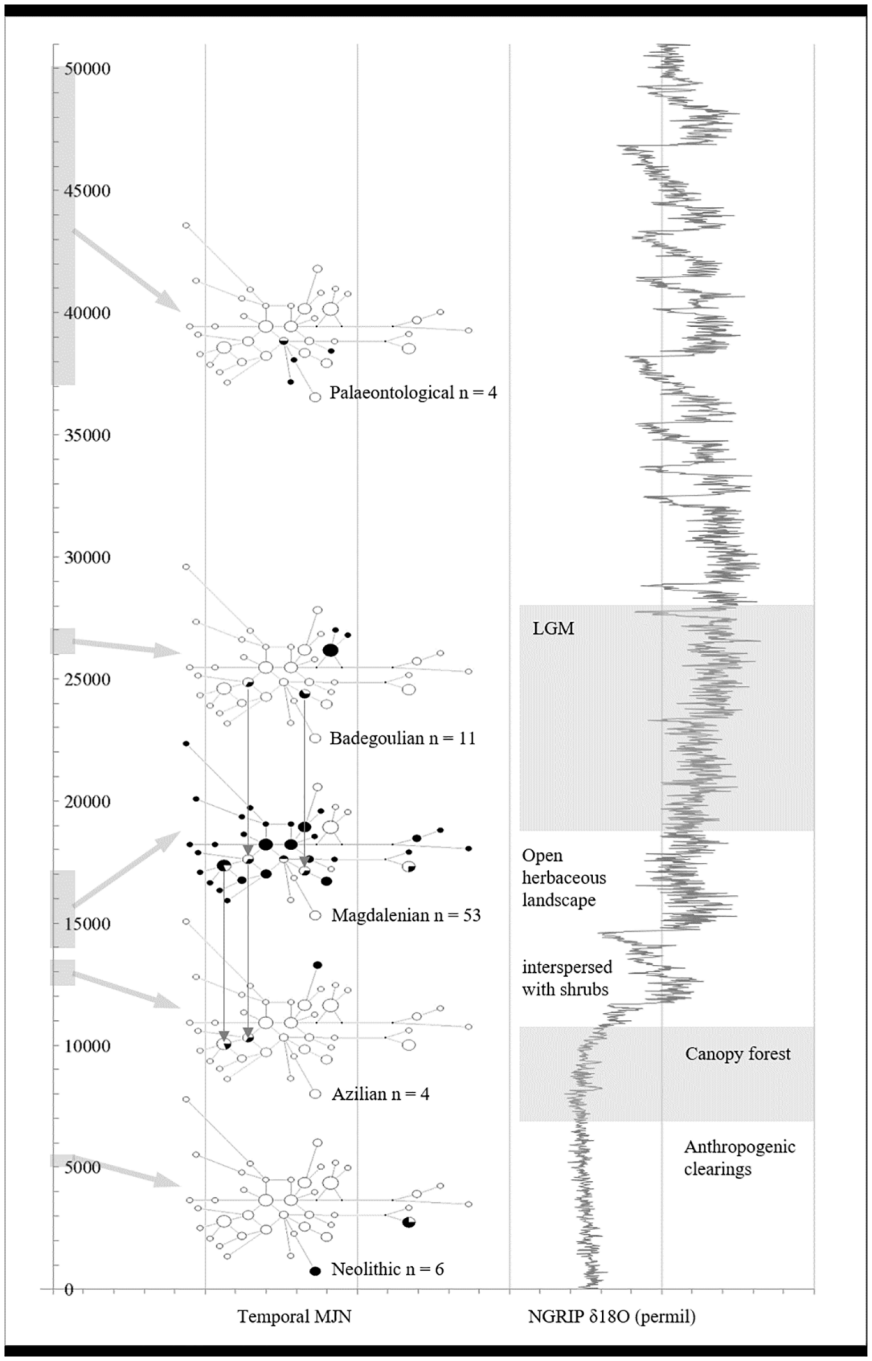
Temporal Median Joining Networks (MJN) of Swiss and Swabian horse populations (97 samples, max. 241 bp) in the context of environmental conditions and temperature. Light grey boxes and arrows show the age of the sequences the particular MJN is based on. Empty nodes represent haplotypes absent from particular time bin. Vertical arrows indicate continuity of haplotypes from subsequent time bins. δ^18^O record of the North Greenland Ice Core Project (NGRIP) after [49–53], vegetation data from [21, 54].

The Median Joining Network (241 bp) shows only little haplotype continuity between the time bins. Two lineages (hgs B and H) are found in Switzerland during and after the LGM, and two lineages of hg B continue into the Azilian (Figs 2 and 3). All other haplotypes are restricted to single time bins or at least do not occur in succeeding time bins. For reasons of comparability, we tested whether a bias was introduced by uneven sample sizes within the time bins. This can be rejected for the comparison between Magdalenian (+ Azilian) and Badegoulian by permutation testing (S1 Fig). F_ST_ values for these time bins portend great genetic differentiation (0.17, *p* < 0.001) (S5 Table).

Simulations according to Tajima [36] and Fu [37] indicate recent population expansion for the Magdalenian and the combined Magdalenian and Azilian sample sets (Table 4, S6 Table). This is supported by the distribution of the number of sequence differences between haplotypes (mismatch analysis, S2 Fig). Both the palaeontological and Magdalenian (+ Azilian) bins exhibit unimodal distributions. Equally, neither the sum of the squared deviations (SSD) nor Harpending’s raggedness index [38, 39] were statistically significant, further indicating population expansion (Table 4). This applies for the Badegoulian and Azilian time bins as well. However, an increased SSD and raggedness as well as multimodal distribution point to populations with secondary contact, i.e. populations that have received genetic input from further populations [45]. For the Neolithic time bin, population expansion can be rejected explicitly; the very high SSD and raggedness index might even indicate that individuals from the same site stemmed originally from independent populations.

**Table 4:** Tajima’s *D*, Fu’s *F*_*S*_, sum of squared deviations (SSD) and Harpending’s raggedness index results for horse populations from Switzerland and the Swabian Jura (dataset 2). Significant results are in bold.

Time period	Tajima’s *D*	*p*	Fu’s *F*_*S*_	*p*	SSD	*p*	Raggedness index	*p*
Palaeontological	-0.8	.2	-1.51	.06	0.02	0.8	0.1	0.9
Badegoulian	-0.09	.5	0.77	.7	0.13	0.1	0.3	0.1
Magdalenian	-1.32	.07	**-21.71**	0	0.001	0.6	0.02	0.6
Magd. + Azilian	-1.29	.08	**-22.59**	0	0.001	0.5	0.02	0.6
Azilian	1.37	.9	0.46	.5	0.09	0.3	0.25	0.7
Neolithic	2.12	1	4.51	1	**0.38**	0.02	**0.88**	0.03

In Fig 3, a temporal Median Joining Network (MJN) of 97 sequences (up to 241 bp) from the Swiss and Swabian Jura is put into context with the δ^18^O record of the North Greenland Ice Core Project (NGRIP) and prevailing regional environment. During the LGM, three distinct haplogroups are present (all from one site), two of which are also found in the Magdalenian. Most lineages present during the LGM did not reoccur in later time periods. The dominating haplotype X3 has as yet not been detected in Eurasian Pleistocene horses [1, 10–12] but is frequent in some modern breeds, both in Iberia (e.g. [46]) and Asia (e.g. [47, 48]). During the Magdalenian, when an open herbaceous landscape prevailed, the network topology shows a star-like expansion pattern. After c. 14.7 ka BP when global atmospheric temperature rose by c. 5°C and forestation began, only two genetically distant lineages are recovered from the Azilian. By c. 12 ka BP temperatures levelled off at present day conditions. Two distinct matrilines, one of which occurred in the Magdalenian, are present in the Neolithic when early human impact on the environment is observed. Generally, very little continuity existed through time.

### Comparison with Pleistocene horse sequences from across Eurasia

**Fig 4:**
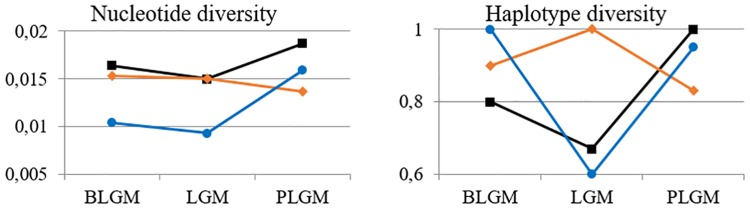
Nucleotide (left panel) and haplotype (right panel) diversity in Asia (black squares), Ural region (red diamonds) and the Swiss and Swabian Jura (blue circles) before (BLGM), during (LGM) and after (PLGM) the Last Glacial Maximum.

**Fig 5:**
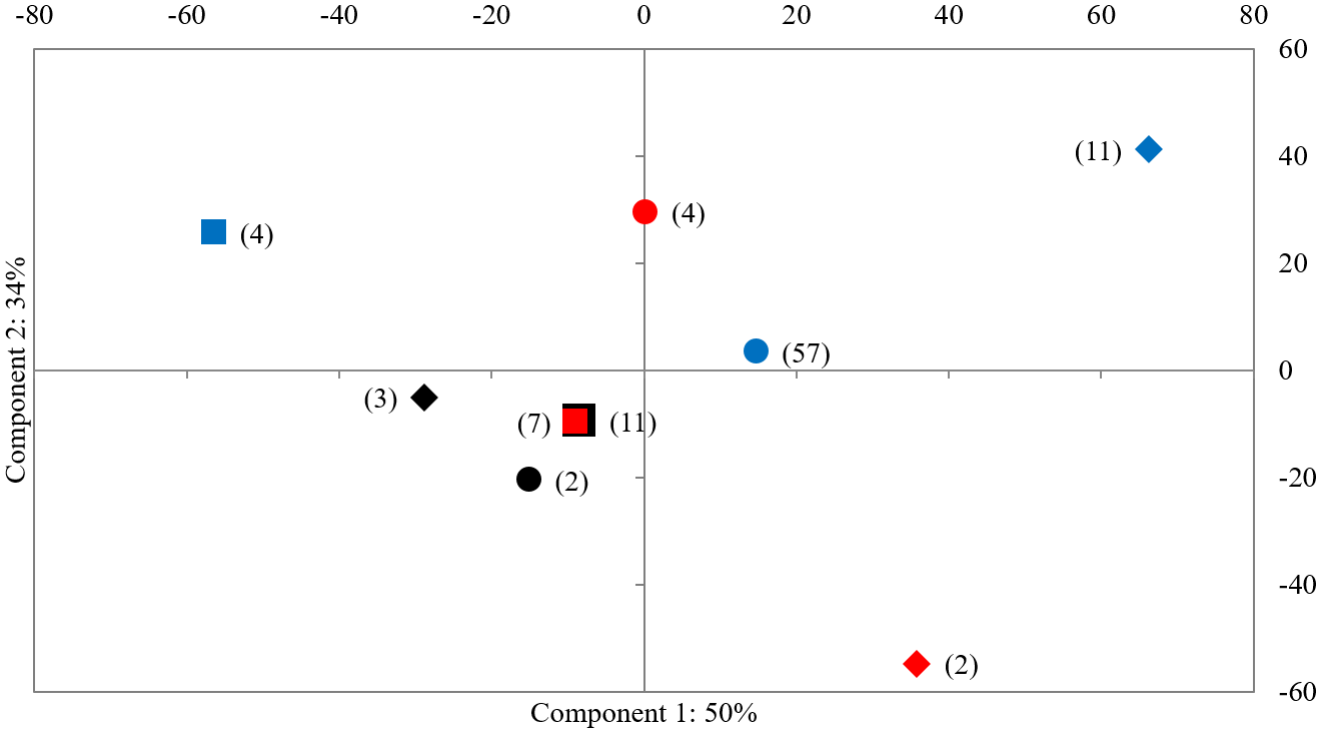
PCA graph based on relative haplogroup frequencies. Asia (black), Ural region (red) and Swiss/Swabian (blue) samples divided in time bins: square = before the LGM, diamond = LGM, circle = after the LGM. Sample numbers are given in parenthesis. The first two components explain 84% of the variation (see S4 Fig for loadings).

The Swiss and Swabian Jura sequences were put into context with published samples from Northern Asia and the Urals regions (S7 Table). They were sorted into time bins: before the LGM (BLGM, c. 50–27.5 ka BP), during the LGM (LGM, c. 25–22.5 ka BP), and after the LGM (PLGM, c. 18–12.5 ka BP). Nucleotide diversity is highest in Asia at all times, and is decreasing westwards (Fig 4). During the LGM, it drops slightly and rises to higher level afterwards in Asia and Switzerland, yet decreases in the Ural region. Haplotype diversity has to be regarded with caution due to low sample sizes (see Fig 5, S7 Table). Generally the trend seen from nucleotide diversity is repeated (Fig 4, S8 Table).

Because the sample sizes in the Eurasian datasets are low compared to the Swiss/Swabian Jura, an unbiased comparison of F_ST_ values is only possible for the time bins Swiss/Swabian LGM and Swiss/Swabian PLGM (see above) and Swiss/Swabian LGM and Asian BLGM (F_ST_ 0.23, p < 0.001; S3 Fig, S9 Table). A principal component analysis (PCA) based on relative haplogroup frequencies demonstrates that while the Asian samples are genetically close to each other through time, and might, together with the Ural BLGM lineages, be addressed as panmictic, the Swiss/Swabian samples are more distinct. Moreover, the interruptive nature of the LGM concerning population continuity is apparent as the LGM demes from all regions stand apart from the preceding and succeeding populations (Fig 5, S4 Fig).

## Discussion

The investigation of mt d-loop sequences from 97 horse teeth and bones dating from 50 to 5 ka BP from the region of the Swiss and Swabian Jura is the first regional study of Pleistocene to early Holocene horse population development. The investigated time frame includes the LGM as a major environmental change, which was followed by open steppe and later afforestation after the temperature rise c. 14.7 ka BP, leading to a densely forested landscape which was in turn partially re-opened for humans’ agricultural demands. The archaeological context of the horse remains studied here mirror hunter-gatherer colonisation, settlement and hunting strategies in the region. We have investigated all known *E*. *f*. *caballus* remains from Pleistocene Switzerland; open-air dry- and wetland sites did not contain specimens with amplifiable mtDNA [29].

In the region of the Swiss and Swabian Jura, we observe only little continuity of horse matrilines through time, particularly in the populations present during (Badegoulian) and directly after (Magdalenian) the LGM. Horses were most abundant when the landscape was open and comprised the typical characteristics of steppe biota like herbaceous grassland interspersed with shrub flora. Statistical analyses portend population expansion in the Magdalenian. Despite numerous archaeological sites from the Azilian, the Mesolithic and the Neolithic, horse finds from these horizons are extremely rare [19, 26]. Population fragmentation is indicated by comparatively high nucleotide and low haplotype diversity, and for the Neolithic deme, expansion can be rejected based on a significant raggedness index.

Throughout the investigated time frame, diversity patterns in Eurasia change. The general trend of decreasing nucleotide and haplotype diversity from east to west supports models of an initial population expansion of wild horses in eastern Asia [55]. However, the idea of a panmictic horse population across Eurasia during the last 50 ka (e.g. [1, 11]) has to be challenged, even for pre-LGM times, based on relative haplogroup frequencies. During the LGM, when large parts of the continent were covered by ice or un-inhabitable due to extremely cold and arid conditions [56], populations were isolated and fragmented as partially supported by F_ST_ values. Subsequently, this led to a diversification particularly in the Ural and Swiss/Swabian Jura regions. The finding of a regional population expansion in the Magdalenian contradicts previous studies of horse population development [1, 12] that proposed a rapid decline directly after the LGM.

Wild horses might have gone extinct in the region of present-day Switzerland in the Neolithic, yet due to low numbers of remains in the archaeological record from the Azilian onwards it is challenging to trace this development genetically. It seems likely that climate, and not human activity, was the major driving force behind abundance and diversity of horse populations in this region: the expansion time is contemporaneous with intensified human encroachment of the area after the LGM. However, Neolithic land use for farming and domestic animal husbandry presumably replaced the last remnants of the wild horse population in Switzerland; genetically, this remains to be proven.

## Conclusion

In summary, the region of present-day Switzerland was inhabited by discontinuous horse populations and we cannot assume a panmictic deme over the investigated time period of 50 ka. Horse populations mostly replaced each other during and after the LGM, and only little continuity is observable. In the Magdalenian, diversity was highest as the population expanded into the newly accessible landscape. When the landscape transformed from open steppe into more and more densely forested woodland, the population probably shrunk and became fragmented.

Comparing horse matrilineages from Asia, the Ural, and the Swiss and Swabian Jura regions, population-specific developments are detectable. Wild horses possibly never formed a panmictic deme throughout their distribution range, and the LGM led to additional population fragmentation which subsequently persisted.

Besides the methodological challenges due to the discontinuous and unbalanced representation of equid sequences, this paper provides the first comprehensive investigation of wild horse remains from one restricted region. This approach has offered the opportunity to focus on aspects of horse population development that might be overlooked in the global picture by demonstrating specific reaction patterns to changing environmental conditions.

## Supporting information

S1 Fig Density plots of randomisation test (10,000 permutations with replacement) based on nucleotide (left panel) and haplotype (right panel) diversity for datasets 1–3. Unbiased and thus comparable pairs are framed. A: Dataset 1; B: Dataset 2; C: Dataset 3.
(DOCX)

S2 Fig Mismatch distribution (observed, bold line, and expected, dashed line) within time bins. X-axis: pairwise differences, y-axis: number of pairs. A: Dataset 1; B: Dataset 2; C: Dataset 3.
(DOCX)

S3 Fig Density plots of randomization of Eurasian Pleistocene horse sample groups (10 k permutations with replacement) based on nucleotide diversity. Swiss/Swabian samples: dataset 2. Rejections of null hypothesis are framed blue (Swiss LGM vs. Asia BLGM 0.0208; Swiss LGM vs. Swiss PLGM 0.034).
(DOCX)

S4 Fig Influential haplogroups (loadings) of component 1 (left panel) and 2 (right panel) for PCA graph. (DOCX)

S1 Table Details of investigated sites, including site context, main references, location, laboratory and archaeological code, skeletal element, GenBank accession code, and dates [extended from 29]. (DOCX)

S2 Table Parameters for weighting of nucleotide positions for Median Joining Network analysis based on 97 Pleistocene horse mitochondrial d-loop sequences. (DOCX)

S3 Table Details of haplogroups detected in Pleistocene horses from the Swiss and Swabian Jura region, nomenclature follows [2]. Haplogroup defining nucleotide positions relative to the horse reference mitogenome [1] are shown according to their position. All deviations from the reference sequence are given, with mandatory defining positions in bold and optional nucleotide positions in parenthesis. Note that transitions on nucleotide positions 15,585; 15,604 and 15,650 occur sporadically in all haplogroups; these positions are regarded as hotspots and therefore dismissed.
(DOCX)

S4 Table Nucleotide and haplotype diversities in horse populations from Switzerland and the Swabian Jura (all datasets). (DOCX)

S5 Table F_ST_ values of pairwise populations (all datasets). Lower triangle: F_ST_ values, upper triangle: *p* values. Comparable populations are boxed, significant F_ST_ values are in bold.
(DOCX)

S6 Table Tajima’s *D*, Fu’s *F*_*S*_, sum of squared deviances (SSD) and Harpending’s raggedness index results for horse populations from Switzerland and the Swabian Jura (all datasets). Significant results are in bold. NaN = not a number because only one haplotype was present.
(DOCX)

S7 Table Sequences of published Pleistocene horses from Eurasia. Sequences marked with an ‘^a^’ are part of draft full genomes which are obtainable as SRA-Illumina runs on GenBank.
(DOCX)

S8 Table Nucleotide and haplotype diversity of Eurasian Pleistocene horses based on pairwise deletion of missing nucleotides. (DOCX)

S9 Table F_ST_ values of Eurasian Pleistocene horses. Lower triangle: F_ST_ values, upper triangle: *p* values. Comparable populations are boxed, significant F_ST_ values are in bold. Swiss/Swabian samples: dataset 2.
(DOCX)
