# Supplementary material for: Ancient mtDNA diversity reveals specific population development of wild horses in Switzerland after the Last Glacial Maximum
Source: PLoS One. 2017 May 24;12(5):e0177458. doi: 10.1371/journal.pone.0177458 (PMC5443500; doi:10.1371/journal.pone.0177458)

S1 Fig: Density plots of randomisation test (10,000 permutations with replacement) based on nucleotide (left panel) and haplotype (right panel) diversity for datasets 1-3. Unbiased and thus comparable pairs are framed. A: Dataset 1; B: Dataset 2; C: Dataset 3.

S1 Fig A: Density plots of randomisation test (10,000 permutations with replacement) based on nucleotide (left panel) and haplotype (right panel) diversity. Dataset 1.


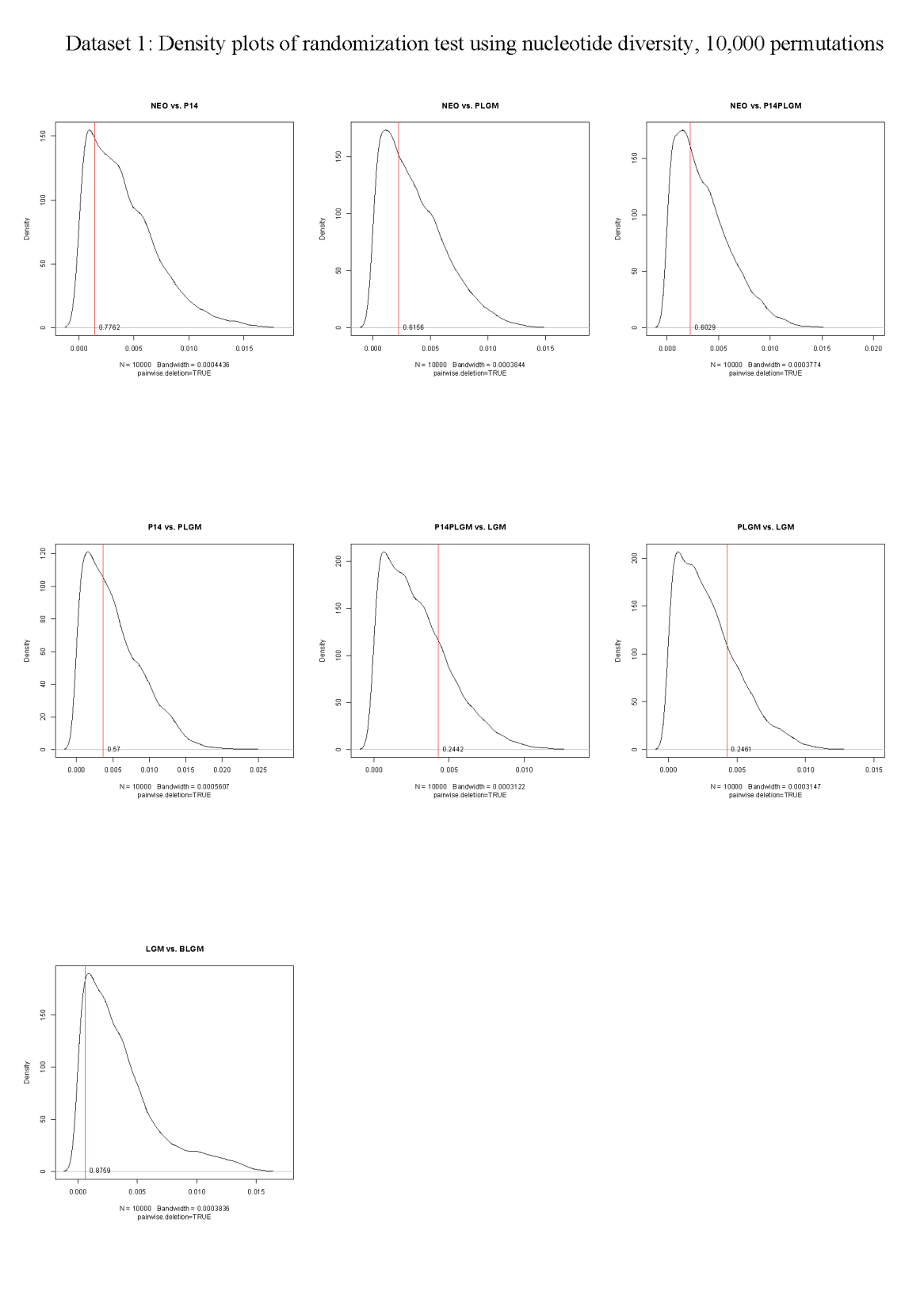

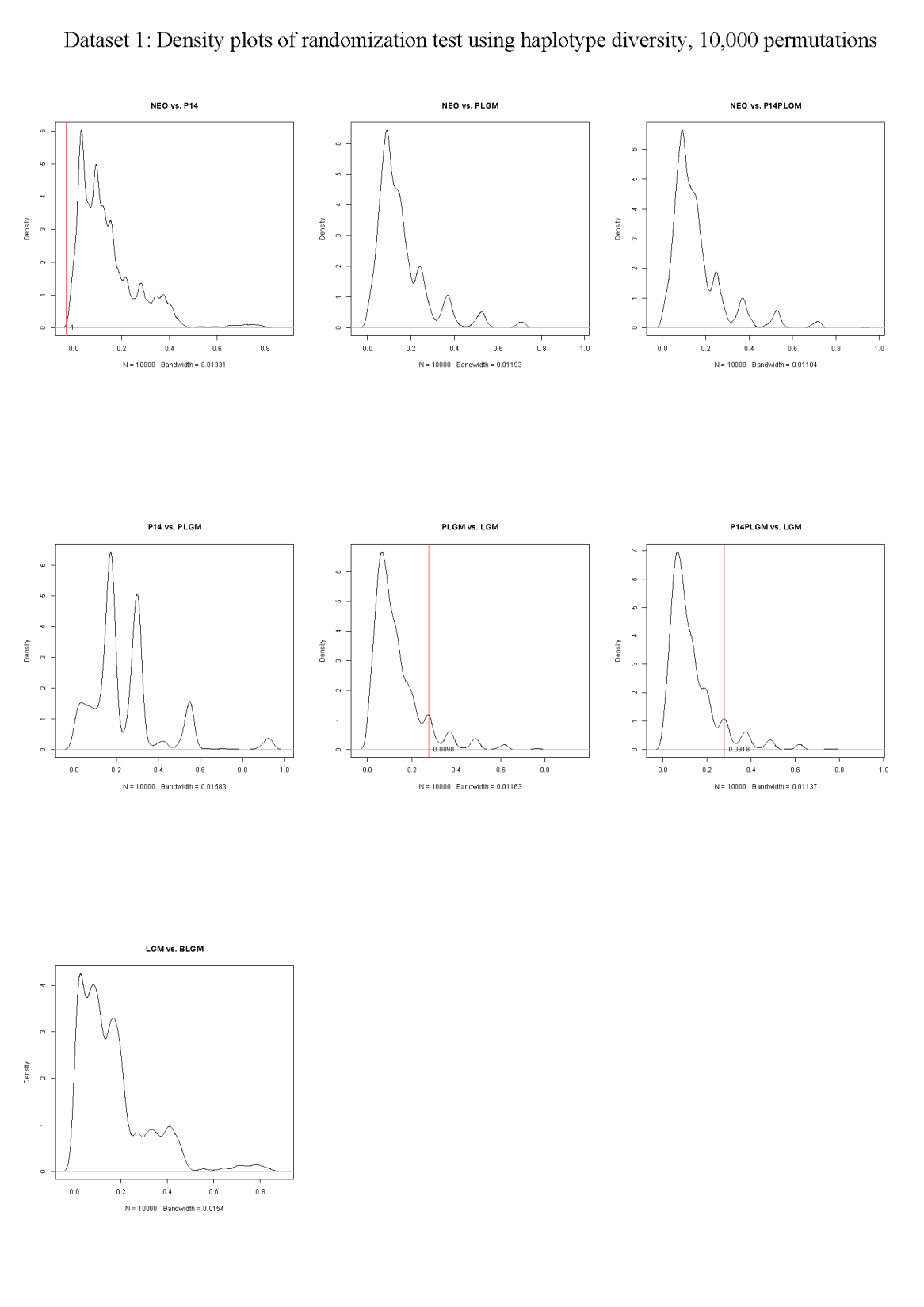


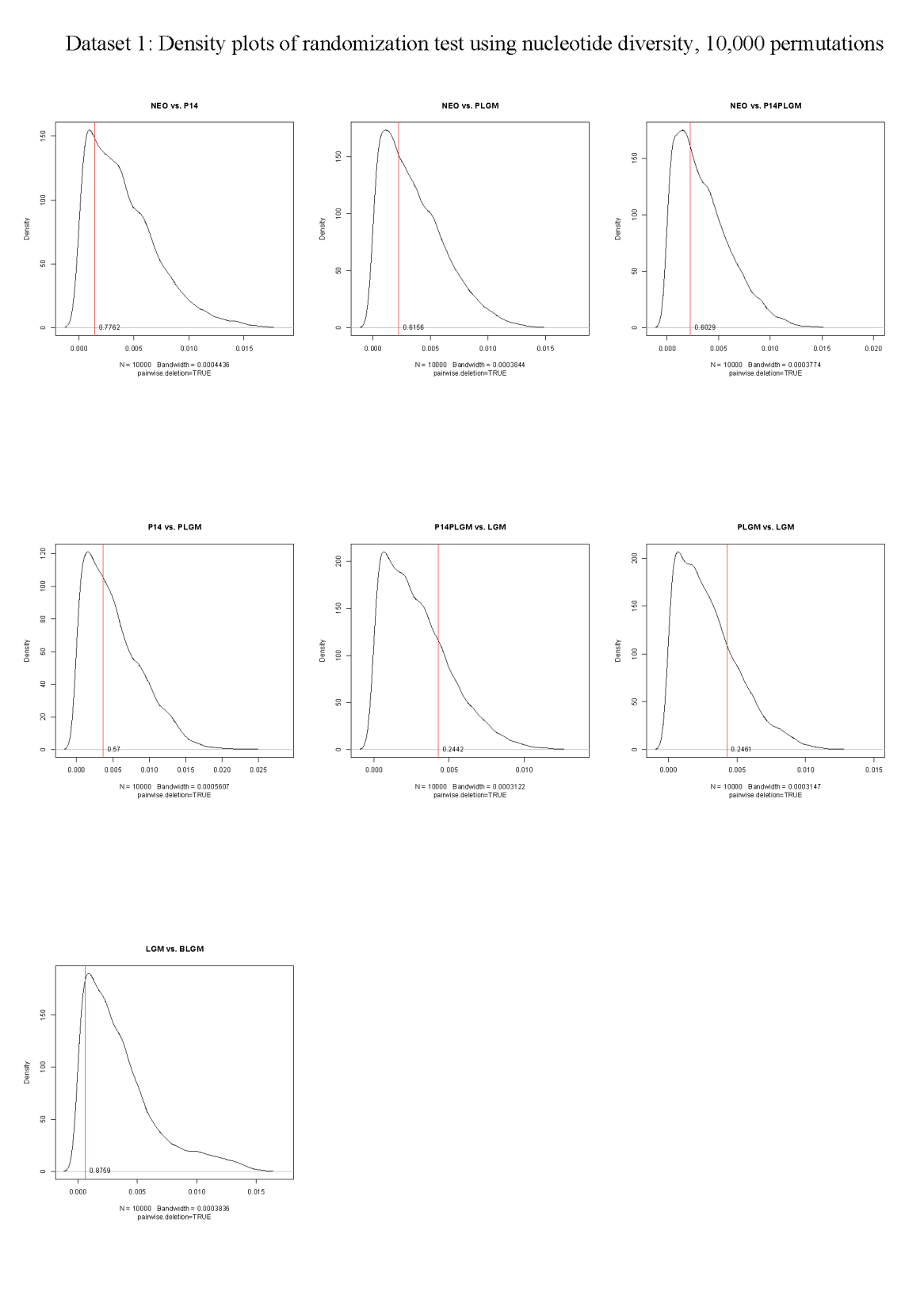

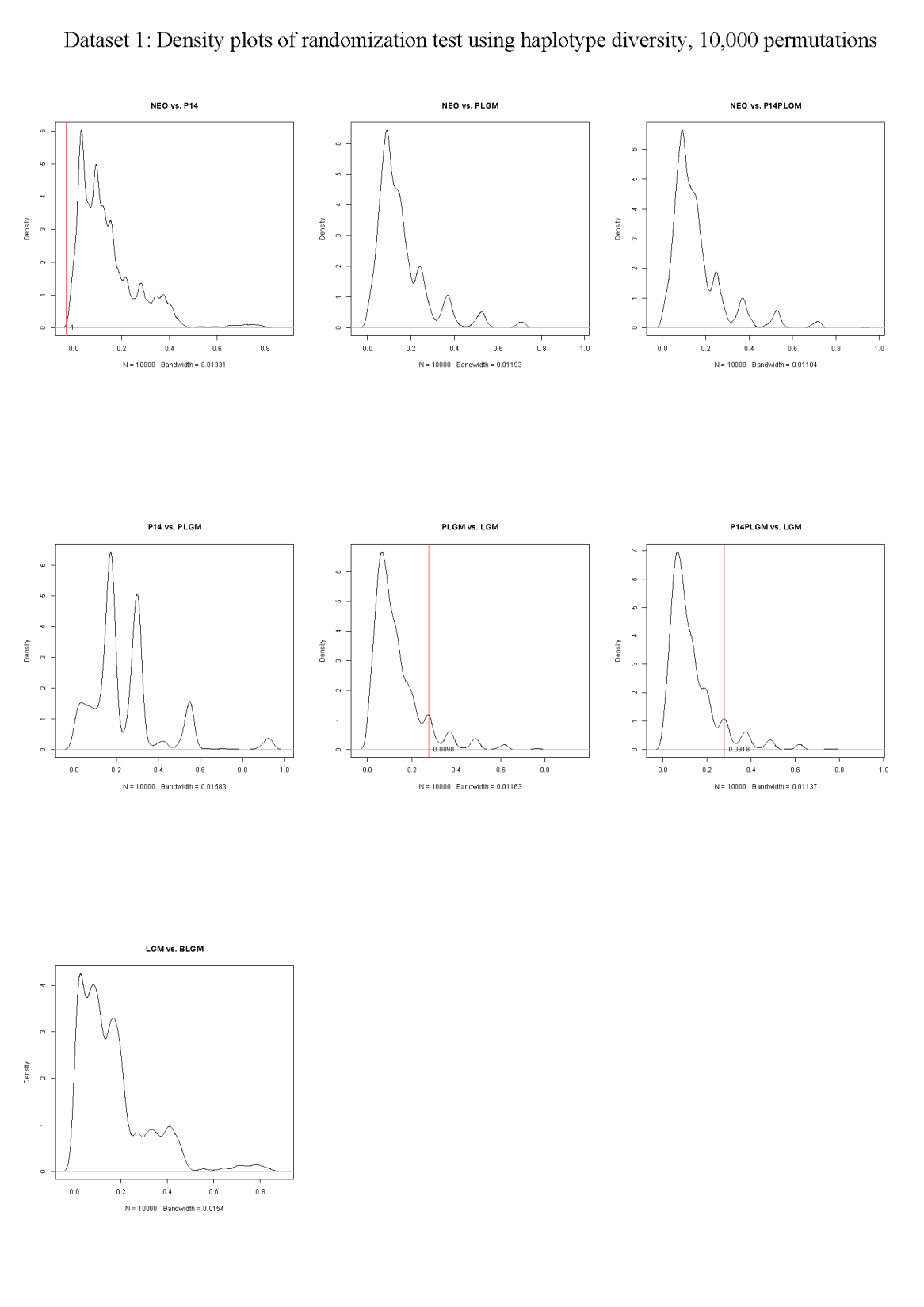


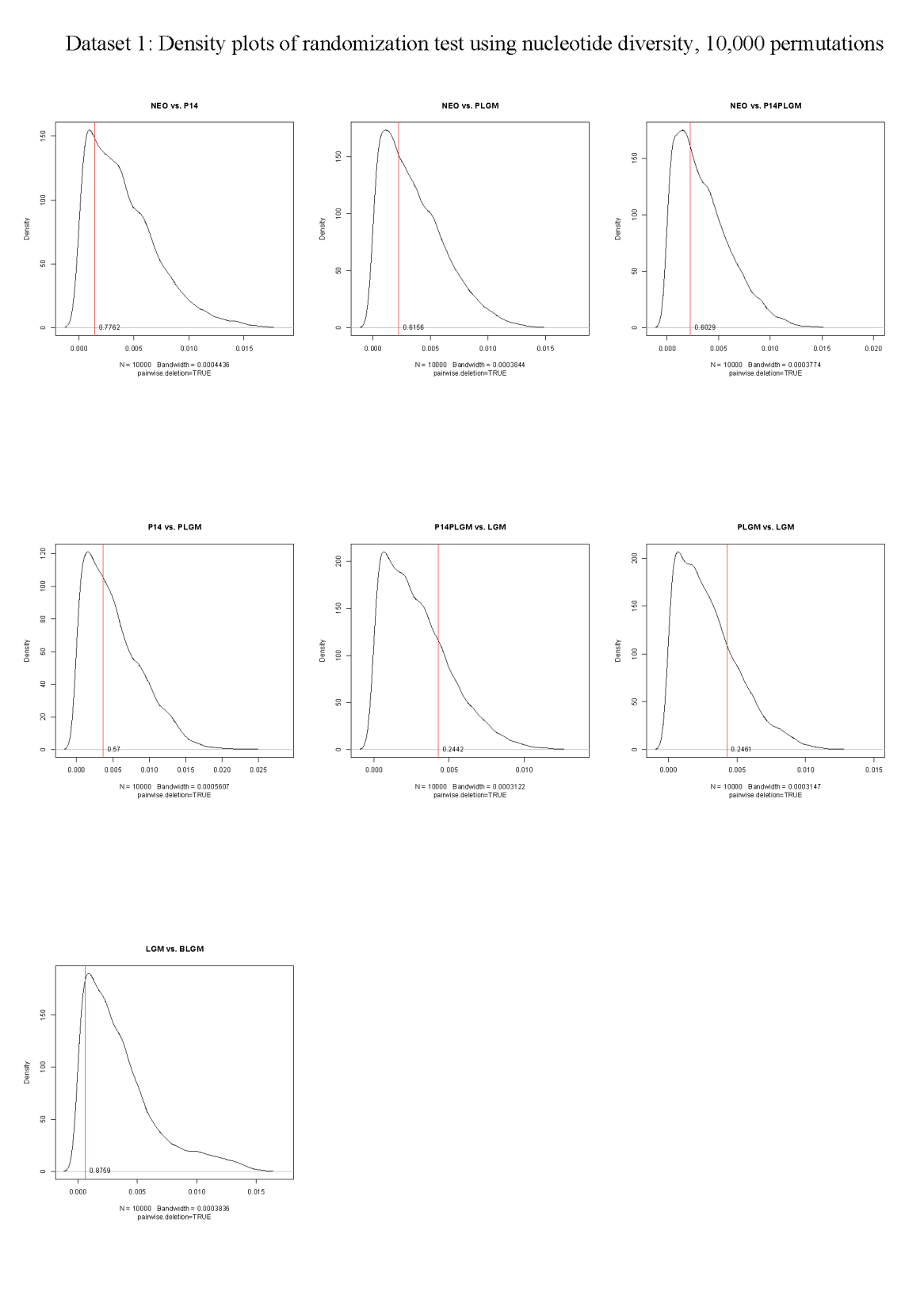

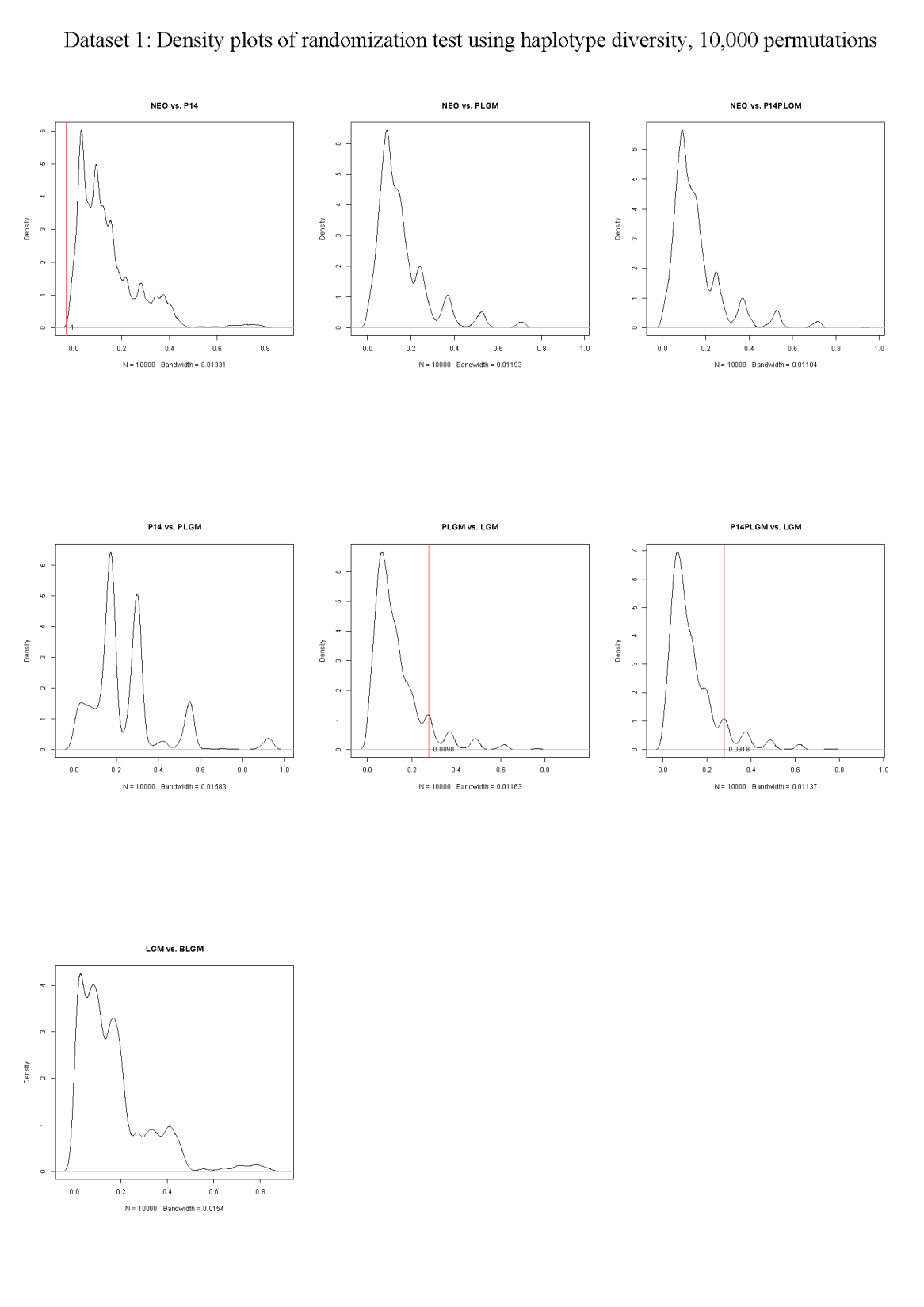


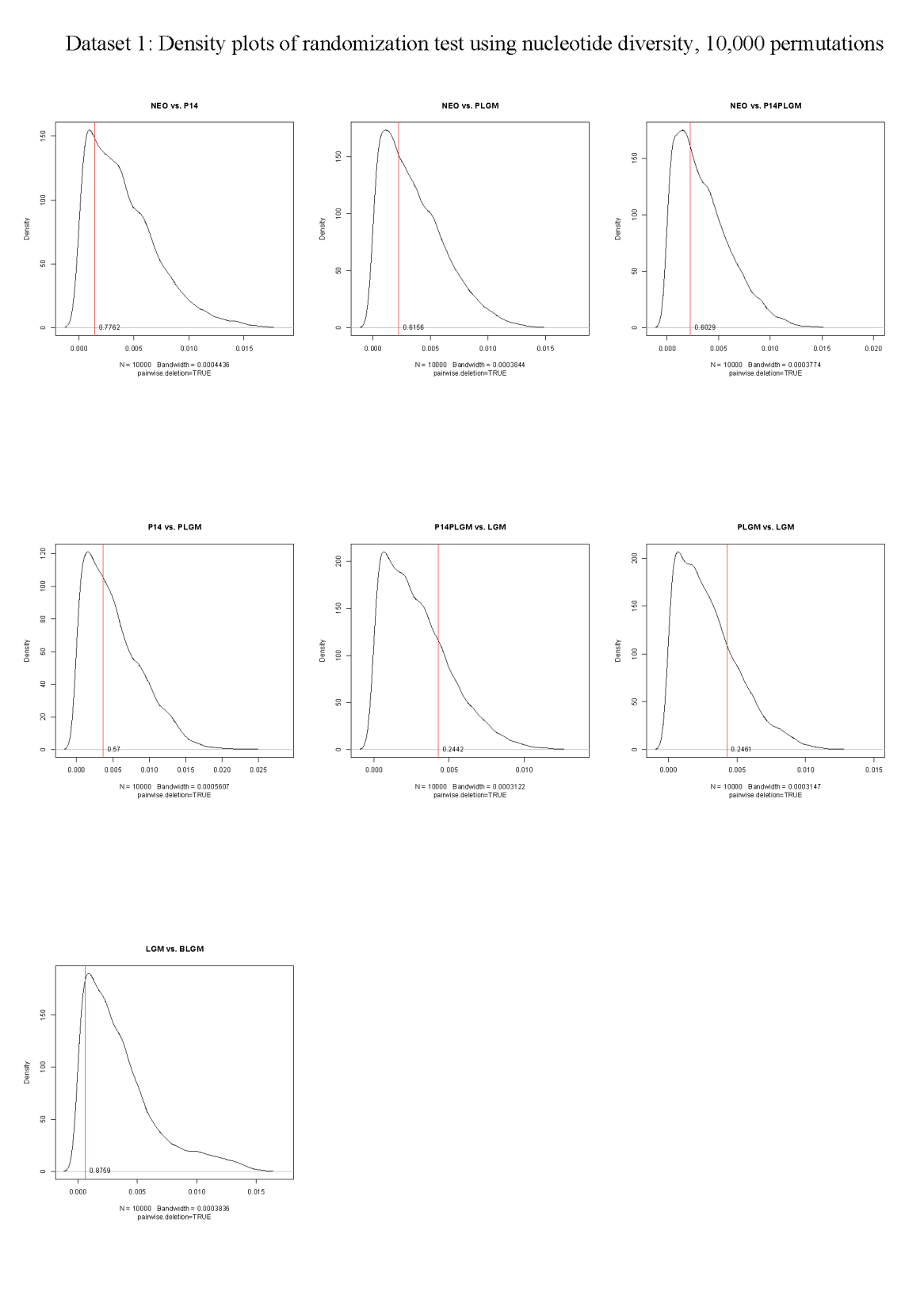

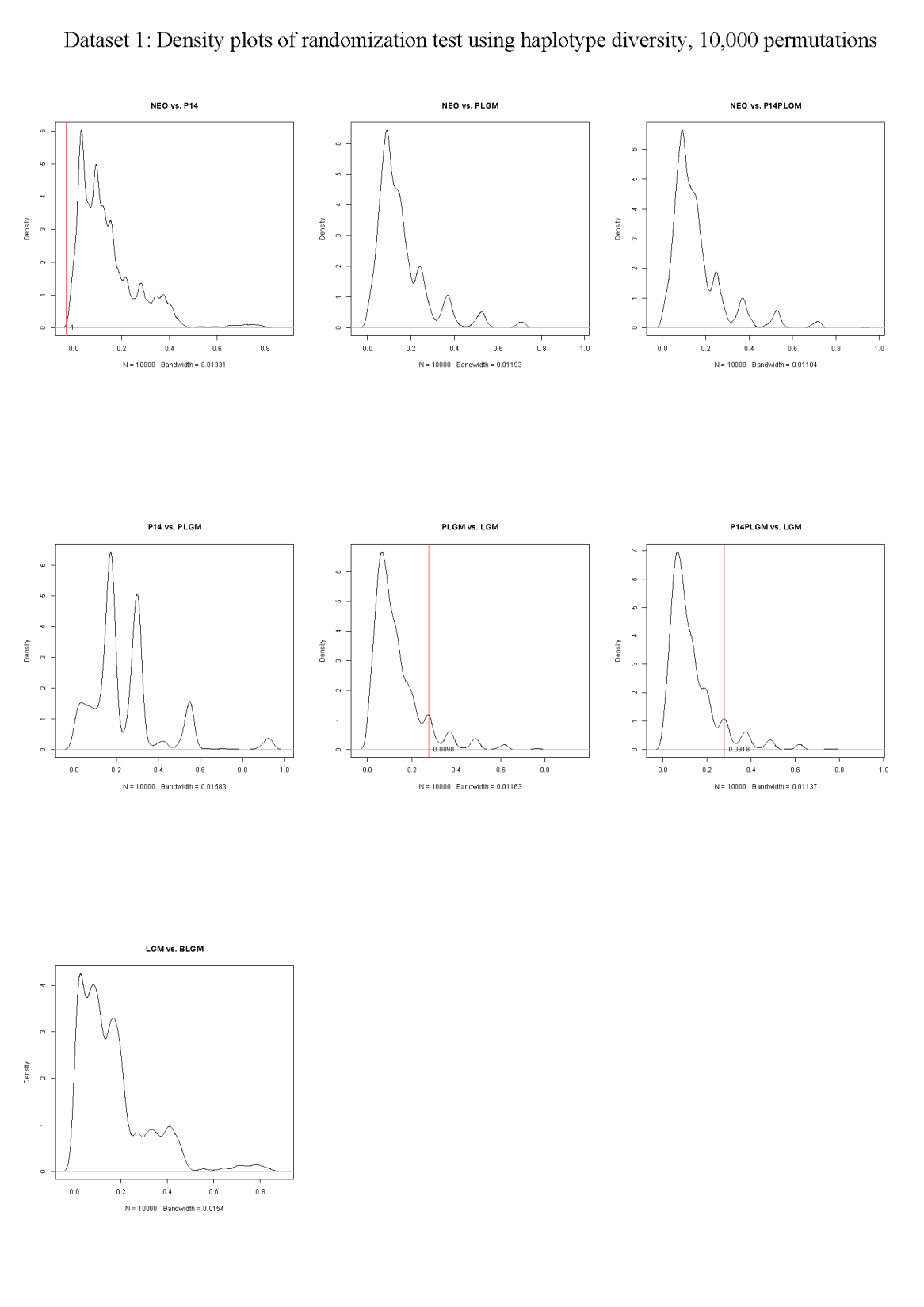


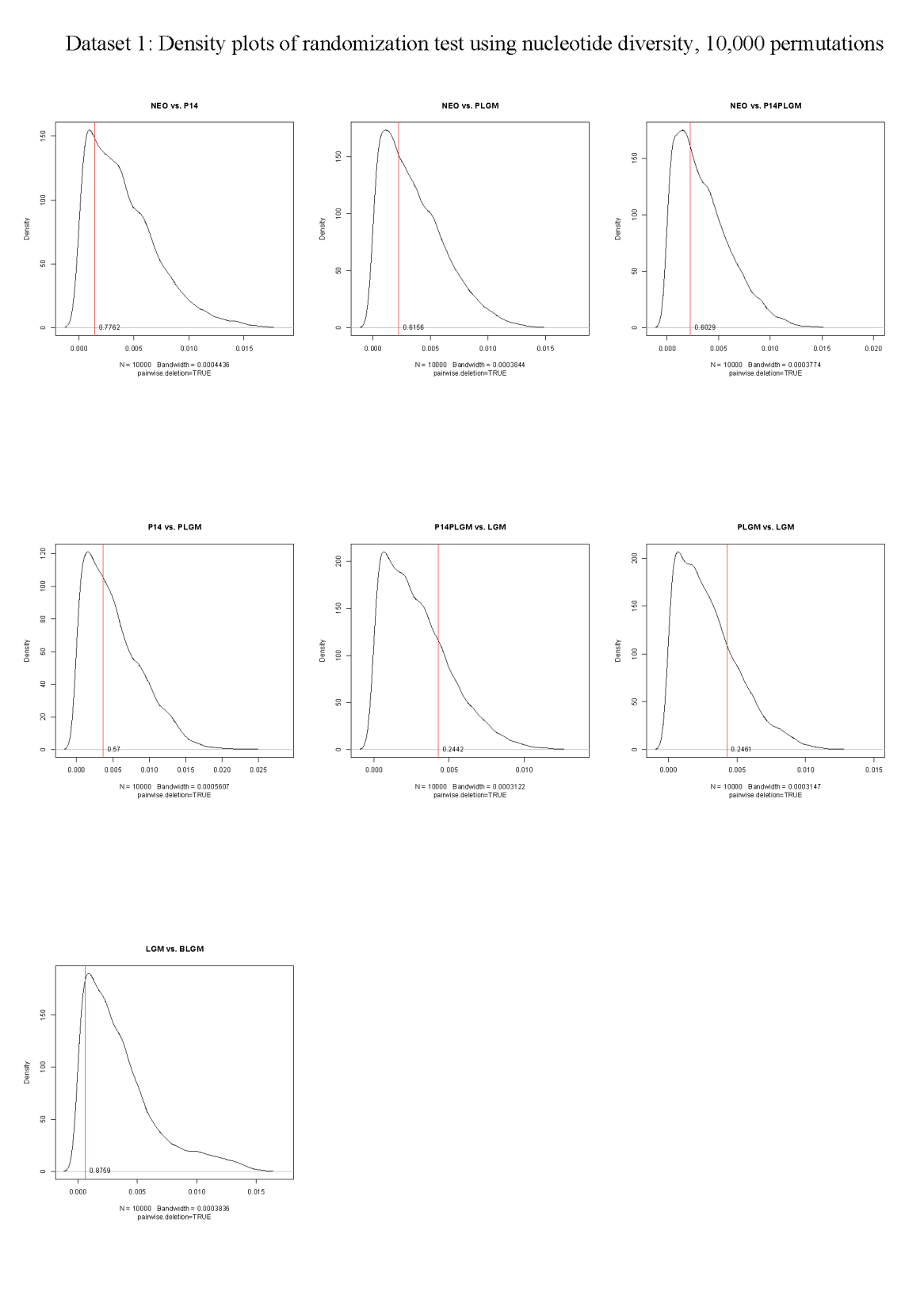

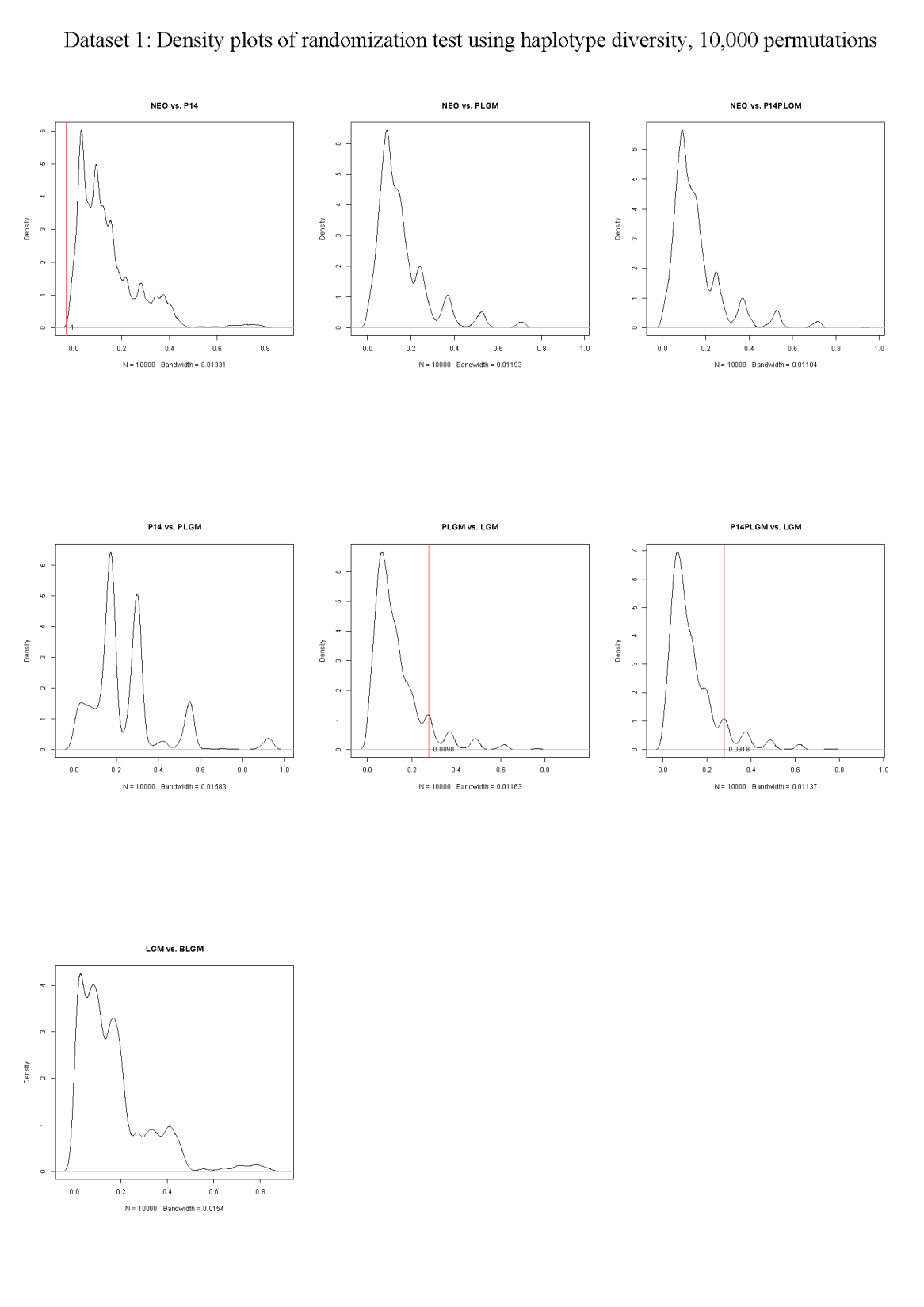


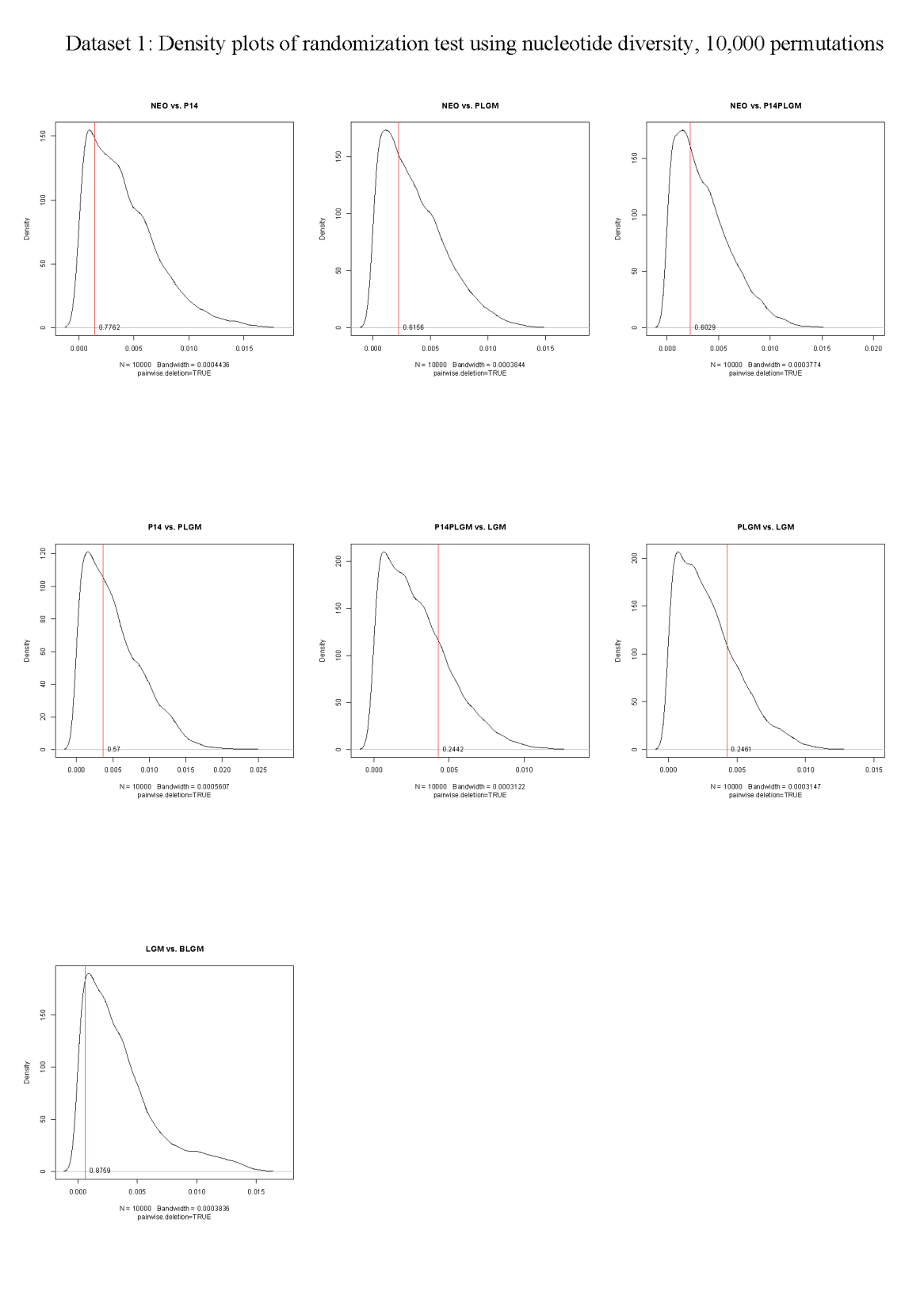

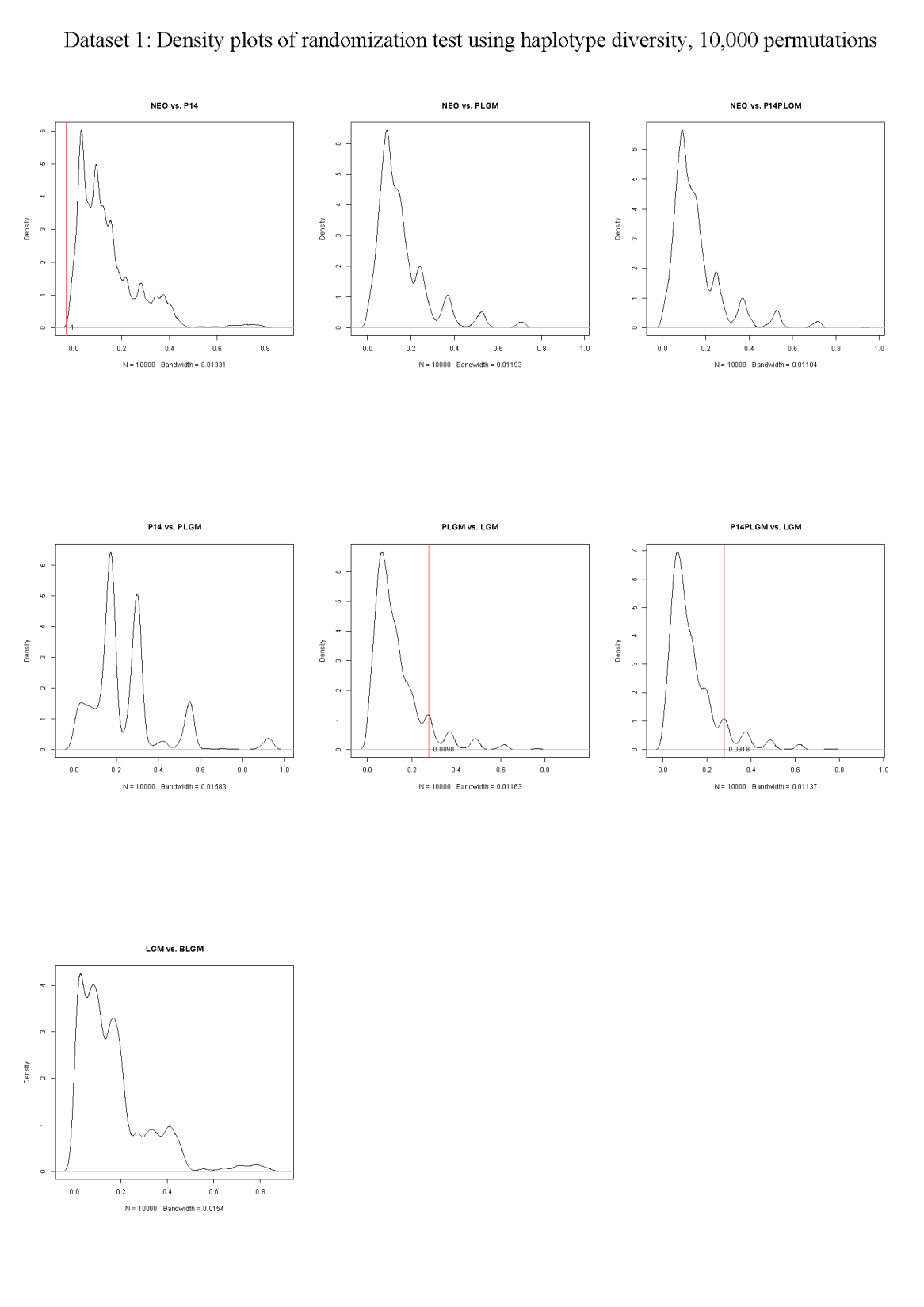


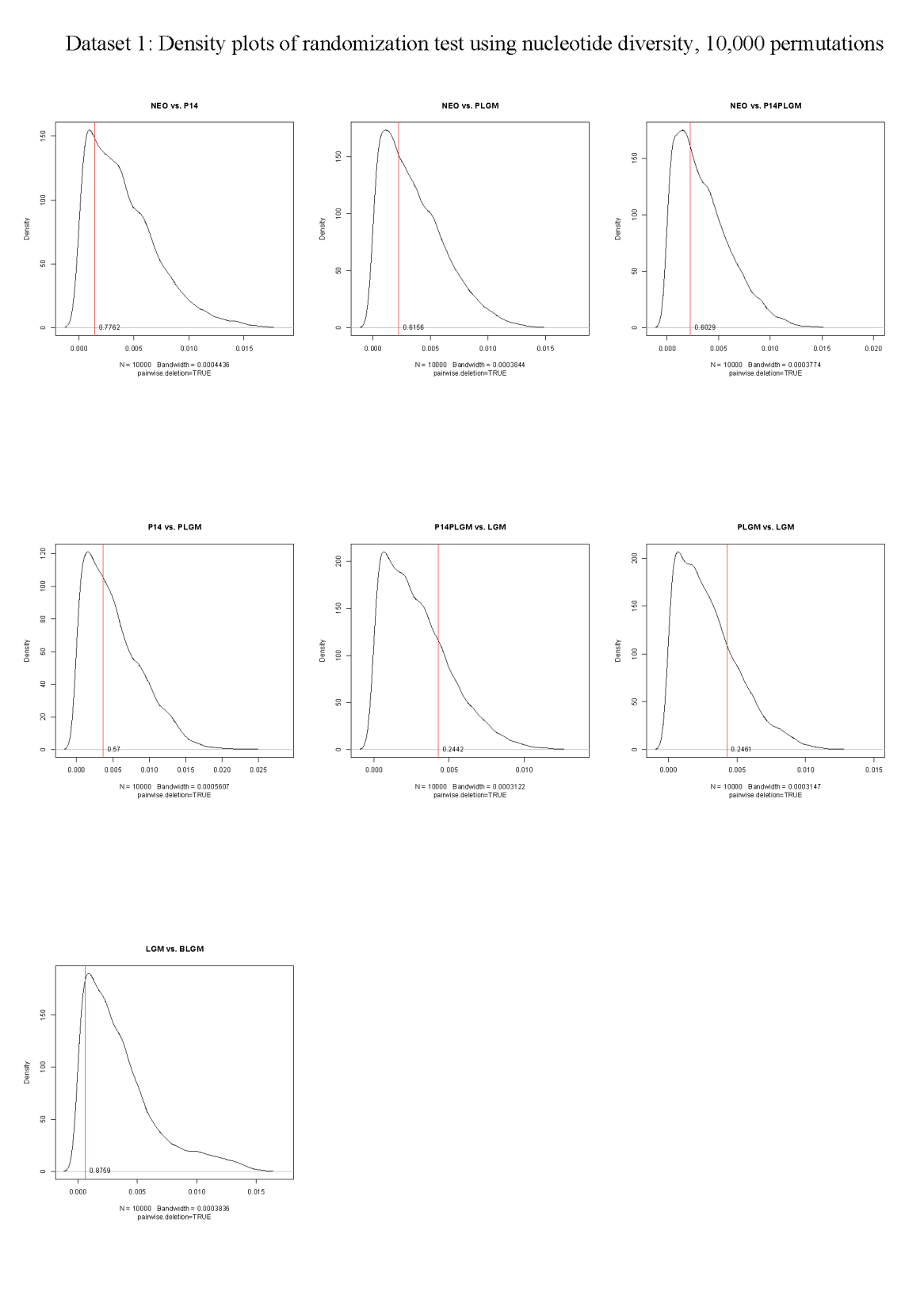

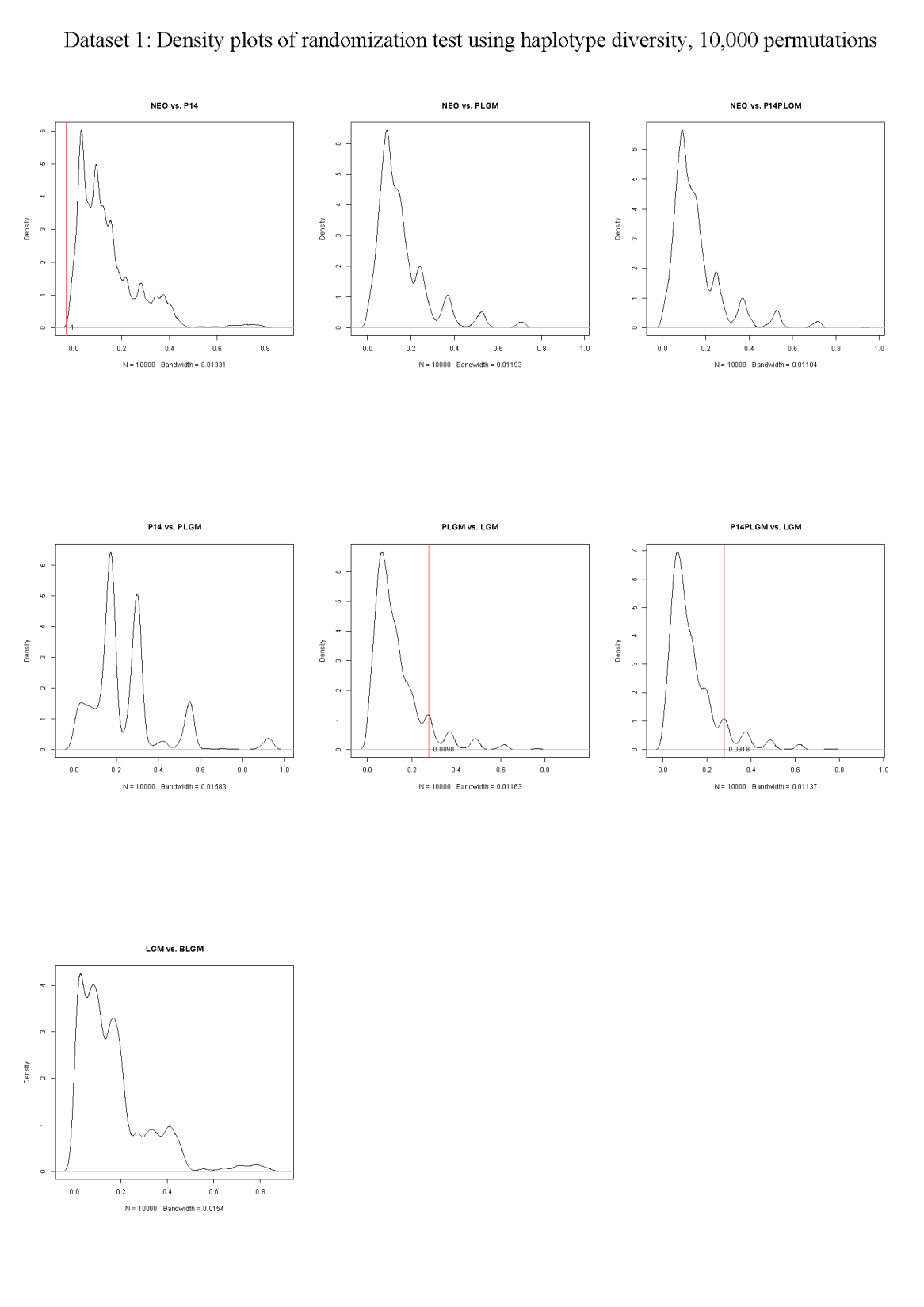


S1 Fig B: Density plots of randomisation test (10,000 permutations with replacement) based on nucleotide (left panel) and haplotype (right panel) diversity. Unbiased and thus comparable pairs are framed blue. Dataset 2.


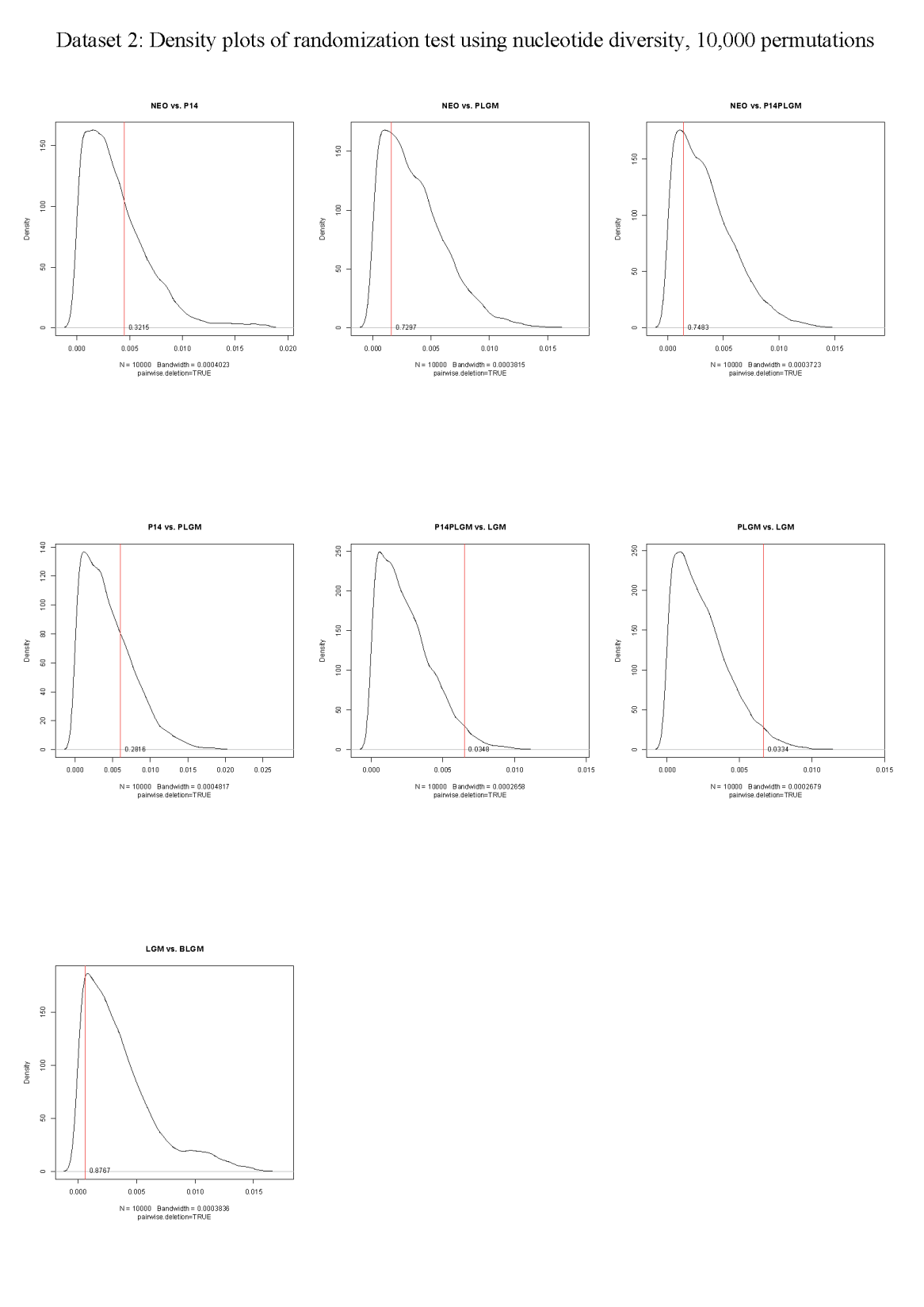

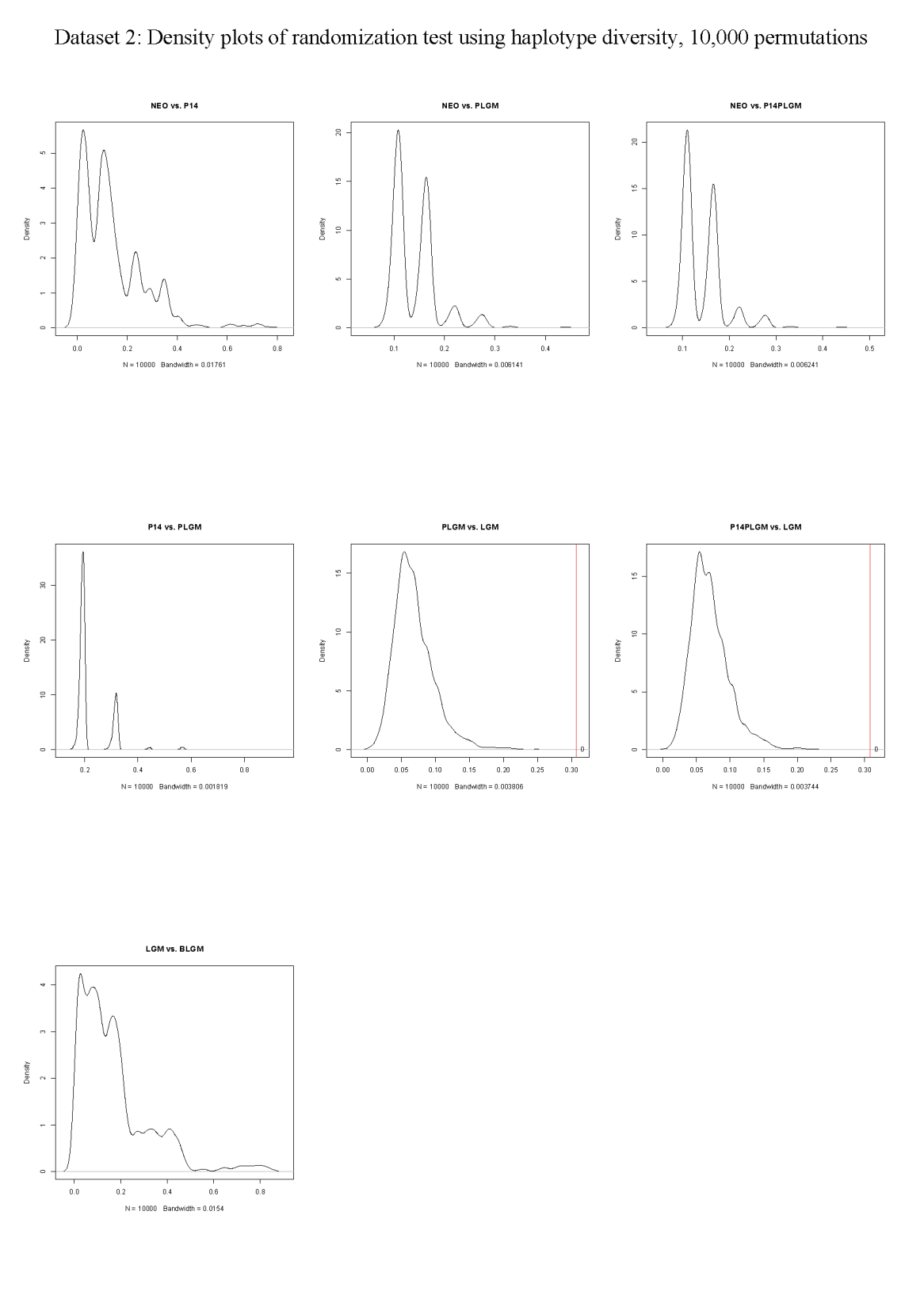


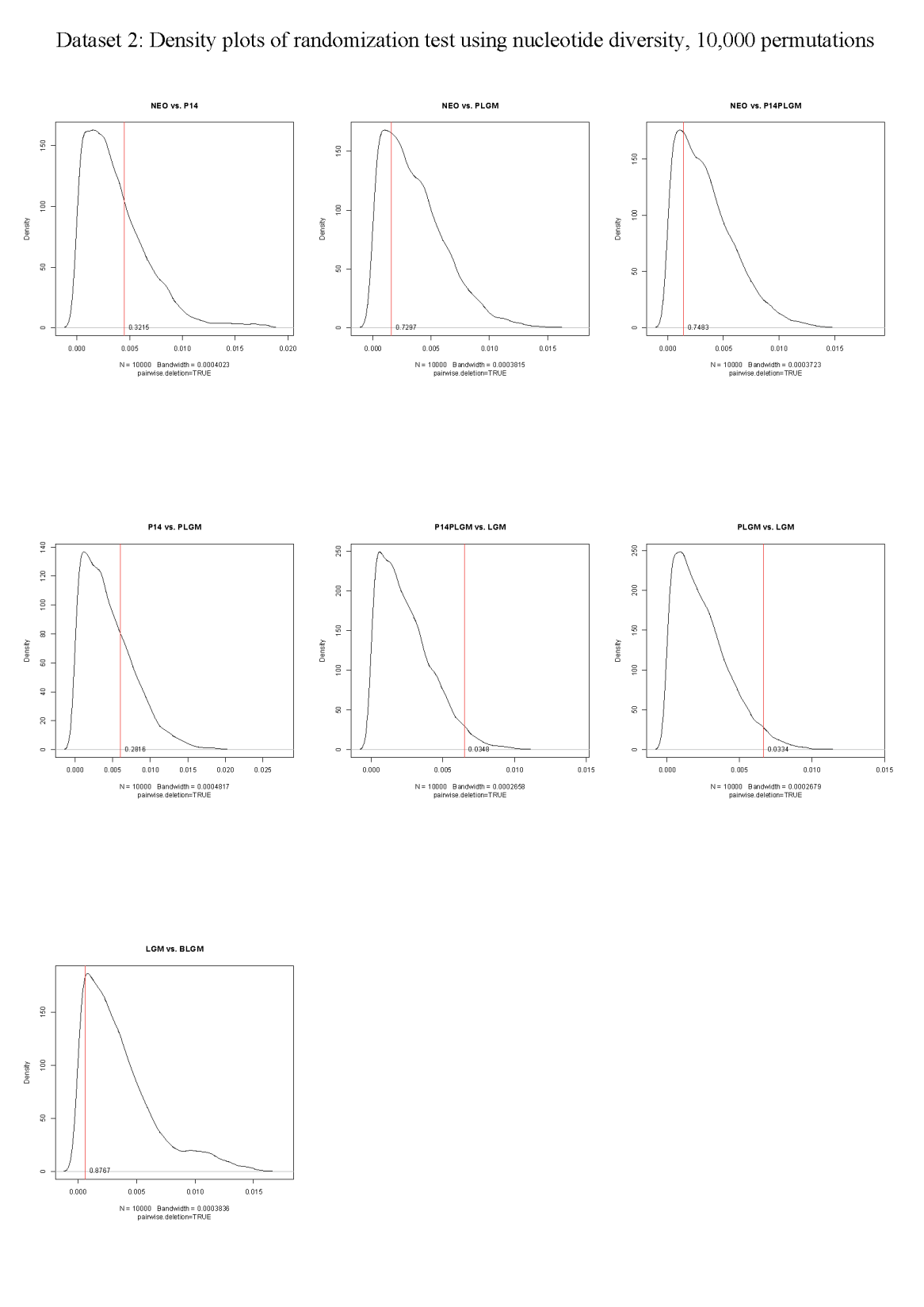

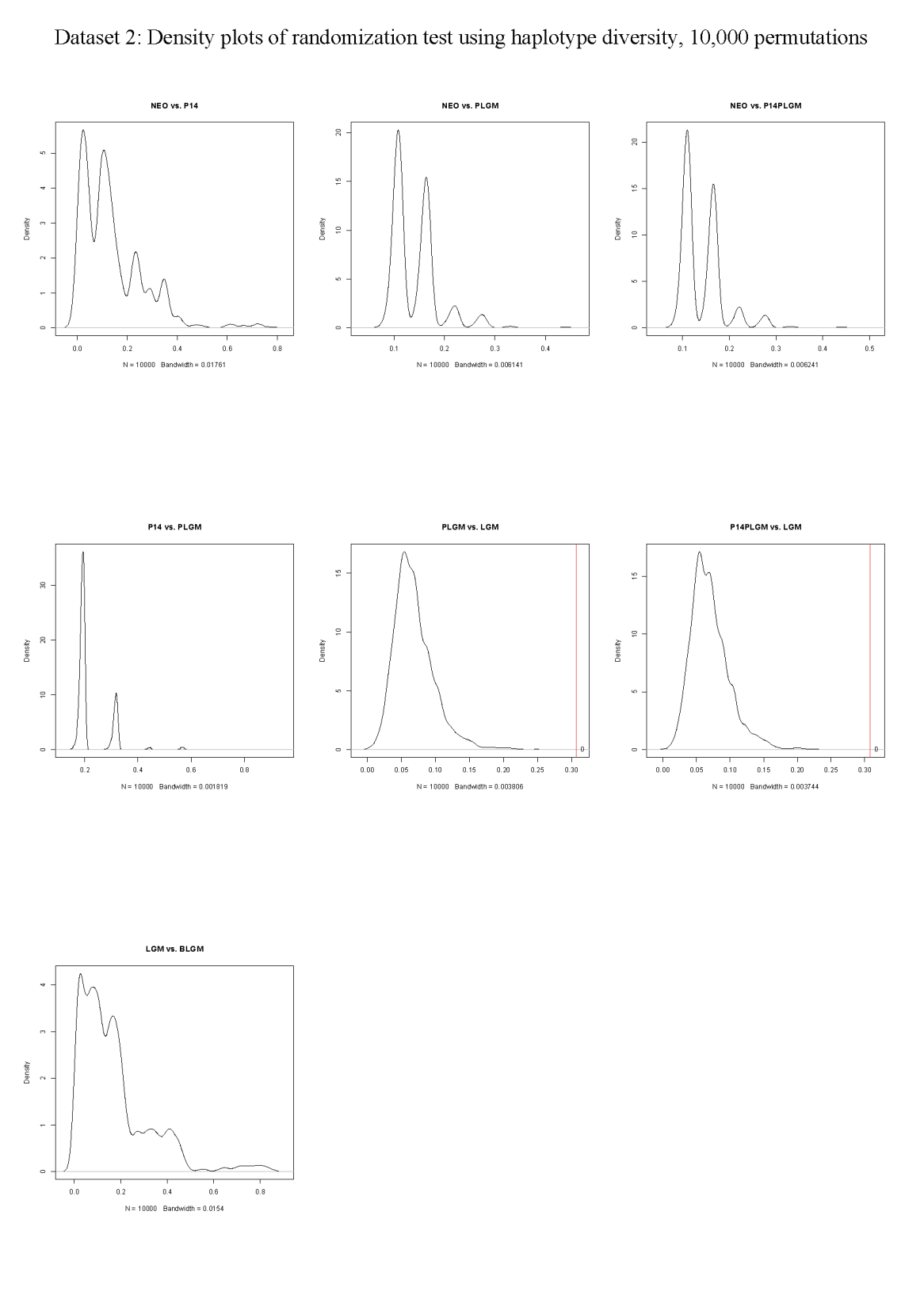


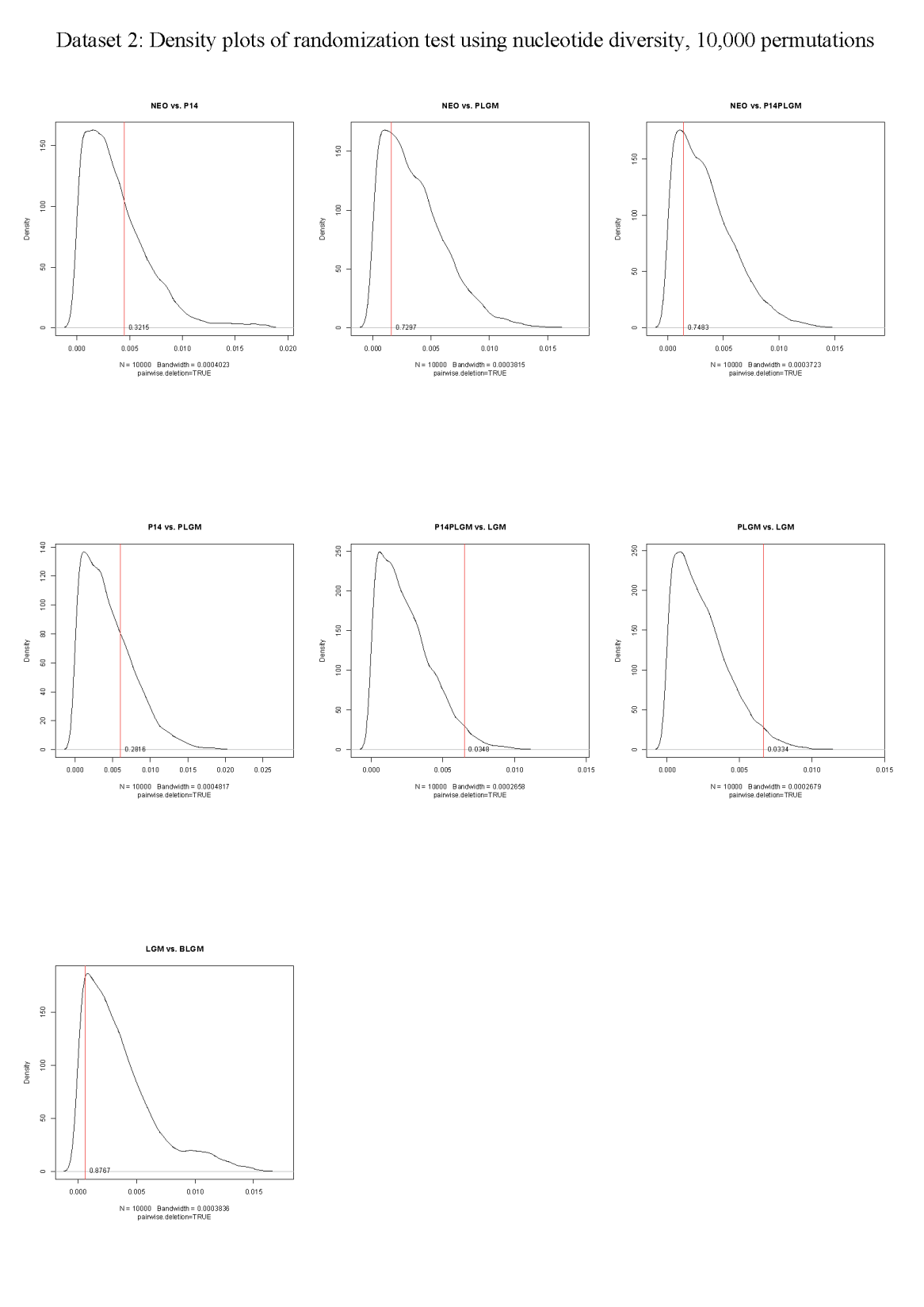

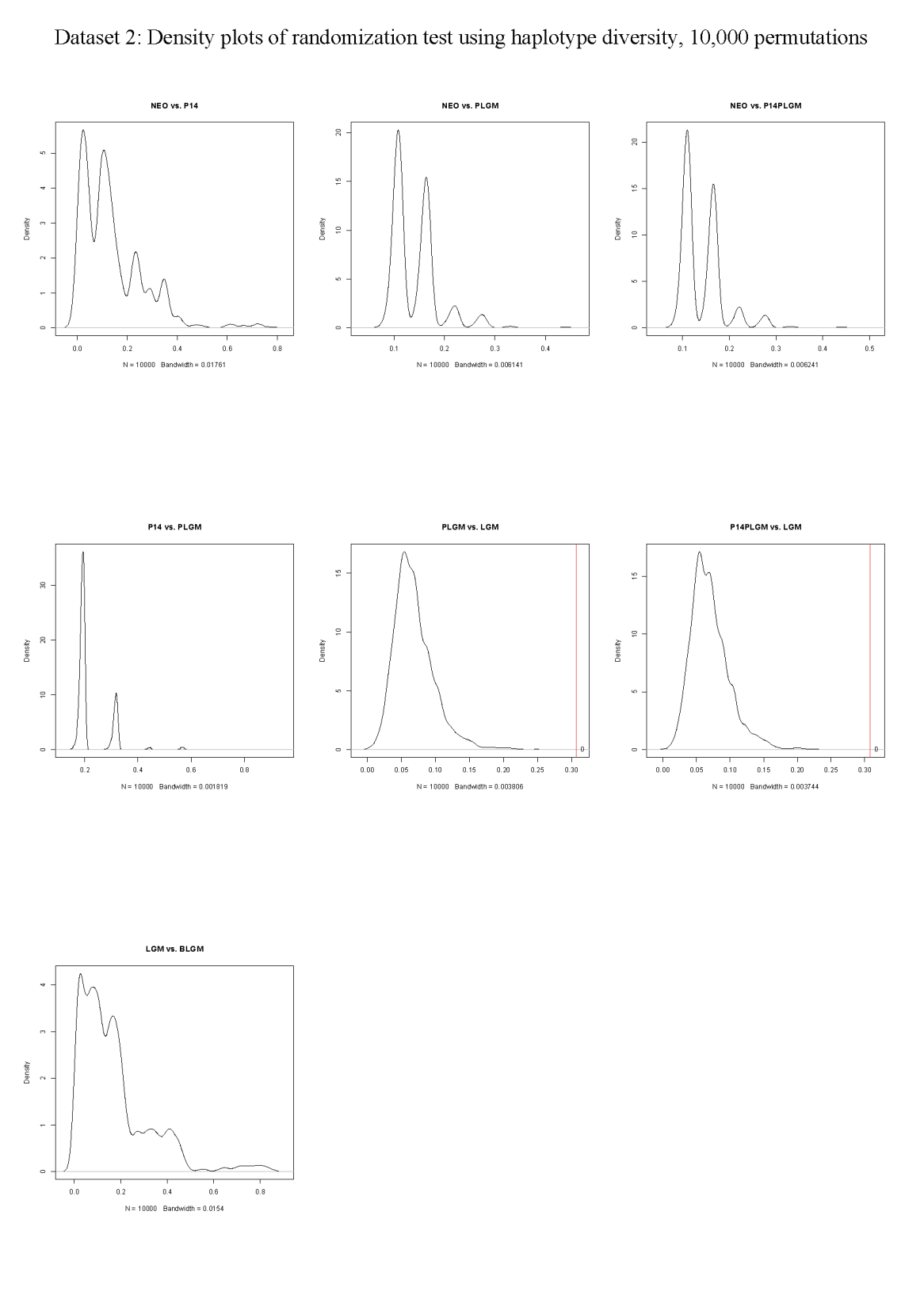


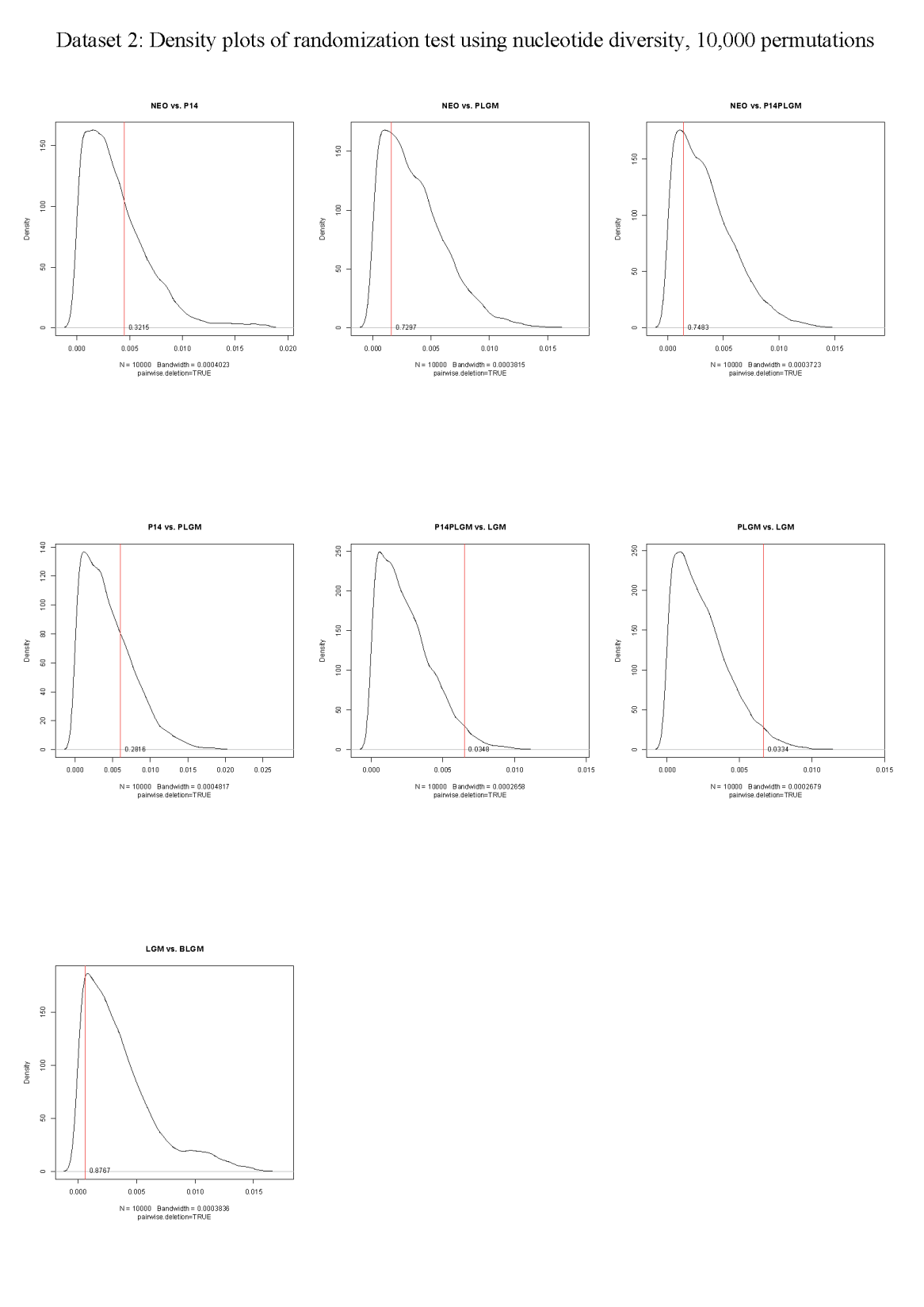

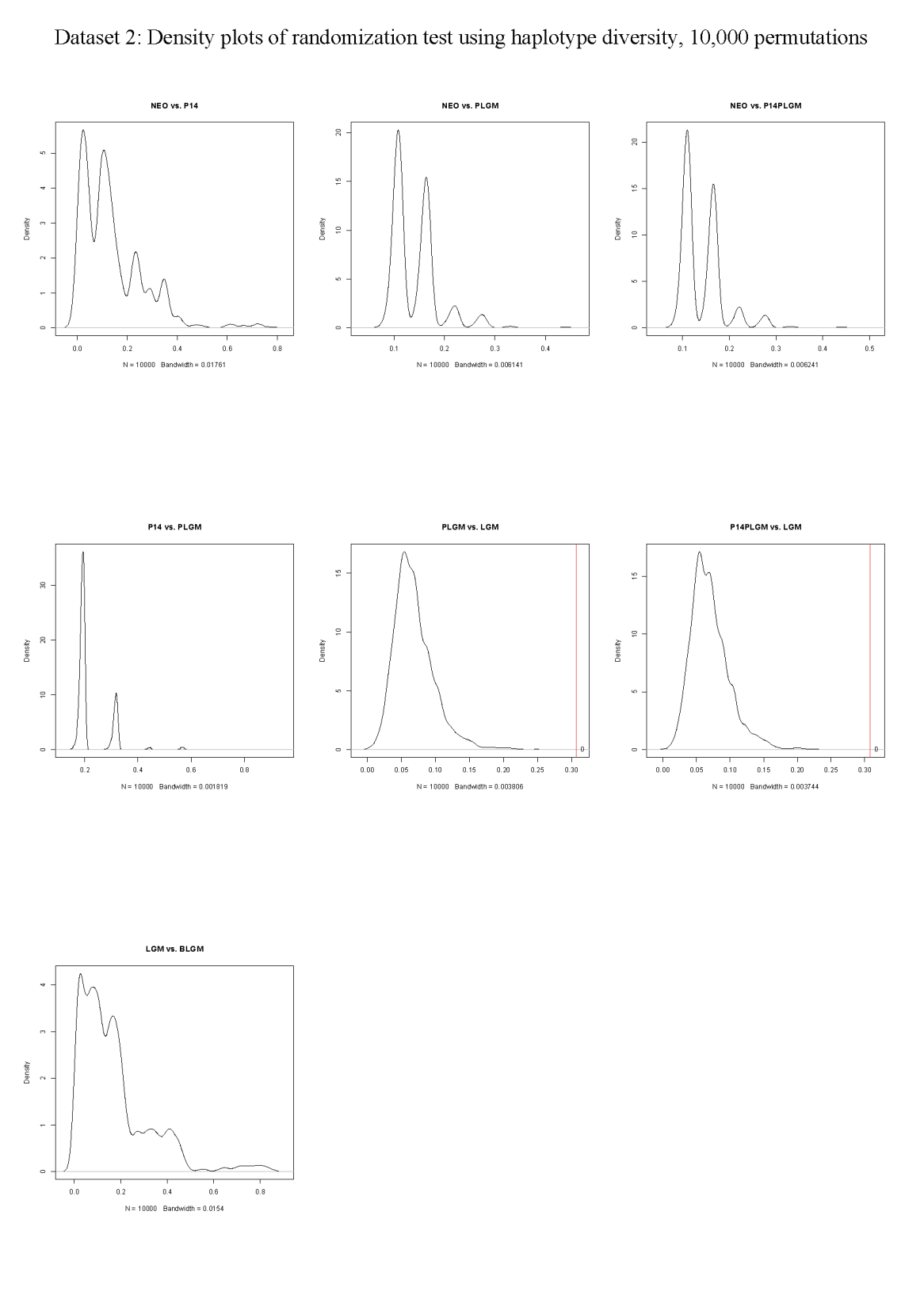


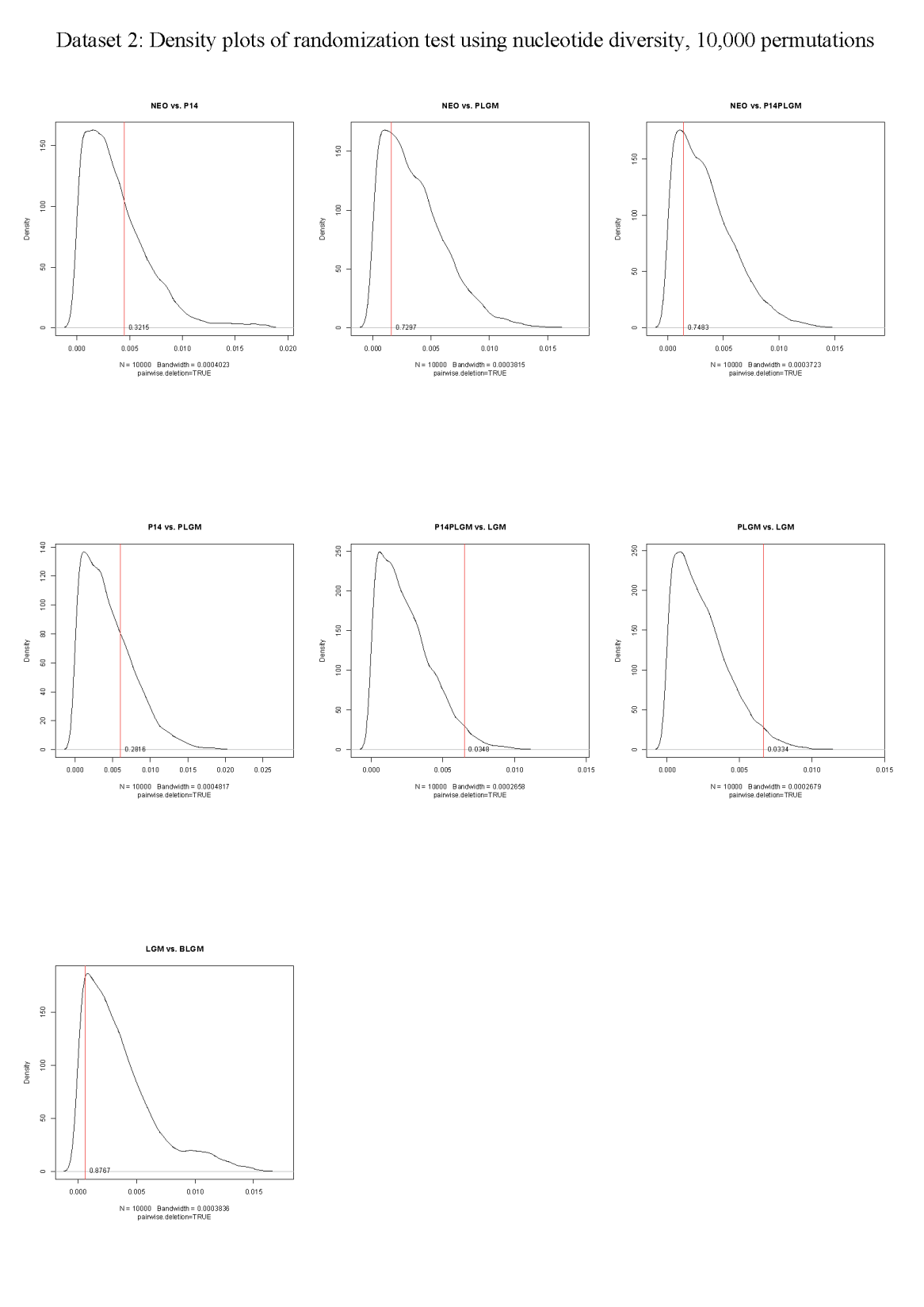

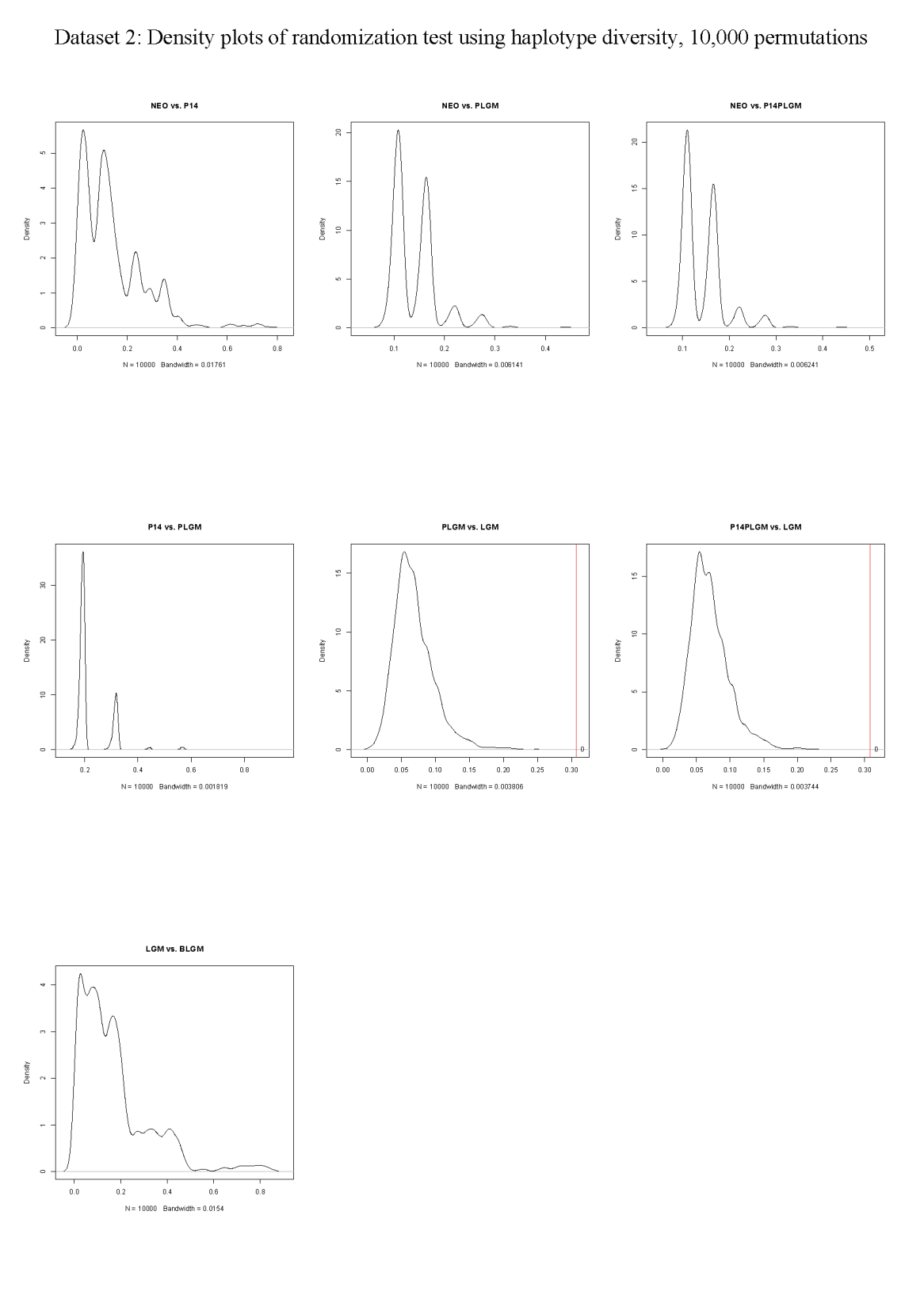


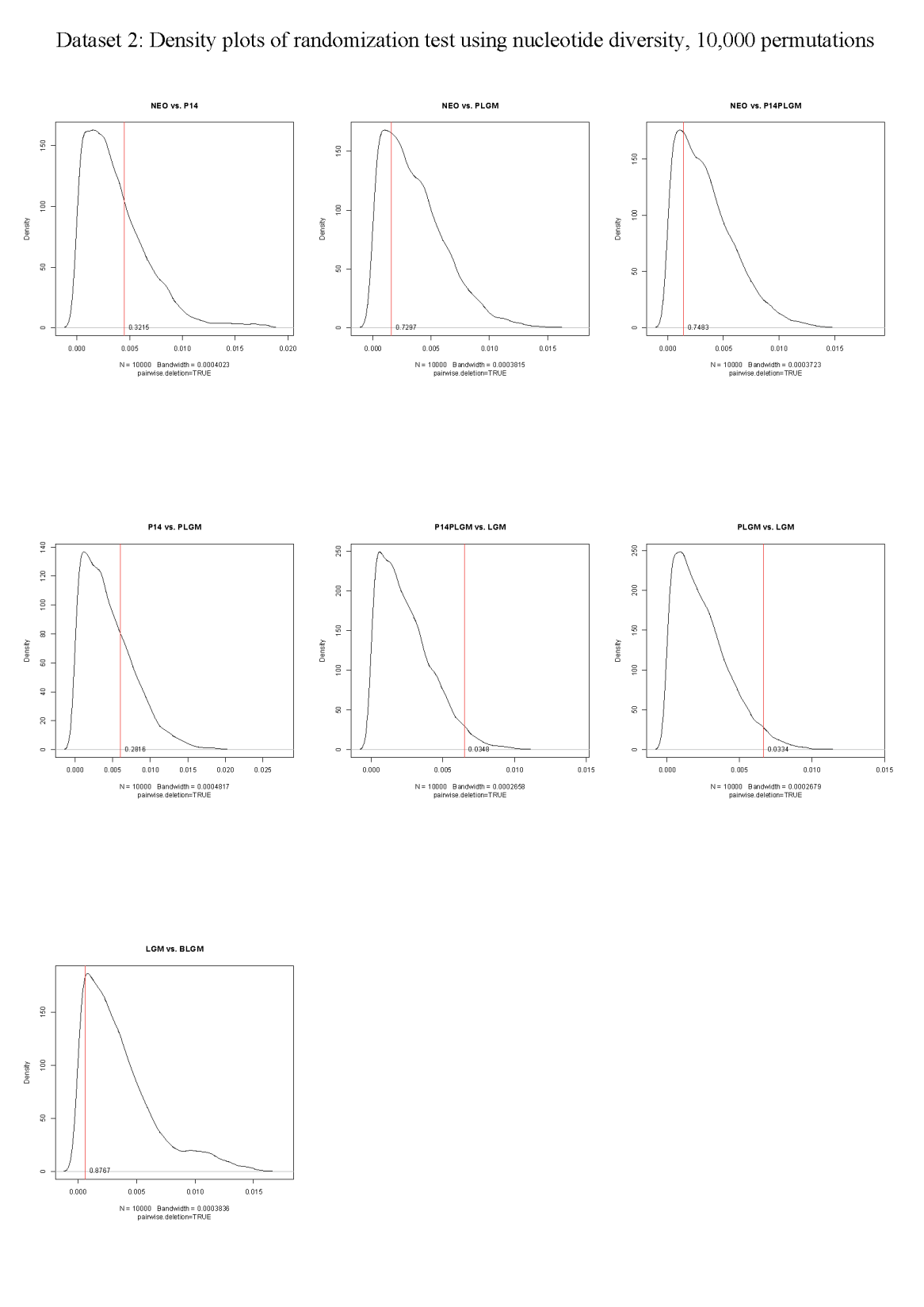

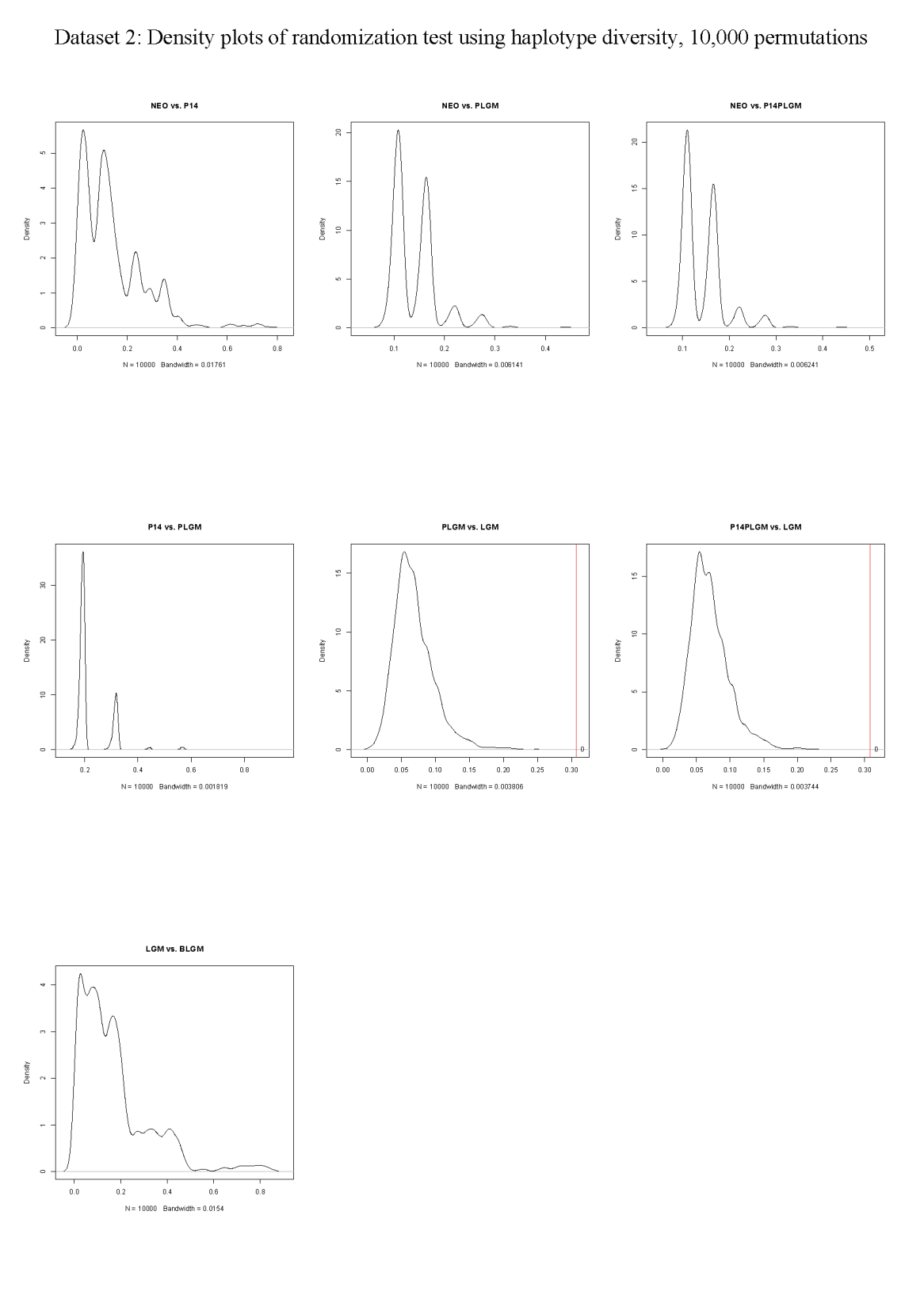


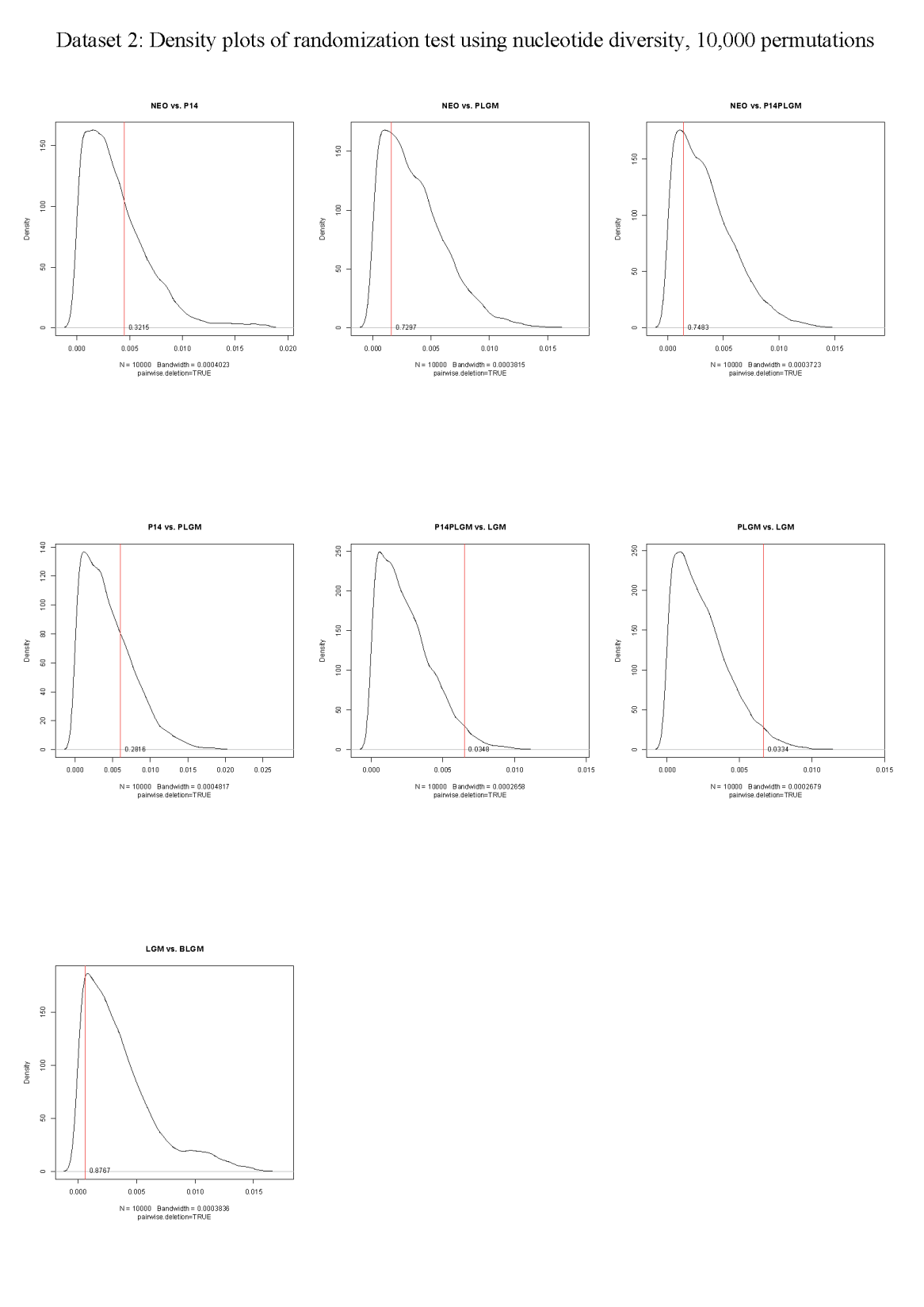

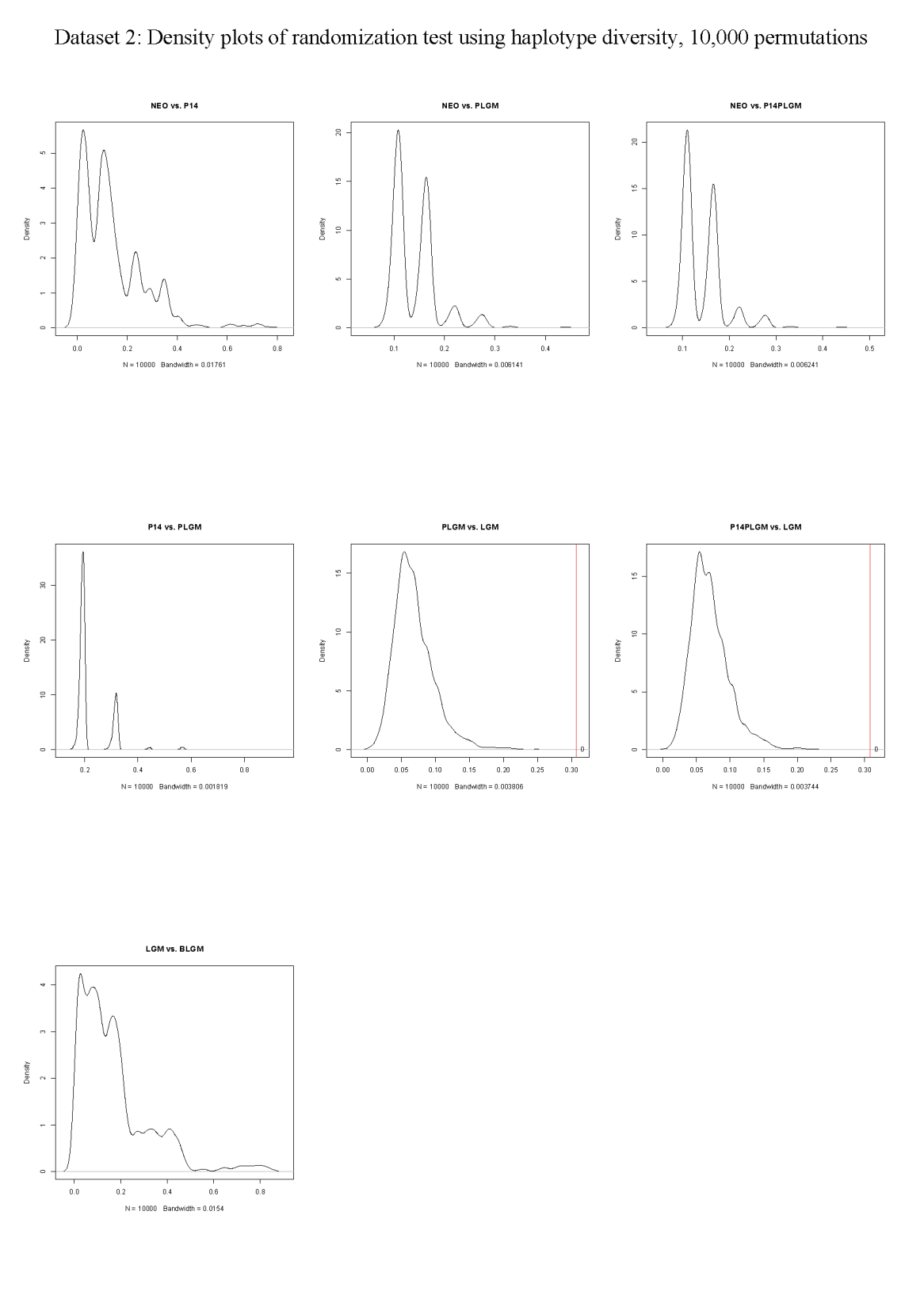


S1 Fig C: Density plots of randomisation test (10,000 permutations with replacement) based on nucleotide (left panel) and haplotype (right panel) diversity. Unbiased and thus comparable pairs are framed blue. Dataset 3.


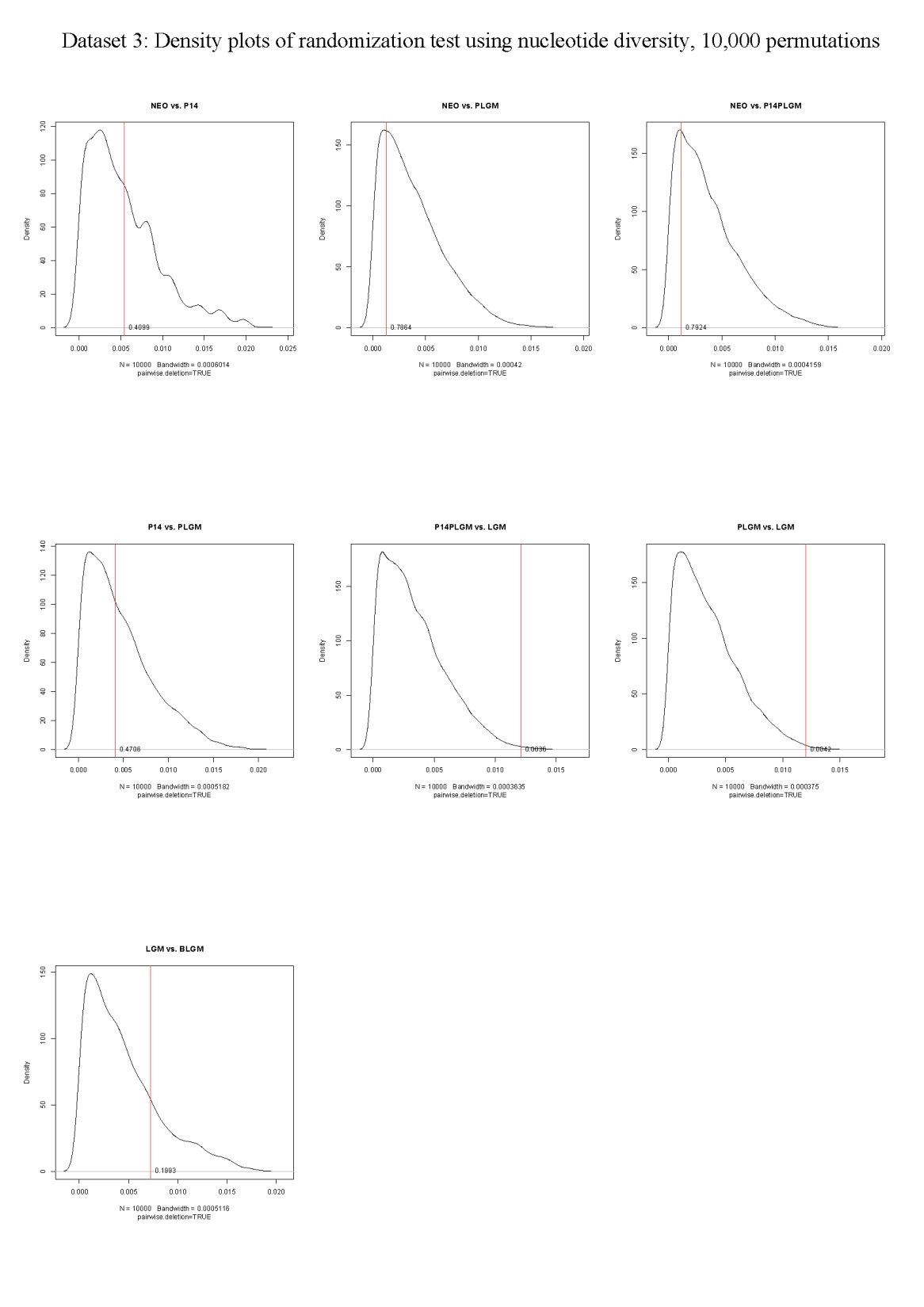

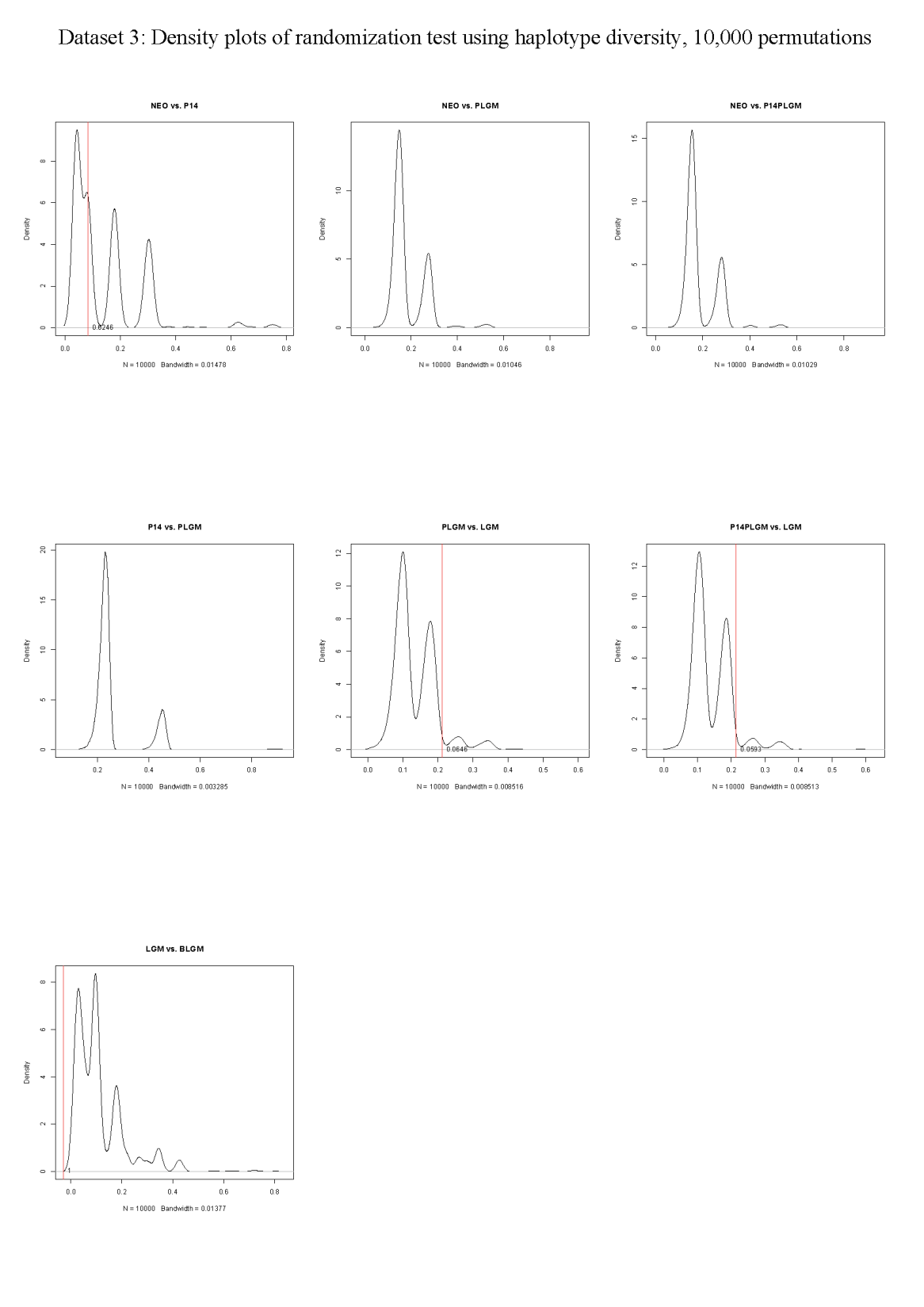


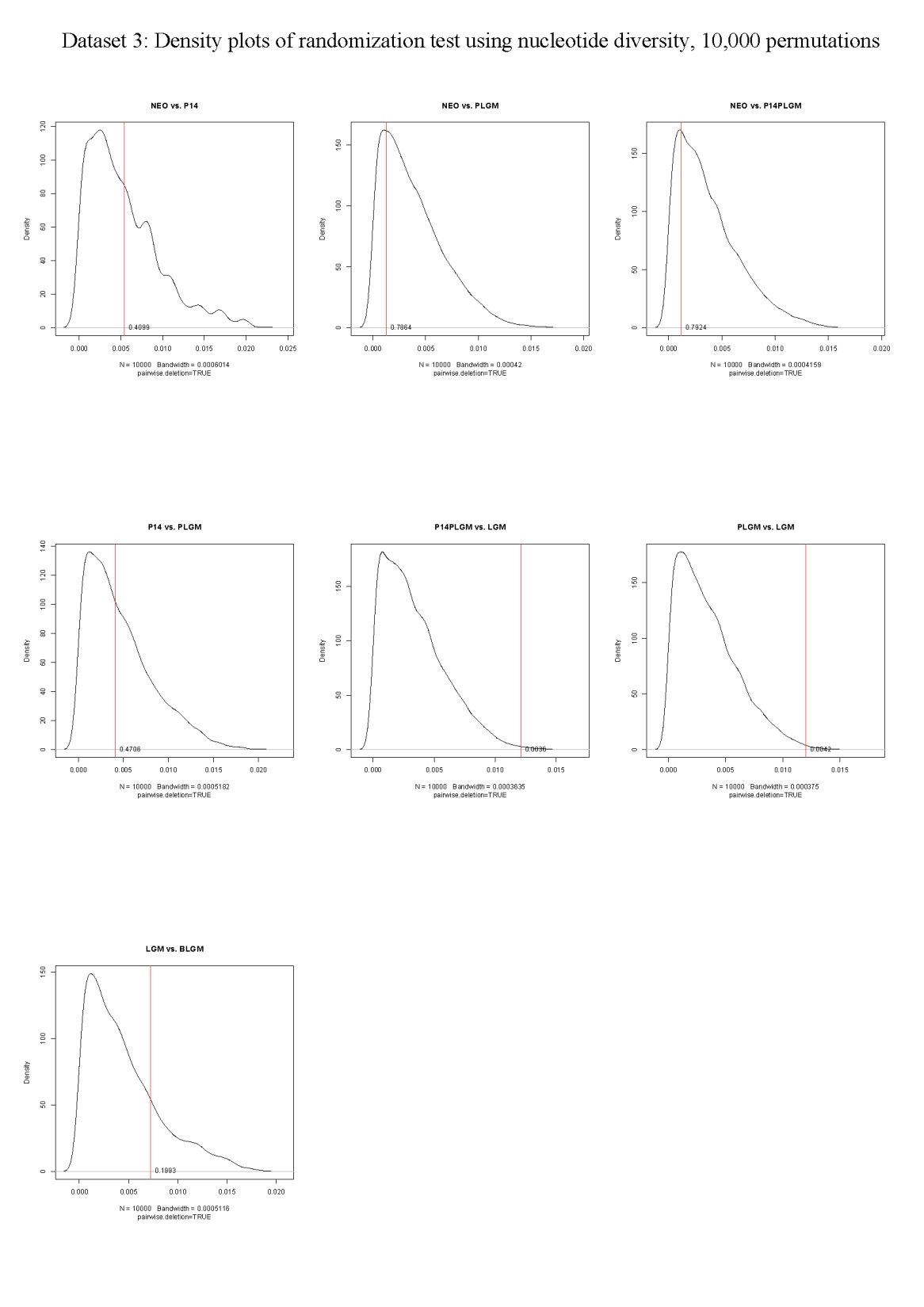

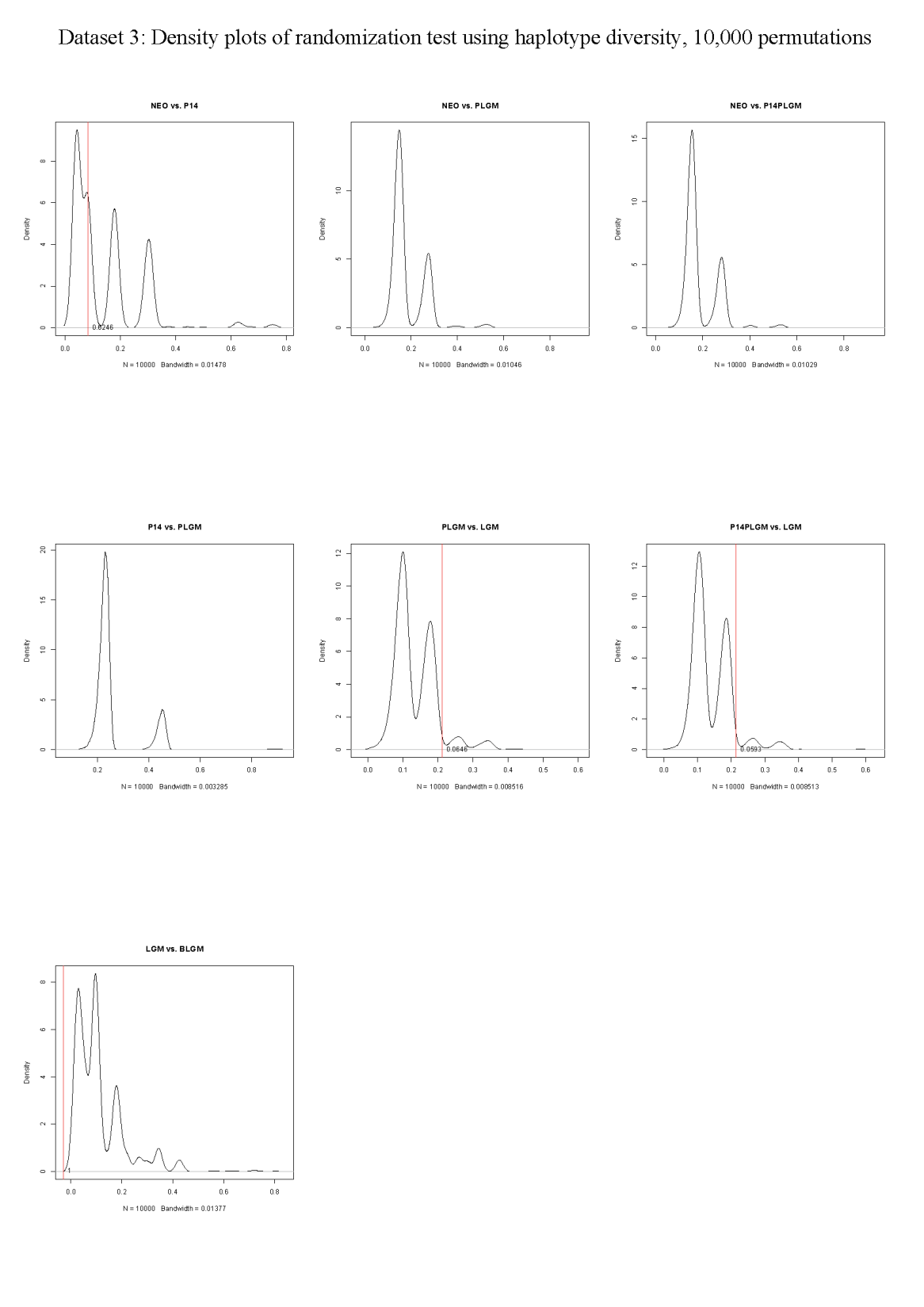


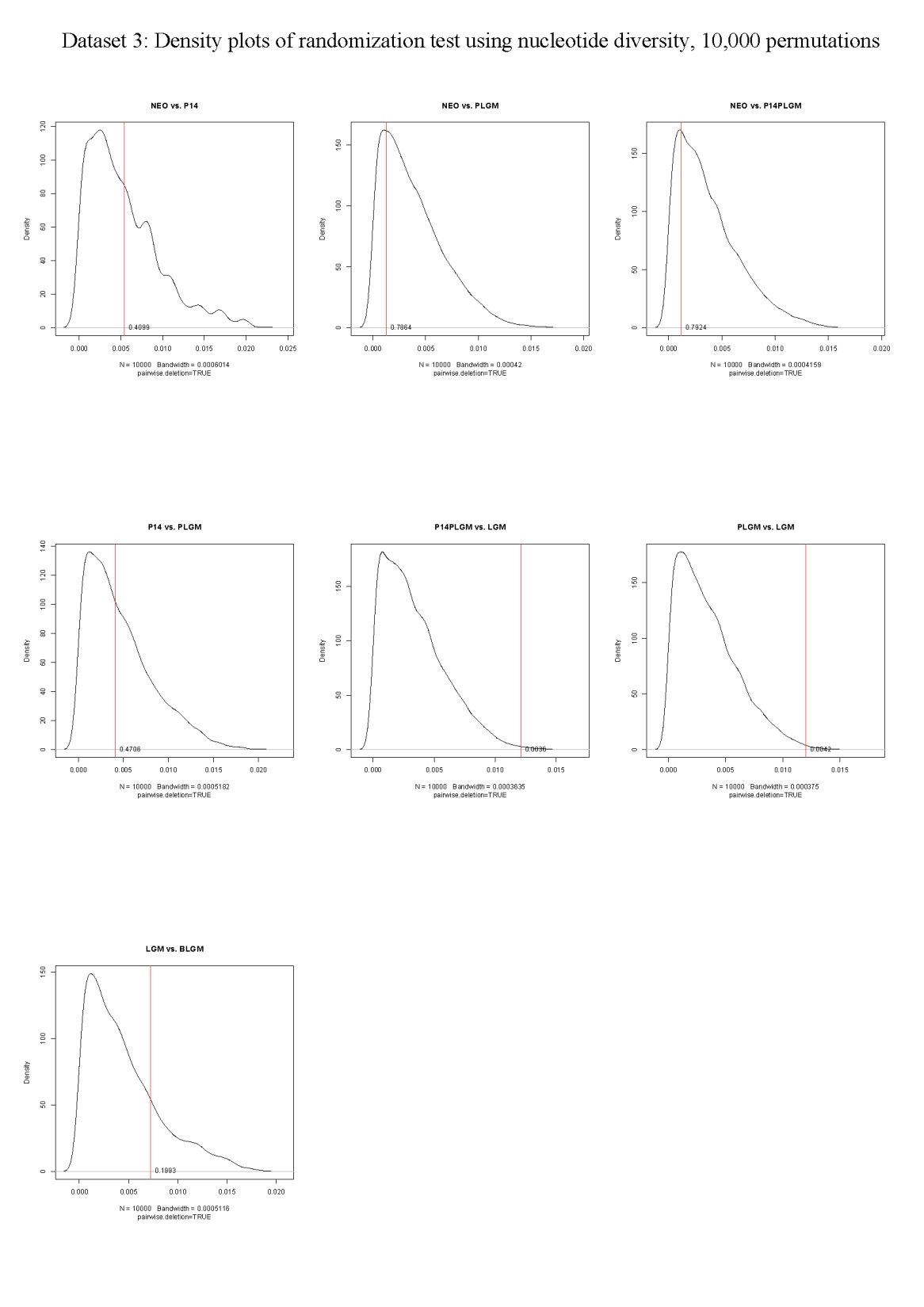

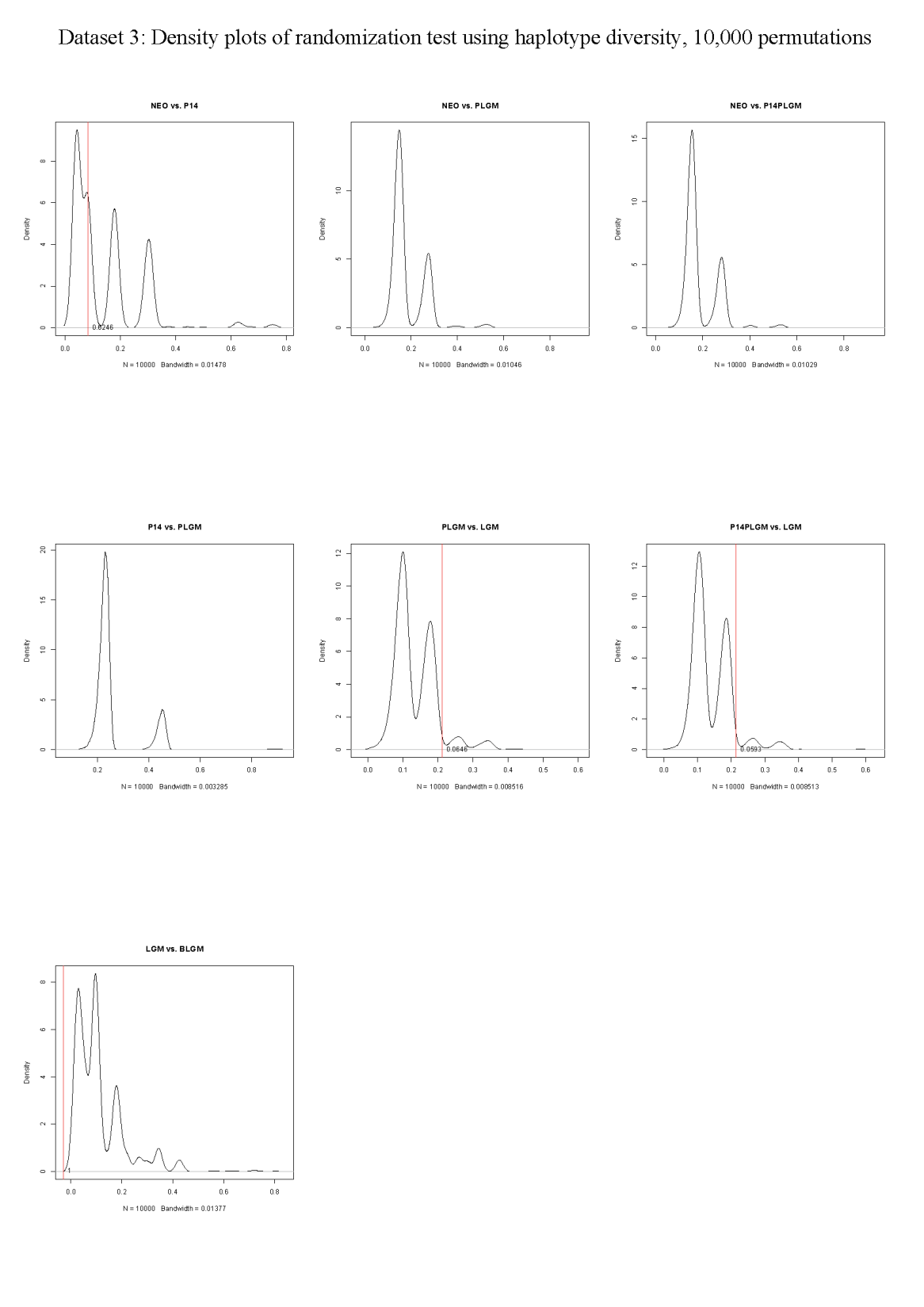


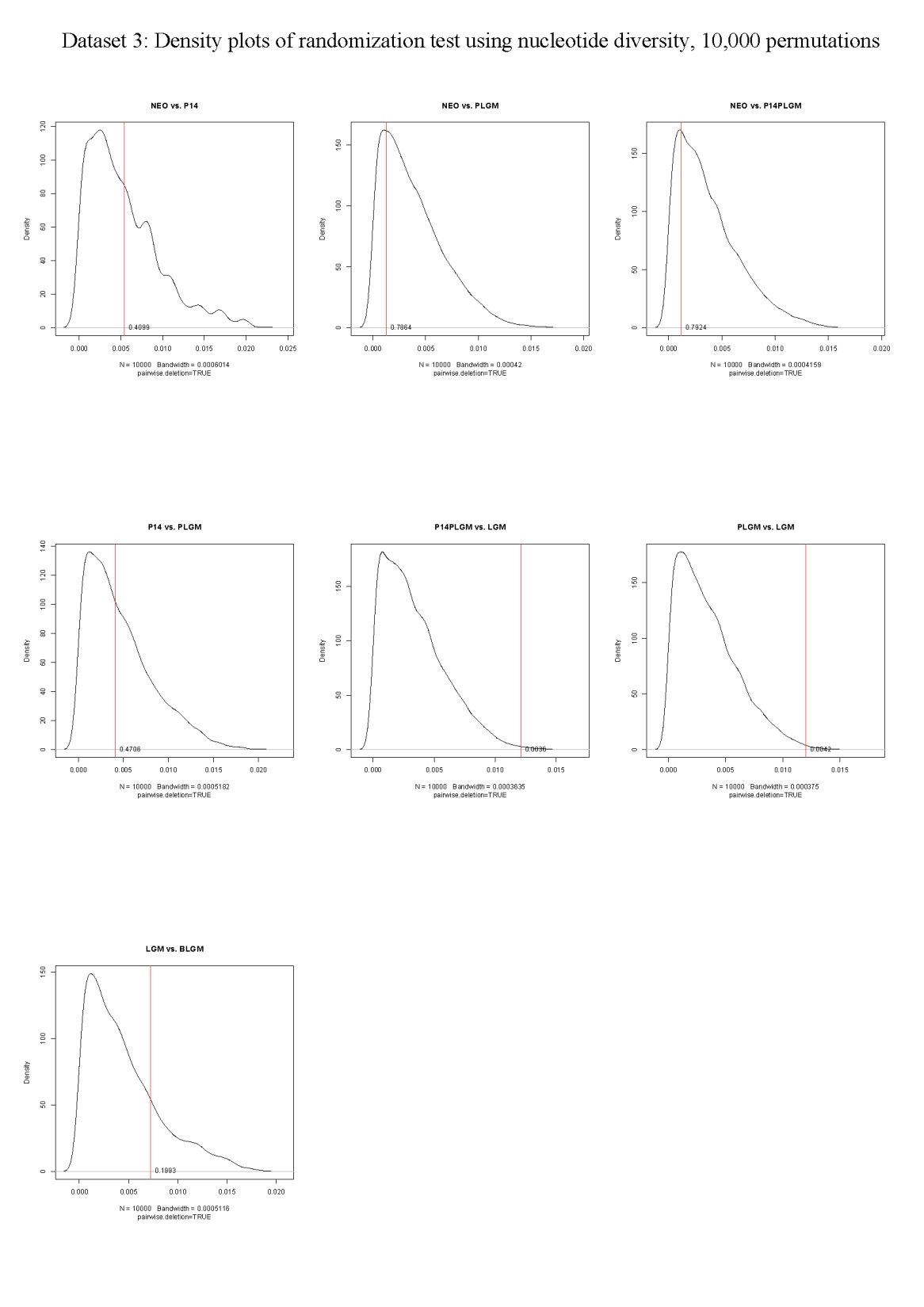

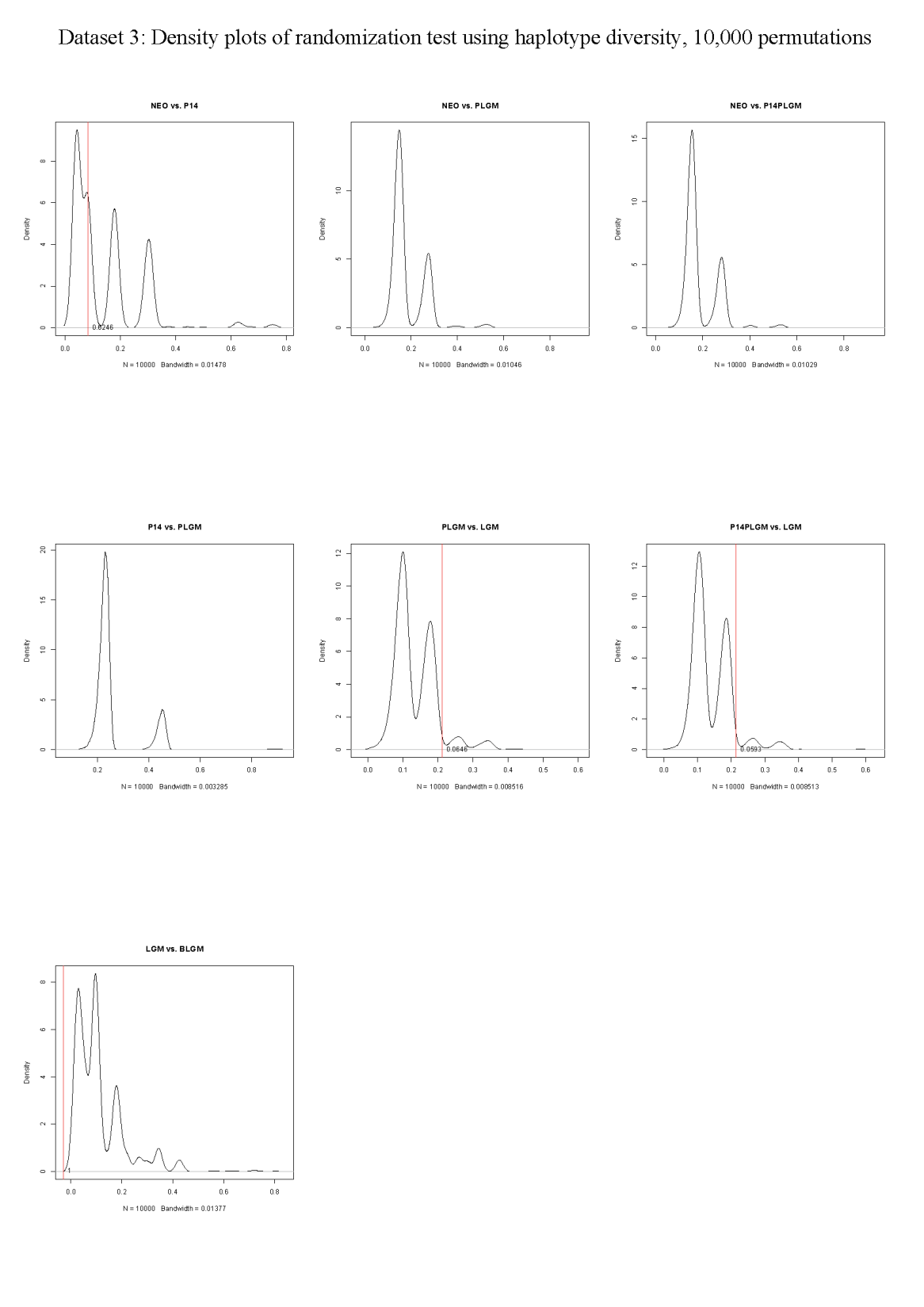


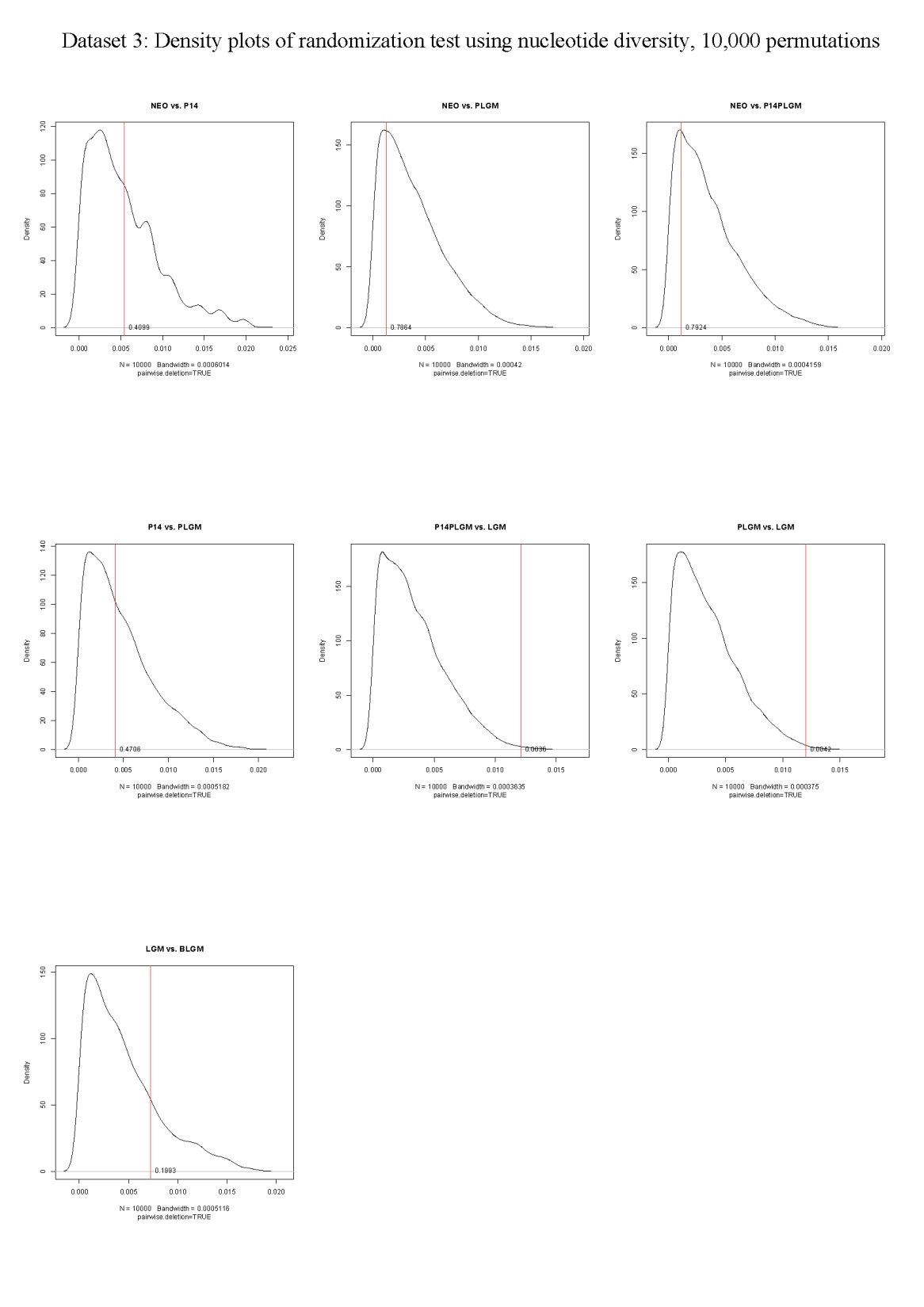

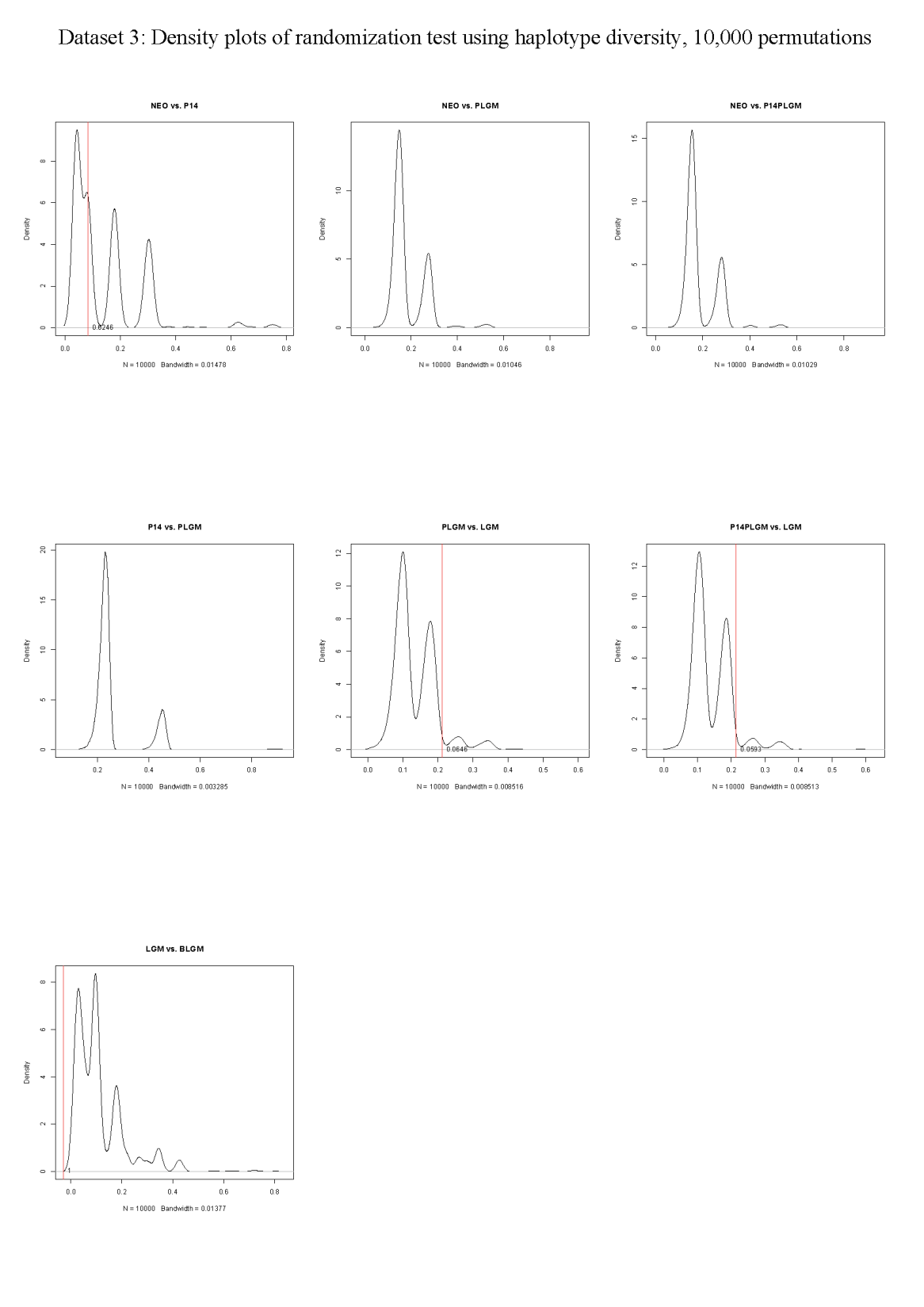


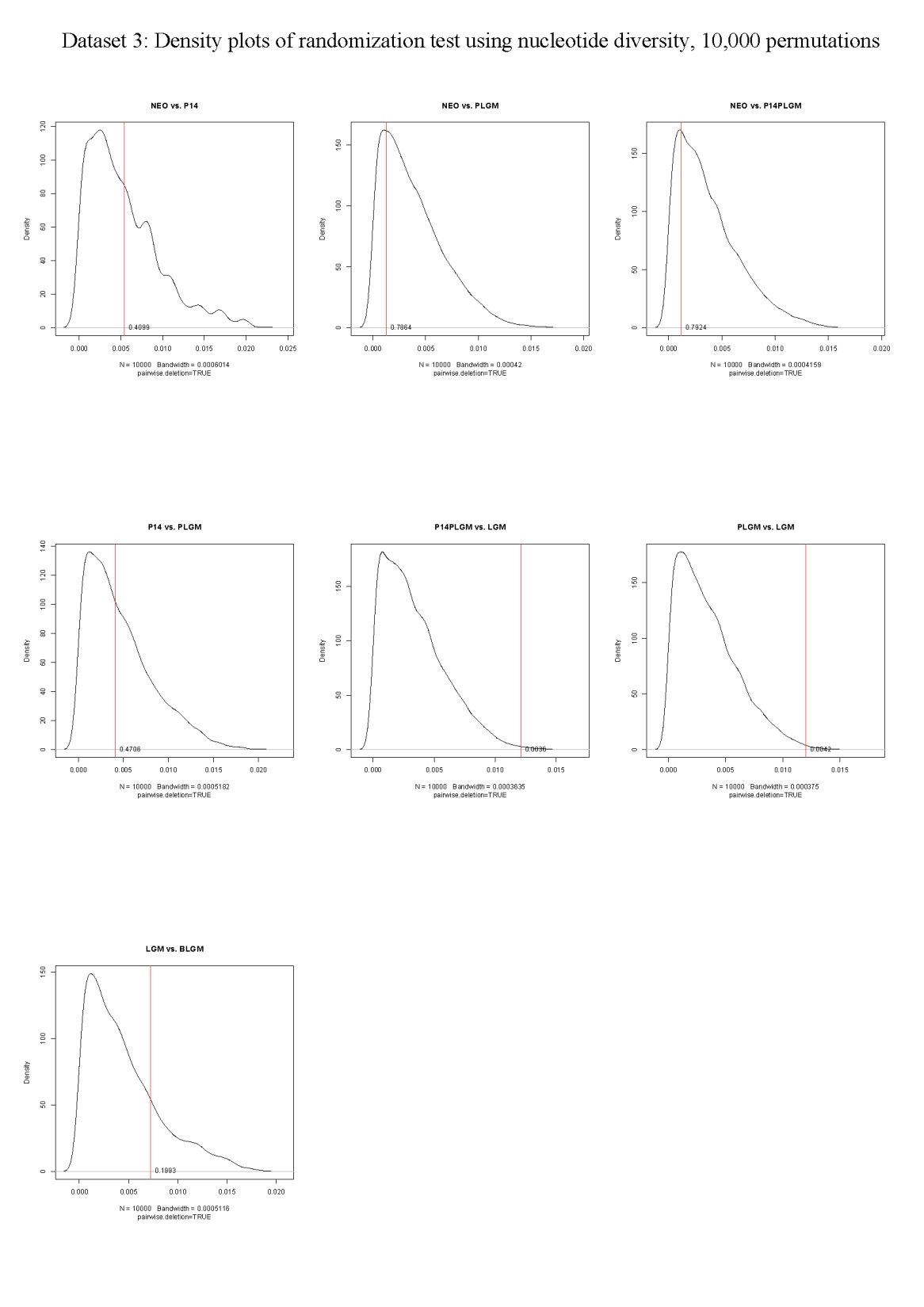

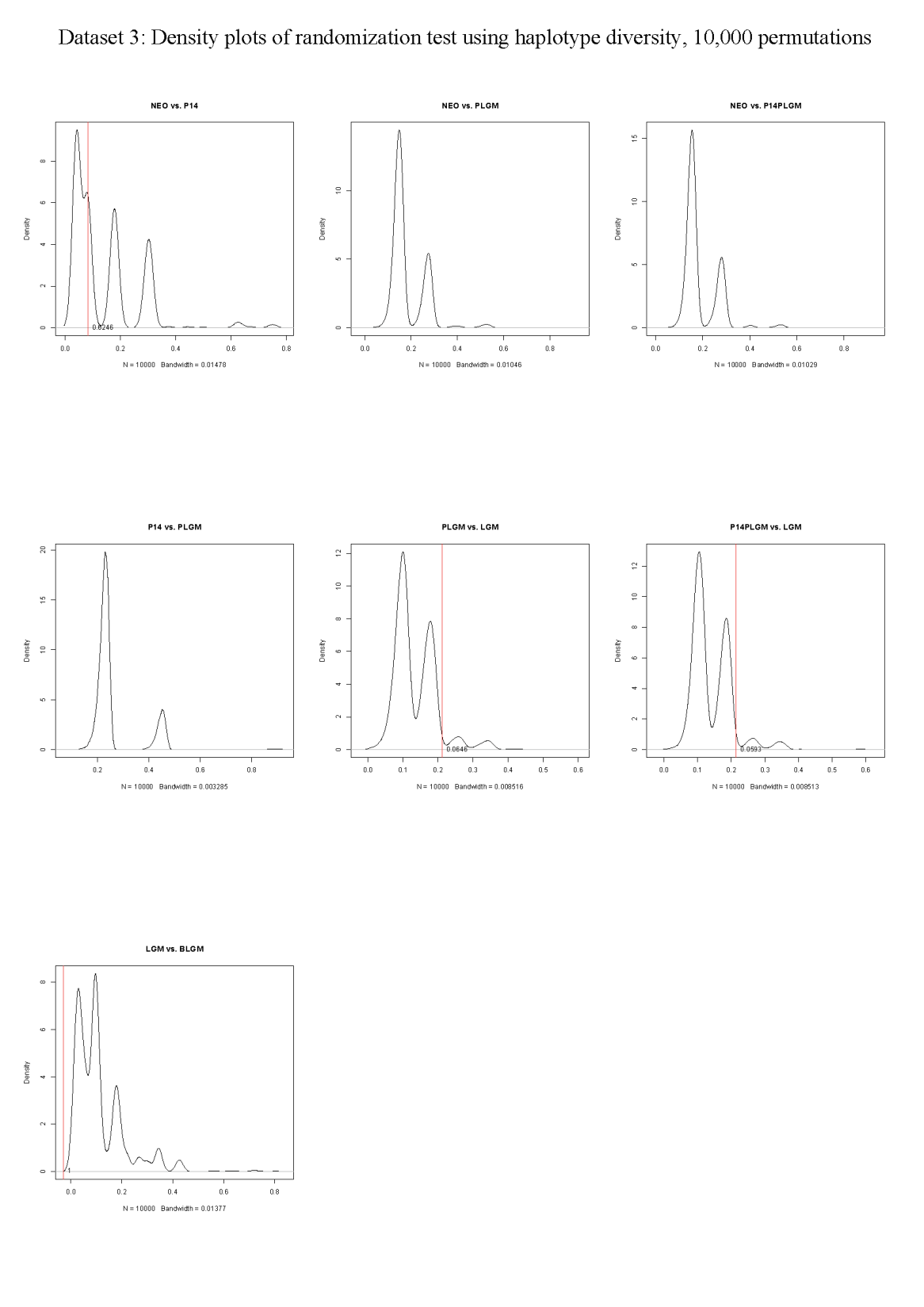


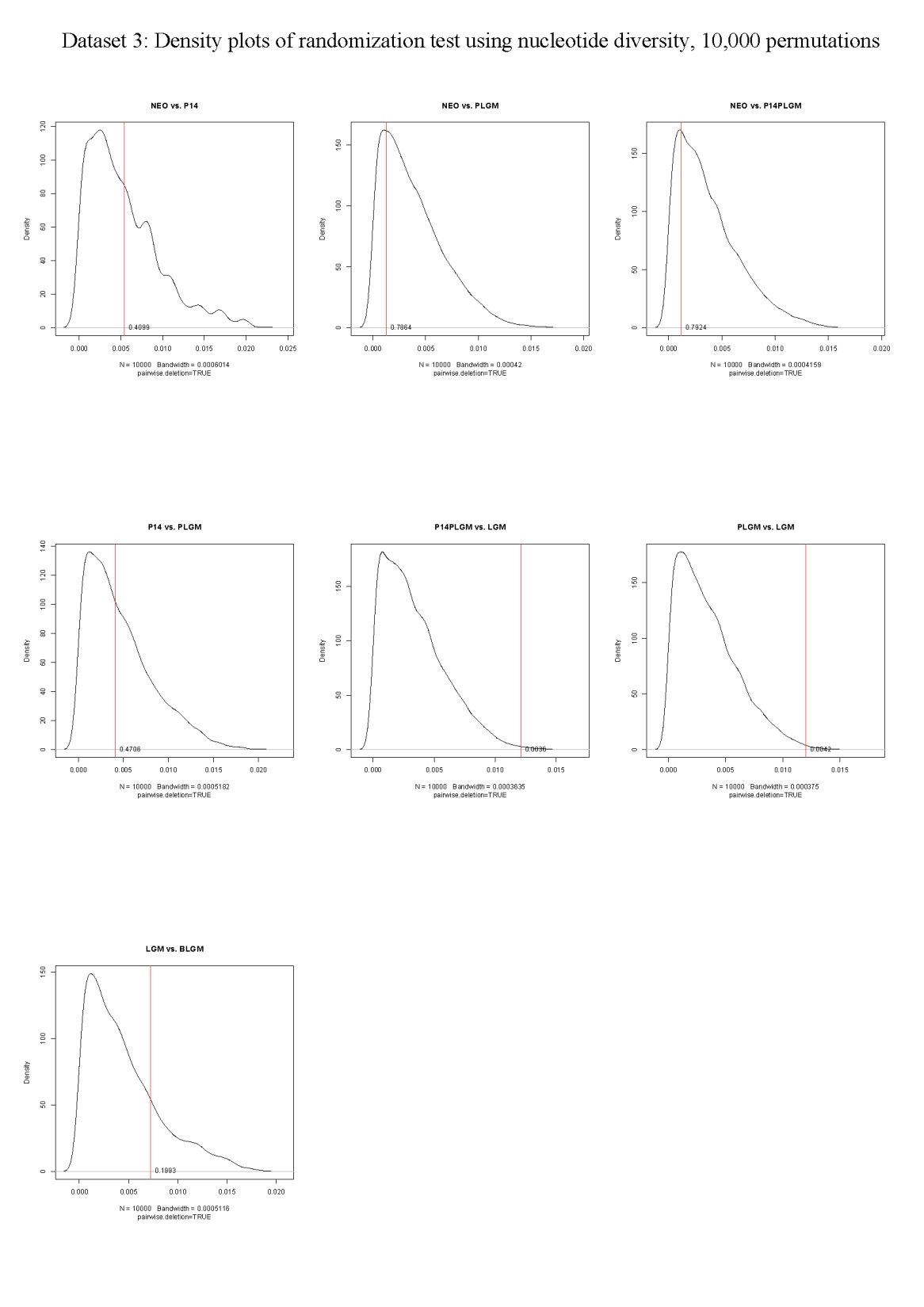

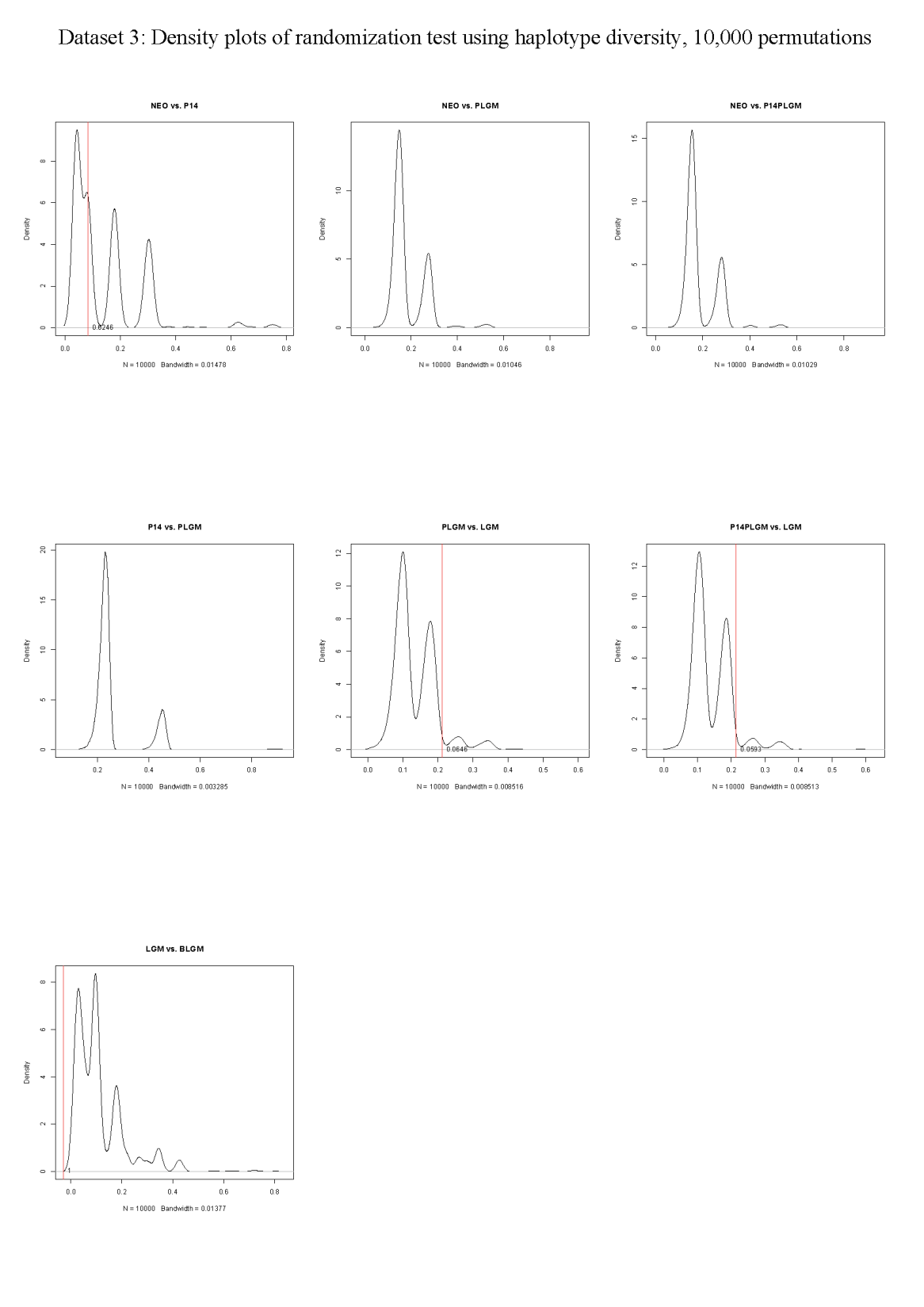

Supplement: S1 Fig — Unbiased and thus comparable pairs are framed. A: Dataset 1; B: Dataset 2; C: Dataset 3. (DOCX) [file pone.0177458.s001.docx]
