# Supplementary material for: Ancient mtDNA diversity reveals specific population development of wild horses in Switzerland after the Last Glacial Maximum
Source: PLoS One. 2017 May 24;12(5):e0177458. doi: 10.1371/journal.pone.0177458 (PMC5443500; doi:10.1371/journal.pone.0177458)
Supplement: S2 Fig — X-axis: pairwise differences, y-axis: number of pairs. A: Dataset 1; B: Dataset 2; C: Dataset 3. (DOCX) [file pone.0177458.s002.docx]

S2 Fig: Mismatch distribution (observed, bold line, and expected, dashed line) within time bins. X-axis: pairwise differences, y-axis: number of pairs. A: Dataset 1; B: Dataset 2; C: Dataset 3.

S2 Fig A: Mismatch distribution (observed, bold line, and expected, dashed line) within time bins. X-axis: paiwise differences, y-axis: number of pairs. Dataset 1.

S2 Fig B: Mismatch distribution (observed, bold line, and expected, dashed line) within time bins. X-axis: paiwise differences, y-axis: number of pairs. Dataset 2.

S2 Fig C: Mismatch distribution (observed, bold line, and expected, dashed line) within time bins. X-axis: paiwise differences, y-axis: number of pairs. Dataset 3.
