# Supplementary material for: Ancient mtDNA diversity reveals specific population development of wild horses in Switzerland after the Last Glacial Maximum
Source: PLoS One. 2017 May 24;12(5):e0177458. doi: 10.1371/journal.pone.0177458 (PMC5443500; doi:10.1371/journal.pone.0177458)
Supplement: S3 Fig — Swiss/Swabian samples: dataset 2. Rejections of null hypothesis are framed blue (Swiss LGM vs. Asia BLGM 0.0208; Swiss LGM vs. Swiss PLGM 0.034). (DOCX) [file pone.0177458.s003.docx]

S3 Fig: Density plots of randomization of Eurasian Pleistocene horse sample groups (10 k permutations with replacement) based on nucleotide diversity. Swiss/Swabian samples: dataset 2. Rejections of null hypothesis are framed blue (Swiss LGM vs. Asia BLGM 0.0208; Swiss LGM vs. Swiss PLGM 0.034).
