# Supplementary material for: Ancient mtDNA diversity reveals specific population development of wild horses in Switzerland after the Last Glacial Maximum
Source: PLoS One. 2017 May 24;12(5):e0177458. doi: 10.1371/journal.pone.0177458 (PMC5443500; doi:10.1371/journal.pone.0177458)
Supplement: S4 Fig — (DOCX) [file pone.0177458.s004.docx]

S4 Fig: Influential haplogroups (loadings) of component 1 (left panel) and 2 (right panel) for PCA graph.
