# Supplementary material for: Ancient mtDNA diversity reveals specific population development of wild horses in Switzerland after the Last Glacial Maximum
Source: PLoS One. 2017 May 24;12(5):e0177458. doi: 10.1371/journal.pone.0177458 (PMC5443500; doi:10.1371/journal.pone.0177458)
Supplement: S1 Table — (DOCX) [file pone.0177458.s005.docx]

S1 Table: Details of investigated sites, including site context, main references, location, laboratory and archaeological code, skeletal element, GenBank accession code, and dates [[extended from 29](#_ENREF_29)].

| **Name of site** | | **Site context** | **Reference** | **Location (alt., lat., lon.)** | **Samples** | | | | | | **Age** | | | |
| --- | --- | --- | --- | --- | --- | --- | --- | --- | --- | --- | --- | --- | --- | --- |
|  |  |  |  |  | **Lab code** | | **Arch code** | **Skeletal element** | **Genbank accession code** | **Haplogroup** | **Direct from sample** | | **From other specimens** | |
|  |  |  |  |  |  |  |  |  |  |  | **BP** | **calBP** | **BP** | **calBP** |
| 1 | Schalbergfelsenhöhle | Limestone cave used by different species and at different times. | ([57](#_ENREF_57)) | 425m, 47.47, 7.57 | PS1 | | Sch.795 | Pre(Molar) | KC893841 | A | ETH39763 34980±330 | 40032±856 |  | |
|  |  |  |  |  | PS2 | | Sch.833 | Pre(Molar) | KC893842 | A | ETH49373 32935±268 | 37414±676 |  |  |
|  |  |  |  |  | PS4 | | Sch.806 | Pre(Molar) | KC893843 | X3 | ETH39764 35995±320 | 41344±296 |  |  |
|  |  |  |  |  | PS5 | | Sch.805 | Pre(Molar) | KC893844 | B | ETH49374 10544±41 | 12511±140 |  |  |
| 2 | Kohlerhöhle | Cave dwelling with two distinct cultural layers covered with sinter. | ([58](#_ENREF_58), [59](#_ENREF_59)) | 378m, 47.43, 7.57 | KOH1 | KOHI* | A.3.L. | Premolar 3/4 inf. sin. | KC893804 | B | ETH39760 11525±60 | 13423±117 | B-4971 11640±150 | 13533±188^b^ |
|  |  |  |  |  | KOH2 |  | A.3.L. | Molar 1/2 inf. dext. |  |  |  |  | B-4969 12820±160 | 15401±471^b^ |
|  |  |  |  |  | KOH3 |  | A.3.L. | Molar 1/2 inf. sin. |  |  |  |  | ETH-43309 12460±45 | 14751±297^c^ |
|  |  |  |  |  | KOH5 |  | A.3.L. | Molar 3 inf. dext. |  |  |  |  | ETH-43310 16205±55 | 19350±289^c^ |
|  |  |  |  |  | KOH6 | KOHII* | - | Incisive dec. | KC893805 | A |  |  |  |  |
|  |  |  |  |  | KOH9 |  | H.a. | Premolar dec.3 inf. dext. |  |  | ETH39761 12790±45 | 15270±248 |  |  |
|  |  |  |  |  | KOH13 | KOHIII* | - | Incisive | KC893806 | B |  |  |  |  |
|  |  |  |  |  | KOH18 |  | - | Premolar 2 sup. dext. |  |  | ETH39762 12465±40 | 14761±293 |  |  |
|  |  |  |  |  | KOH4 | | A.6.r | Molar 3 inf. sin. | KC893807 | D |  |  |  |  |
|  |  |  |  |  | KOH7 | | H.a.g. | Incisive | KC893808 | A |  |  |  |  |
|  |  |  |  |  | KOH8 | | H.a. | Incisive | KC893809 | A |  |  |  |  |
|  |  |  |  |  | KOH10 | | H.a. | Incisive | KC893810 | K |  |  |  |  |
|  |  |  |  |  | KOH11 | | P.2.L. | Incisive | KC893811 | A |  |  |  |  |
|  |  |  |  |  | KOH12 | | H.a. | Incisive | KC893812 | A |  |  |  |  |
|  |  |  |  |  | KOH17 | | - | Premolar 3/4 dec. sup. sin. | KC893813 | B |  |  |  |  |
|  |  |  |  |  | KOH19 | | - | Maxilla dext., Premolars 2-3, Molars 1-3 | KC893814 | H |  |  |  |  |
|  |  |  |  |  | KOH20 | | Prof. 13-14 | Maxilla sin., Premolars 2-3, Molars 1-3 | KC893815 | H |  |  |  |  |
|  |  |  |  |  | KOH21 | | Prof. 9-10 L.o. | Molar sup. | KC893816 | X3 |  |  |  |  |
|  |  |  |  |  | KOH22 | | Prof. 14-16 Sch. I | Molar sup. | KC893817 | X3 | ETH44383 19615±75 | 23406±338 |  |  |
|  |  |  |  |  | KOH23 | | P.12.L.u. | Molar sup. | KC893818 | X3 | ETH44381 19810±65 | 23721±275 |  |  |
|  |  |  |  |  | KOH24 | | P.11.L.u. | Molar sup. | KC893819 | X3 | ETH44380 19970±70 | 23897±315 |  |  |
|  |  |  |  |  | KOH25 | | Prof. 14-16 Sch. II | Molar sup. | KC893820 | X3 |  |  |  |  |
|  |  |  |  |  | KOH26 | | P.1.L.u.B. | Molar sup. | KC893821 | A | ETH44378 12765±40 | 15223±258 |  |  |
|  |  |  |  |  | KOH27 | | Prof. 13 M-u. | Molar sup. | KC893822 | X3 | ETH44382 19730±75 | 23610±251 |  |  |
|  |  |  |  |  | KOH28 | | Prof. 14-16 Sch. II | Molar sup. | KC893823 | X3 |  |  |  |  |
|  |  |  |  |  | KOH29 | | P.12 L.u. | Molar sup. | KC893824 | B |  |  |  |  |
|  |  |  |  |  | KOH30 | | Prof. 10 R.m. | Molar sup. | KC893825 | X3 | ETH44379 19305±75 | 23067±265 |  |  |
|  |  |  |  |  | KOH31 | | P.0-1 | Molar sup. | KC893826 | A |  |  |  |  |
| 3 | Kesslerloch | Limestone cave dwelling, layers covered with sinter. | ([60-63](#_ENREF_60)) | 440m, 47.75, 8.69 | KES1 | | KESLH 33 | Metapodial | KC893761 | X4b |  |  | B-3327 11220±180 | 13120±198^d^ |
|  |  |  |  |  | KES2 | | KESLH 33.2 | Metapodial | KC893762 | X4b |  |  | KIA-33350 12225±45 | 14277±239^e^ |
|  |  |  |  |  | KES3 | | KESLH 26 II s | Incisive | KC893763 | B |  |  | KIA-33351 12335±45 | 14502±338^e^ |
|  |  |  |  |  | KES5 | | KESLH 21 II c | Incisive | KC893764 | X4b |  |  | KIA-11826 12502±52 | 14819±295^e^ |
|  |  |  |  |  | KES8 | | KESLH 33_21 | Metapodial | KC893765 | A/B/C/D/F |  |  | OxA-5748 12770±90 | 15213±302^f^ |
|  |  |  |  |  | KES9 | | KESLH 33_22 | Metapodial | KC893766 | B |  |  | KIA-11825 12774±54 | 15237±262^e^ |
|  |  |  |  |  | KES10 | | KESLH 38_44 | Metapodial | KC893767 | A/B/C/D/F |  |  | KIA-11829 12897±53 | 15591±329^e^ |
|  |  |  |  |  | KES11 | | KESLH 38_45 | Metapodial | KC893768 | X4b |  |  | Hv-10652 12890±90 | 15595±380^g^ |
|  |  |  |  |  | KES13 | | KESLH 38 | Metapodial | KC893769 | K |  |  | B-3329 12970±180 | 15798±512^d^ |
|  |  |  |  |  | KES16 | | KESLH 38 | Metapodial | KC893770 | A/B/C/D/F |  |  | KIA-11827 13052±53 | 15941±390^e^ |
|  |  |  |  |  | KES17 | | KESLH 11 | Incisive | KC893771 | X4b |  |  | OxA-5746 13120±90 | 16038±401^f^ |
|  |  |  |  |  | KES18 | | KESLH 26 | (Pre)molar M3/P2 | KC893772 | X3 |  |  | OxA-5747 13430±100 | 16367±427^f^ |
|  |  |  |  |  | KES19 | | KESLH 26 | (Pre)molar M3/P2 | KC893773 | X4b |  |  | OxA-5750 13670±100 | 16745±270^f^ |
|  |  |  |  |  | KES20 | | KESLH 26 | (Pre)molar M3/P2 | KC893774 | B |  |  | KIA-11828 13858±55 | 17085±180^e^ |
|  |  |  |  |  | KES21 | | KESLH 26 | (Pre)molar M3/P2 | KC893775 | H |  |  | OxA-10239 13980±110 | 17234±228 |
|  |  |  |  |  | KES27 | | KESLH 38 | Molar 3 inf. dext. | KC893776 | B |  |  | OxA-5749 14150±100 | 17392±254^f^ |
|  |  |  |  |  | KES29 | | KESLH 36 3 B s | Molar 3 inf. dext. | KC893777 | A | ETH44387 13035±60 | 15915±396 | OxA-10238 14330±110 | 17505±261^e^ |
|  |  |  |  |  | KES31 | | KESLH 32 III A c | Molar sup. | KC893778 | A |  |  | OxA-10298 15020±180 | 18254±263^e^ |
|  |  |  |  |  | KES34 | | KESLH 13 I s | Molar sup. sin. | KC893779 | A/B/C/D/F | ETH44388 12605±50 | 14962±295 |  |  |
|  |  |  |  |  | KES35 | | KESLH 34 III B c lower part | Molar inf. | KC893780 | A/B/C/D/F |  |  |  |  |
|  |  |  |  |  | KES37 | | KESLH 34 III B c | Molar sup. | KC893781 | B |  |  |  |  |
|  |  |  |  |  | KES38 | | KESLH 34 III B c | Molar sup. | KC893782 | A/B/C/D/F |  |  |  |  |
|  |  |  |  |  | KES39 | | KESLH 26 II s | Molar inf.dext. | KC893783 | X4b |  |  |  |  |
|  |  |  |  |  | KES41 | | KESLH 11; HE11 | Molar sup. dext. | KC893784 | B | ETH44385 13690±50 | 16824±201 |  |  |
|  |  |  |  |  | KES42 | | KESLH 34 III B c | Molar sup. | KC893785 | D |  |  |  |  |
|  |  |  |  |  | KES43 | | KESLH 34_20 | Metapodial | KC893786 | A/B/C/D/F |  |  |  |  |
|  |  |  |  |  | KES46 | | KESLH 34; HE34 | Molar inf., small | KC893787 | A/B/C/D/F |  |  |  |  |
|  |  |  |  |  | KES47 | | KESLH 26 II s | Molar sup. | KC893788 | X4b |  |  |  |  |
|  |  |  |  |  | KES48 | | KESLH 26 II s | Molar sup. | KC893789 | A |  |  |  |  |
|  |  |  |  |  | KES49 | | KESLH 24 II u | Metapodial | KC893790 | B |  |  |  |  |
|  |  |  |  |  | KES50 | | KESLH 24 II u | Metapodial | KC893791 | K |  |  |  |  |
|  |  |  |  |  | KES51 | | KESLH 12 I u | Metapodial | KC893792 | H |  |  |  |  |
|  |  |  |  |  | KES53 | | KESLH 33 III A s | Molar sup. dext. | KC893793 | K | ETH44384 12885±65 | 15560±333 |  |  |
|  |  |  |  |  | KES54 | | KESLH 24; HE 24 II u | Molar inf. sin. | KC893794 | X4b |  |  |  |  |
|  |  |  |  |  | KES55 | | KESLH 24; HE 24 II u | Molar inf. sin. | KC893795 | X4b |  |  |  |  |
|  |  |  |  |  | KES56 | | KESLH 24; HE 24 II u | Molar inf. sin. | KC893796 | A/B/C/D/F |  |  |  |  |
|  |  |  |  |  | KES58 | | KESLH 33 III A s | Molar sup. sin., small | KC893797 | X3 |  |  |  |  |
|  |  |  |  |  | KES59 | | KESLH 34 III B c | Molar inf. dext. | KC893798 | B |  |  |  |  |
|  |  |  |  |  | KES61 | | KESLH AG22 | Metacapal small | KC893799 | A/B/C/D/F |  |  |  |  |
|  |  |  |  |  | KES62 | | KESLH AG19 | Metatarsal | KC893800 | K/X4b |  |  |  |  |
|  |  |  |  |  | KES63 | | KESLH 21 II c | Molar inf. | KC893801 | A |  |  |  |  |
|  |  |  |  |  | KES66 | | KESLH 24 II n | Molar sup. sin. | KC893802 | H | ETH44386 12795±55 | 15277±254 |  |  |
|  |  |  |  |  | KES67 | | KESLH 24 II n | Molar sup. sin. | KC893803 | X3 |  |  |  |  |
| 4 | Käsloch | Limestone cave dwelling. | ([64](#_ENREF_64), [65](#_ENREF_65)) | 420m, 47.37, 7.91 | KL1 | | 127 6 7138 | Molar | KC893827 | B | ETH39769 12505±45 | 14825±291 |  |  |
|  |  |  |  |  | KL3 | | 127 6 7102 | Molar | KC893828 | A/B/C/D/F | ETH39770 13760±45 | 16931±154 |  |  |
|  |  |  |  |  | KL4 | | 127 6 7140 | Molar | KC893829 | H | ETH39771 12450±45 | 14719±313 |  |  |
| 5 | Rislisberghöhle | Limestone cave dwelling. | ([66](#_ENREF_66), [67](#_ENREF_67)) | 488m, 47.3, 7.7 | RIS3 | | 707 | Dec. molar inf. sin. | KC893831 | K | ETH39768 10770±45 | 12749±50 | Ly-1099 11860±230 | 13868±346^b^ |
|  |  |  |  |  | RIS6 | | 103/28 Fk252 Feld66 | (Pre)molar | KC893832 | K |  |  | ETH-42516 12680±45 | 15064±301^c^ |
|  |  |  |  |  | RIS7 | | 103/28 Fk1133 | (Pre)molar | KC893833 | K |  |  | ETH-42515 12710±45 | 15112±296^c^ |
|  |  |  |  |  | RIS13 | | 103/28 Fk1130 | (Pre)molar | KC893834 | K | ETH44377 12575±55 | 14924±293 | ETH-42517 13000±50 | 15856±398^c^ |
| 6 | Schweizersbild | Rockshelter. | ([68](#_ENREF_68), [69](#_ENREF_69)) | 472m, 47.72, 8.64 | SB1 | | Yellow cultural layer | Molar inf. | KC893835 | B |  |  |  |  |
|  |  |  |  |  | SB2 | | Yellow cultural layer | Molar inf. | KC893836 | B |  |  |  |  |
|  |  |  |  |  | SB6 | | Yellow cultural layer | (Pre)molar inf. | KC893837 | A | ETH44390 12240±60 | 14308±258 |  |  |
|  |  |  |  |  | SB7 | | - | Molar inf. | KC893838 | B | ETH44392 12690±50 | 15077±33 |  |  |
|  |  |  |  |  | SB10 | | - | Molar sup. | KC893839 | A |  |  |  |  |
|  |  |  |  |  | SB11 | | Lower rodent layer | Molar sup. | KC893840 | X3 | ETH44391 12240±50 | 14303±251 |  |  |
| 7 | Abri Neumühle | Rockshelter. | ([70](#_ENREF_70)) | 520m, 47.44, 7.33 | RAN1 | | NM o.B. 89.1.A | Molar | KC893830 | C | ETH44393 12305±40 | 14439±324 |  |  |
| 8 | Twann-Bahnhof | Lacustrine settlement, transgressed. | ([71](#_ENREF_71)) | 426m, 47.09,7.17 | TWB3 | | 1009.6 | Metatarsus III dext. dist. | KC893755 | B | ETH39773 4800±35 | 5540±46 | dendrochronological layer date 3666±35 BC |  |
|  |  |  |  |  | TWB4 | | 167 | Phalanx I post. dext., Phalanx II, Humerus prox. Epiphysis sin. | KC893756 | D |  |  | dendrochronological layer date 3598±25 BC |  |
|  |  |  |  |  | TWB5 | | 909.21 Layer E6 | Phalanx I ant. sin. | KC893757 | B | ETH39774 4740±35 | 5467±97 | dendrochronological layer date 3598±25 BC |  |
|  |  |  |  |  | TWB9 | | 1723.5 | Premolar dec.2 inf. dext. | KC893758 | B |  |  | dendrochronological layer date 3666±35 BC |  |
|  |  |  |  |  | TWB10 | | 1247 V-10 | Scapula sin. dist. | KC893759 | A/B/C/D/F |  |  | dendrochronological layer date 3666±35 BC |  |
|  |  |  |  |  | TWB13 | | 1127 V-10 | Sesamoid | KC893760 | A |  |  | dendrochronological layer date 3666±35 BC |  |
| 9 | Mumpf | Settlement. | ([72](#_ENREF_72)) | 287m, 47.55, 7.92 | MU1 | | 123 | Premolar 3/4 sup. | KC893753 | D | ETH36444 4525±55 | 5180±101^i^ |  |  |
|  |  |  |  |  | MU2 | | - | Molar 3 sup. | KC893754 | D | ETH36446 4395±60 | 5042±139^i^ |  |  |

^a^Bodenforschung Basel-Stadt, ^b^[[73](#_ENREF_73)], ^c^[[74](#_ENREF_74)], ^d^[[75](#_ENREF_75)], ^e^[[63](#_ENREF_63)], ^f^[[76](#_ENREF_76)], ^g^[[77](#_ENREF_77)], ^i^[[72](#_ENREF_72)].

*Multiple teeth presumably belonging to the same individual.
