# Supplementary material for: Ancient mtDNA diversity reveals specific population development of wild horses in Switzerland after the Last Glacial Maximum
Source: PLoS One. 2017 May 24;12(5):e0177458. doi: 10.1371/journal.pone.0177458 (PMC5443500; doi:10.1371/journal.pone.0177458)

S2 Table: Parameters for weighting of nucleotide positions for Median Joining Network analysis based on 97 Pleistocene horse mitochondrial d-loop sequences.


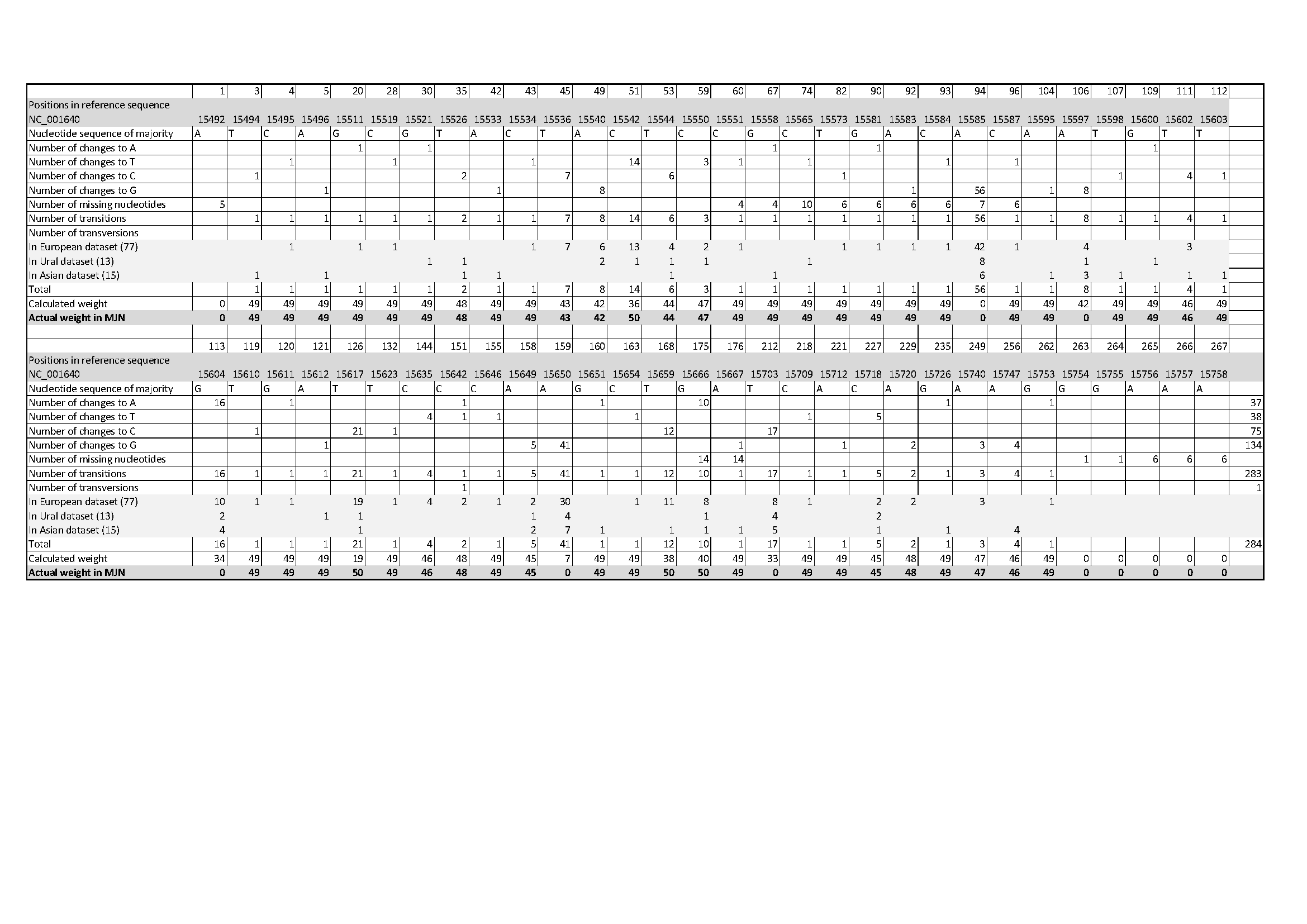

Supplement: S2 Table — (DOCX) [file pone.0177458.s006.docx]
