# Supplementary material for: Ancient mtDNA diversity reveals specific population development of wild horses in Switzerland after the Last Glacial Maximum
Source: PLoS One. 2017 May 24;12(5):e0177458. doi: 10.1371/journal.pone.0177458 (PMC5443500; doi:10.1371/journal.pone.0177458)
Supplement: S3 Table — Haplogroup defining nucleotide positions relative to the horse reference mitogenome [1] are shown according to their position. All deviations from the reference sequence are given, with mandatory defining positions in bold and optional nucleotide positions in parenthesis. Note that transitions on nucleotide positions 15,585; 15,604 and 15,650 occur sporadically in all haplogroups; these positions are regarded as hotspots and therefore dismissed. (DOCX) [file pone.0177458.s007.docx]

S3 Table: Details of haplogroups detected in Pleistocene horses from the Swiss and Swabian Jura region, nomenclature follows [2]. Haplogroup defining nucleotide positions relative to the horse reference mitogenome [1] are shown according to their position. All deviations from the reference sequence are given, with mandatory defining positions in bold and optional nucleotide positions in parenthesis. Note that transitions on nucleotide positions 15,585; 15,604 and 15,650 occur sporadically in all haplogroups; these positions are regarded as hotspots and therefore dismissed.

| Haplogroup | Nucleotide positions |
| --- | --- |
| A | **15,495; 15,602; 15,720** |
| B | 15,495; 15,602; **15,617**; (**15,635**); (**15,649**); (**15,659**); 15,720 |
| C | 15,495; 15,602; (**15,649**); 15,720 |
| D | 15,495 |
| H | 15,495; **15,536**; 15,602; 15,720 |
| K | 15,495; 15,602; **15,703**; 15,720; (**15,740**) |
| X3 | 15,495; **15,542**; (**15,544**); 15,602; **15,666**; 15,720 |
| X4b | 15,495; **15,540**; 15,602; (**15,718**); 15,720 |
