# Supplementary material for: Ancient mtDNA diversity reveals specific population development of wild horses in Switzerland after the Last Glacial Maximum
Source: PLoS One. 2017 May 24;12(5):e0177458. doi: 10.1371/journal.pone.0177458 (PMC5443500; doi:10.1371/journal.pone.0177458)
Supplement: S4 Table — (DOCX) [file pone.0177458.s008.docx]

S4 Table: Nucleotide and haplotype diversities in horse populations from Switzerland and the Swabian Jura (all datasets).

| Time period | Dataset 1 | | | | Dataset 2 | | | | Dataset 3 | | | |
| --- | --- | --- | --- | --- | --- | --- | --- | --- | --- | --- | --- | --- |
|  | Number of samples | Number of haplotypes | Nucleotide diversity | Haplotype diversity | Number of samples | Number of haplotypes | Nucleotide diversity | Haplotype diversity | Number of samples | Number of haplotypes | Nucleotide diversity | Haplotype diversity |
| Palaeontological | 4 | 3 | 0.0111 | 0.83 | 4 | 4 | 0.0104 | 1 | 4 | 4 | 0.0104 | 1 |
| Badegoulian | 11 | 3 | 0.0089 | 0.47 | 11 | 4 | 0.0093 | 0.6 | 5 | 1 | 0 | 0 |
| Magdalenian | 70 | 14 | 0.0092 | 0.63 | 53 | 28 | 0.016 | 0.95 | 20 | 8 | 0.0091 | 0.7 |
| Magd. + Azilian | 74 | 14 | 0.0103 | 0.63 | 57 | 29 | 0.0159 | 0.95 | - |  | - | - |
| Azilian | 4 | 2 | 0.0111 | 0.67 | 4 | 3 | 0.0104 | 0.83 | 3 | 2 | 0.0111 | 0.67 |
| Neolithic | 8 | 3 | 0.0086 | 0.68 | 6 | 2 | 0.0149 | 0.6 | 4 | 2 | 0.0166 | 0.67 |
