# Supplementary material for: Ancient mtDNA diversity reveals specific population development of wild horses in Switzerland after the Last Glacial Maximum
Source: PLoS One. 2017 May 24;12(5):e0177458. doi: 10.1371/journal.pone.0177458 (PMC5443500; doi:10.1371/journal.pone.0177458)
Supplement: S5 Table — Lower triangle: FST values, upper triangle: p values. Comparable populations are boxed, significant FST values are in bold. (DOCX) [file pone.0177458.s009.docx]

S5 Table: F_ST_ values of pairwise populations (all datasets). Lower triangle: F_ST_ values, upper triangle: *p* values. Comparable populations are boxed, significant F_ST_ values are in bold.

|  | Dataset 1 | | | | | | Dataset 2 | | | | | | Dataset 3 | | | | |
| --- | --- | --- | --- | --- | --- | --- | --- | --- | --- | --- | --- | --- | --- | --- | --- | --- | --- |
|  | Palaeontological | Badegoulian | Magdalenian | Magd.+ Azilian | Azilian | Neolithic | Palaeontological | Badegoulian | Magdalenian | Magd.+ Azilian | Azilian | Neolithic | Palaeontological | Badegoulian | Magdalenian | Azilian | Neolithic |
| Palaeonto-logical | - | .009 | .3 | .3 | .4 | .1 | - | .01 | .6 | .5 | .2 | .1 | - | .008 | .5 | .08 | .2 |
| Badegoulian | **.4** | - | 0 | .0002 | .003 | .001 | **.3** | - | .0003 | .0003 | .002 | .0003 | **.77** | - | 0 | .01 | .008 |
| Magda-lenian | .01 | **.28** | - | .8 | .05 | .05 | 0 | **.16** | - | 1 | .1 | .0008 | 0 | **.68** | - | .03 | .003 |
| Magd.+ Azilian | .009 | **.26** | 0 | - | .1 | .07 | 0 | **.17** | 0 | - | .2 | .0006 | **-** | **-** | **-** | - | - |
| Azilian | .22 | **.5** | .17 | .09 | - | .06 | .17 | **.46** | .06 | .04 | - | .1 | .21 | **.85** | .25 | - | .2 |
| Neolithic | .13 | **.45** | .07 | .07 | .33 | - | .21 | **.5** | **.2** | **.2** | .26 | - | .17 | **.78** | **.29** | .21 | - |
