# Supplementary material for: Ancient mtDNA diversity reveals specific population development of wild horses in Switzerland after the Last Glacial Maximum
Source: PLoS One. 2017 May 24;12(5):e0177458. doi: 10.1371/journal.pone.0177458 (PMC5443500; doi:10.1371/journal.pone.0177458)
Supplement: S6 Table — Significant results are in bold. NaN = not a number because only one haplotype was present. (DOCX) [file pone.0177458.s010.docx]

S6 Table: Tajima’s *D*, Fu’s *F_S_*, sum of squared deviances (SSD) and Harpending’s raggedness index results for horse populations from Switzerland and the Swabian Jura (all datasets). Significant results are in bold. NaN = not a number because only one haplotype was present.

|  | Dataset 1 | | | | | | | | Dataset 2 | | | | | | | | Dataset 3 | | | | | | | |
| --- | --- | --- | --- | --- | --- | --- | --- | --- | --- | --- | --- | --- | --- | --- | --- | --- | --- | --- | --- | --- | --- | --- | --- | --- |
|  | Tajima’s *D* | *p* | Fu’s *F_S_* | *p* | SSD | *p* | Raggedness index | *p* | Tajima’s *D* | *p* | Fu’s *F_S_* | *p* | SSD | *p* | Raggedness index | *p* | Tajima’s *D* | *p* | Fu’s *F_S_* | *p* | SSD | *p* | Raggedness index | *p* |
| Palaeontol. | -0.21 | .6 | 0.56 | .5 | **0.27** | 0.03 | 0.75 | 0.2 | -0.8 | .2 | -1.51 | .06 | 0.03 | 0.8 | 0.11 | 0.9 | -0.8 | .2 | **-1.51** | .05 | 0.03 | 0.8 | 0.11 | 0.9 |
| Badegoulian | 0.19 | .6 | 2.34 | .9 | **0.33** | 0.0007 | 0.4 | 0.9 | -0.09 | .5 | 0.77 | .7 | 0.13 | 0.1 | 0.33 | .02 | NaN | NaN | NaN | NaN | NaN | NaN | NaN | NaN |
| Magdalenian | **-1.53** | .04 | **-8.39** | .0004 | **0.24** | 0.0003 | 0.03 | 1 | -1.32 | .07 | **-21.71** | 0 | 0.001 | 0.6 | 0.02 | 0.6 | **-1.53** | .049 | -2.3 | .07 | 0.07 | 0.1 | 0.25 | 0.09 |
| Magd.+Azil. | -1.35 | .07 | **-7.29** | .001 | 0.02 | 0.4 | 0.1 | 0.5 | -1.29 | .08 | **-22.59** | 0 | 0.001 | 0.63 | 0.02 | 0.6 | - | - | - | - | - | - | - | - |
| Azilian | 2.08 | 1 | 2.72 | .9 | **0.37** | 0.02 | 1 | 0.3 | 1.37 | .9 | 0.46 | .5 | 0.08 | 0.33 | 0.25 | 0.7 | 0 | .8 | 2.02 | .8 | 0.37 | 0.05 | 1 | 0.6 |
| Neolithic | 0.58 | .8 | 0.72 | .6 | 0.06 | 0.2 | 0.23 | 0.3 | 2.12 | 1 | 4.51 | 1 | **0.38** | 0.02 | **0.88** | 0.03 | 2.16 | 1 | 3.53 | .9 | **0.41** | 0.02 | 1 | 0.3 |
