# Supplementary material for: Ancient mtDNA diversity reveals specific population development of wild horses in Switzerland after the Last Glacial Maximum
Source: PLoS One. 2017 May 24;12(5):e0177458. doi: 10.1371/journal.pone.0177458 (PMC5443500; doi:10.1371/journal.pone.0177458)
Supplement: S7 Table — Sequences marked with an ‘a’ are part of draft full genomes which are obtainable as SRA-Illumina runs on GenBank. (DOCX) [file pone.0177458.s011.docx]

S7 Table: Sequences of published Pleistocene horses from Eurasia. Sequences marked with an ‘^a^’ are part of draft full genomes which are obtainable as SRA-Illumina runs on GenBank.

| Origin | Date | GenBank accession code | Reference |
| --- | --- | --- | --- |
| Germany (south) | 50-14,000 BP | DQ007558/DQ007611; DQ007556/DQ007609; DQ007591; DQ007590 | Weinstock et al. (2005) |
|  |  | FJ204352 | Cieslak et al. (2010) |
| Russian Federation (Taymyr peninsula) | 43-16,000 BP | upon request ^a^ | Orlando et al. (2013) |
| Russian Federation (Novosibirsk islands, Sakha Republic) | 40-20,000 BP | upon request ^a^ | Orlando et al. (2013) |
|  |  | JN570964-JN570966 | Lorenzen et al. (2011) |
|  |  | FJ204314-FJ204318 | Cieslak et al. (2010) |
| Russian Federation (Lena river delta, Sakha Republic) | 36-31,000 BP | JN570958; JN570959 | Lorenzen et al. (2011) |
|  |  | DQ007577 | Weinstock et al. (2005) |
| Russian Federation (Kolyma lowlands, Sakha Republic) | 38-17,000 BP | JN570957; JN570961 | Lorenzen et al. (2011) |
| Russian Federation (Yana river basin, Sakha Republic) | 22,500 BP | JN570960 | Lorenzen et al. (2011) |
| Russian Federation (Urals) | 46-17,000 BP | JN570954; JN570972-JN570982; JN570993 | Lorenzen et al. (2011) |
