# Supplementary material for: Ancient mtDNA diversity reveals specific population development of wild horses in Switzerland after the Last Glacial Maximum
Source: PLoS One. 2017 May 24;12(5):e0177458. doi: 10.1371/journal.pone.0177458 (PMC5443500; doi:10.1371/journal.pone.0177458)
Supplement: S8 Table — (DOCX) [file pone.0177458.s012.docx]

S8 Table: Nucleotide and haplotype diversity of Eurasian Pleistocene horses based on pairwise deletion of missing nucleotides.

| Region | Time bin | Number of sequences | Number of haplotypes | Nucleotide diversity | Haplotype diversity |
| --- | --- | --- | --- | --- | --- |
| Asia | BLGM | 11 | 6 | 0.0164 | 0.8 |
|  | LGM | 3 | 2 | 0.015 | 0.67 |
|  | PLGM | 2 | 2 | 0.0187 | 1 |
| Urals | BLGM | 7 | 5 | 0.0153 | 0.9 |
|  | LGM | 2 | 2 | 0.015 | 1 |
|  | PLGM | 4 | 3 | 0.0137 | 0.83 |
| Switzerland | BLGM | 4 | 4 | 0.0104 | 1 |
|  | LGM | 11 | 4 | 0.0093 | 0.6 |
|  | PLGM | 57 | 29 | 0.0159 | 0.95 |
