# Supplementary material for: Ancient mtDNA diversity reveals specific population development of wild horses in Switzerland after the Last Glacial Maximum
Source: PLoS One. 2017 May 24;12(5):e0177458. doi: 10.1371/journal.pone.0177458 (PMC5443500; doi:10.1371/journal.pone.0177458)
Supplement: S9 Table — Lower triangle: FST values, upper triangle: p values. Comparable populations are boxed, significant FST values are in bold. Swiss/Swabian samples: dataset 2. (DOCX) [file pone.0177458.s013.docx]

S9 Table: F_ST_ values of Eurasian Pleistocene horses. Lower triangle: F_ST_ values, upper triangle: *p* values. Comparable populations are boxed, significant F_ST_ values are in bold. Swiss/Swabian samples: dataset 2.

| **A** | Asia BLGM | Asia LGM | Asia PLGM | Ural BLGM | Ural LGM | Ural PLGM | Swiss BLGM | Swiss LGM | Swiss PLGM |
| --- | --- | --- | --- | --- | --- | --- | --- | --- | --- |
| Asia BLGM | - | 0.3 | 0.6 | 0.5 | 0.6 | 0.5 | 0.7 | **0.0008** | **0.03** |
| Asia LGM | 0 | - | 0.4 | 0.4 | 0.8 | 0.3 | 0.4 | **0.03** | 0.09 |
| Asia PLGM | 0.068 | 0.055 | - | 0.3 | 1 | 0.3 | 0.07 | **0.05** | 0.1 |
| Ural BLGM | 0 | 0.01 | 0.103 | - | 0.4 | 0.6 | 0.7 | **0.005** | 0.3 |
| Ural LGM | 0 | 0 | 0 | 0 | - | 0.1 | 0.2 | **0.01** | 0.1 |
| Ural PLGM | 0 | 0.076 | 0.144 | 0.032 | 0.255 | - | 1 | 0.2 | 0.8 |
| Swiss BLGM | 0 | 0.031 | 0.203 | 0 | 0.203 | 0 | - | **0.02** | 0.5 |
| Swiss LGM | **0.233** | **0.383** | **0.387** | **0.265** | **0.51** | 0.162 | **0.297** | - | **0.0003** |
| Swiss PLGM | **0.05** | 0.11 | 0.142 | 0.01 | 0.163 | 0 | 0 | **0.167** | - |
